# Biodiversity and Biogeography of Chthamalid Barnacles from the North-Eastern Pacific (Crustacea Cirripedia)

**DOI:** 10.1371/journal.pone.0149556

**Published:** 2016-03-09

**Authors:** Benny K. K. Chan, H. -N. Chen, P. R. Dando, A. J. Southward, E. C. Southward

**Affiliations:** 1 Biodiversity Research Center, Academia Sinica, 128 section 2, Academia Road, Taipei, 115, Taiwan, ROC; 2 Institute of Ecology and Evolutionary Biology, National Taiwan University, Taipei, Taiwan, 10617, ROC; 3 Marine Biological Association of the U.K., The Laboratory, Citadel Hill, Plymouth, Pl 2PB, United Kingdom; The University of Sydney, AUSTRALIA

## Abstract

The biogeography and ecology of the species of Chthamalus present on the west coast of America are described, using data from 51 localities from Alaska to Panama, together with their zonation on the shore with respect to that of other barnacles. The species present were C. dalli, Pilsbry 1916, C. fissus, Darwin, 1854, C. anisopoma Pilsbry 1916 and four species in the C. panamensis complex. The latter are C. panamensis Pilsbry, 1916, C. hedgecocki, Pitombo & Burton, 2007, C. alani nom. nov. (formerly C. southwardorum Pitombo & Burton, 2007) and C. newmani sp. nov.). These four species were initially separated by enzyme electrophoresis. They could only be partially separated by DNA bar coding but may be separated using morphological characters.

## Introduction

Small chthamalid barnacles are a major world-wide component of the rocky intertidal zone of tropical and sub-tropical shores, though only a few species penetrate into temperate latitudes. Darwin [1] described *Chthamalus fissus* from California, but otherwise it was to be assumed that the rest of the Pacific was inhabited by “varieties” of the then world-wide species *Chthamalus stellatus*. As more specimens reached the museums, Pilsbry [2] distinguished *C*. *dalli* of the north-east Pacific temperate region, leaving *C*. *fissus* restricted to the warm-temperate region of southern California. Pilsbry [2] also described a curiously asymmetrical species, *C*. *anisopoma* from the Gulf of California, (Sea of Cortez) and two new species from the Panama Canal Zone. One, *C*. *imperatrix*, has now been assigned to *Microeuraphia*, but the other, *Chthamalus panamensis*, has caused severe problems to naturalists because of its apparent wide distribution from Baja California to Peru and to the difficulty of separating it from *C*. *fissus* and *C*. *dalli*. The apparent lack of distinct morphological characters to separate *C*. *fissus* and *C*. *panamensis*, especially of differences in cirri, mouth-parts and their setation, led Southward & Newman [3] to suggest that *C*. *fissus*, *C*. *panamensis* and the Caribbean form of *C*. *bisinuatus* (since distinguished as *C*. *proteus*, Dando & Southward [4]) should be regarded as varieties of a single amphiamerican neotropical species. Such a distribution was envisaged to be a result of the way in which the Caribbean and the Isthmus of Panama were believed to have been formed in the Jurassic Epoch, with final closure of the Isthmus as recently as the Pliocene.

There was a lack of information on the distribution of chthamalids along the coast of the Tropical Eastern Pacific (TEP) to assess the validity of the amphiamerican species hypothesis. This led Dr W.A. Newman, of Scripps Institution of Oceanography, to organise a cruise of R.V. ‘Alpha Helix’, TEPE78, to examine the shores of the TEP between Panama and northern Mexico in April 1978. The first breakthrough occurred as a result of enzyme electrophoresis studies on the cruise samples by Hedgecock [5]. The results confirmed the uniqueness of *C*. *anisopoma* and *C*. *dalli* and showed that the *C*. *fissus* group contained 3 sibling species. One northern species, called *C*. *‘fissus* SD’ from San Diego, was thought to be the true *C*. *fissus*. Another species, *C*. *‘fissus* BC’, was found on the Mexican coast at Bahia Chamela, Jalisco and Isla Sacrificios, Oaxaca, with a single specimen being found at San Diego, California and 4 specimens in the Golfo de Fonseca, Honduras. A southern species, called *C*. *‘fissus* FB’ was found at Farfan Beach, Panama and was the dominant species in the Golfo de Fonseca, Honduras. Hedgecock [5] thought that the FB form was probably *C*. *panamensis*.

Southward and Newman (unpublished notes), after examining these specimens, did not consider that this division explained all the morphological variation observed along the coast. Dando & Southward[6], again using enzyme electrophoresis, found that there were 6 species of the *Chthamalus fissus* group in the region between lower California and Panama: *C*. *fissus*, *C*. *anisopoma* and two pairs of sibling species. The latter were related to, and included, *C*. *panamensis* Pilsbry. All four of the sibling species could be separated by morphology. This finding was also reported by Southward [7] and Dando [8].

Laguna [9] also had access to the TEPE78 collections and described in his thesis the distribution of barnacles in the TEP. He referred to Hedgecock’s “Mexican type” of *Chthamalus* as an undescribed species with a range from Mazatlan to Honduras. Laguna [9] also found a “Cortezian type” that co-existed with *C*. *anisopoma* in the Gulf of California and on the west coast of Baja California. Laguna also noted that *C*. *panamensis s*.*s*. ranged from the Golfo de Fonseca, Honduras to the Gulf of Guyaquil, Ecuador. In a later paper, Laguna [10] introduced the informal names *Chthamalus “mexicanus”* and *Chthamalus “cortezianus”*, both of which he related to Hedgecock [5], although the latter author only found one new species in Mexican waters. Subsequent authors have quoted these two informal names, but have confused the species to which they refer; for example, Meyers *et al*. [11] equate *C*. *“mexicanus”* with *C*. *hedgecocki* on p. 77, but with *C*. *southwardorum* on p. 84 of their paper.

Pitombo & Burton [12] published the first formal descriptions of two new TEP species: *Chthamalus hedgcocki* Pitombo & Burton, 2007, from type locality Punta Camaron, Mazatlan, Sinaloa, Mexico, range from Punta Lobos, Baja California Sur to Acapulco, Guerro, Mexico; and *Chthamalus southwardorum* Pitombo & Burton, 2007, from type locality San Christovan River estuary, San Blas, Nayarit, Mexico, range from the Gulf of California to Peru. They equated *C*. *hedgecocki* to the BC form of Hedgecock [5] and *C*. *southwardorum* to the FB form of Hedgecock [5]. Pitombo & Burton [12] thought that *C*. *hedgecocki* and *C*. *panamensis* were likely to be one of the pairs of sibling species referred by Dando and Southward and that the second pair might be formed by a further division of *C*. *southwardorum* or by another cryptic, undescribed species.

Wares and his co-workers [11, 13, 14] collected new material of *Chthamalus* spp. from various localities in Mexico, north of 19°, and in Costa Rica and Panama. They inferred patterns of speciation from DNA sequence studies of mitochondrial CO1 and ribosomal 16S and presented evidence that *C*. *southwardorum* existed in genetically-distinct southern and northern clades. Meyers *et al* [11] calculated the timing of the isolation of the northern and southern *Chthamalus* populations, either side of the Central American Gap near the Gulf of Tehuantepec, as at least 315,000 years BP. They additionally presented evidence of a more recent northward migration of *C*. *panamensis* into Mexico starting 160,000 years BP.

The current descriptions of species in the *panamensis* complex [2, 12] do not cover all the four species known to be present, nor do they adequately describe the range of intraspecific variation. Thus it is still difficult to separate the species in the *C*.*’panamensis’* complex. In the present study we describe in detail the morphology of *Chthamalus* species in the North Eastern Pacific, including *C*. *anisopoma* and the four species in the *panamensis* complex, relate this to the results of enzyme electrophoresis and DNA barcoding, and provide keys to separate all the species of *Chthamalus* known from the North Eastern Pacific. The one new species described is the “southern” clade of *C*. *southwardorum*. We discuss the relationships between the species in the *C*. *fissus* group, give guidance on the best characters for identification on the shore and outline the geographical distribution and zonation of *Chthamalus* species from Alaska to Panama.

## Materials and Methods

### Sample Collections

**Fig 1:**
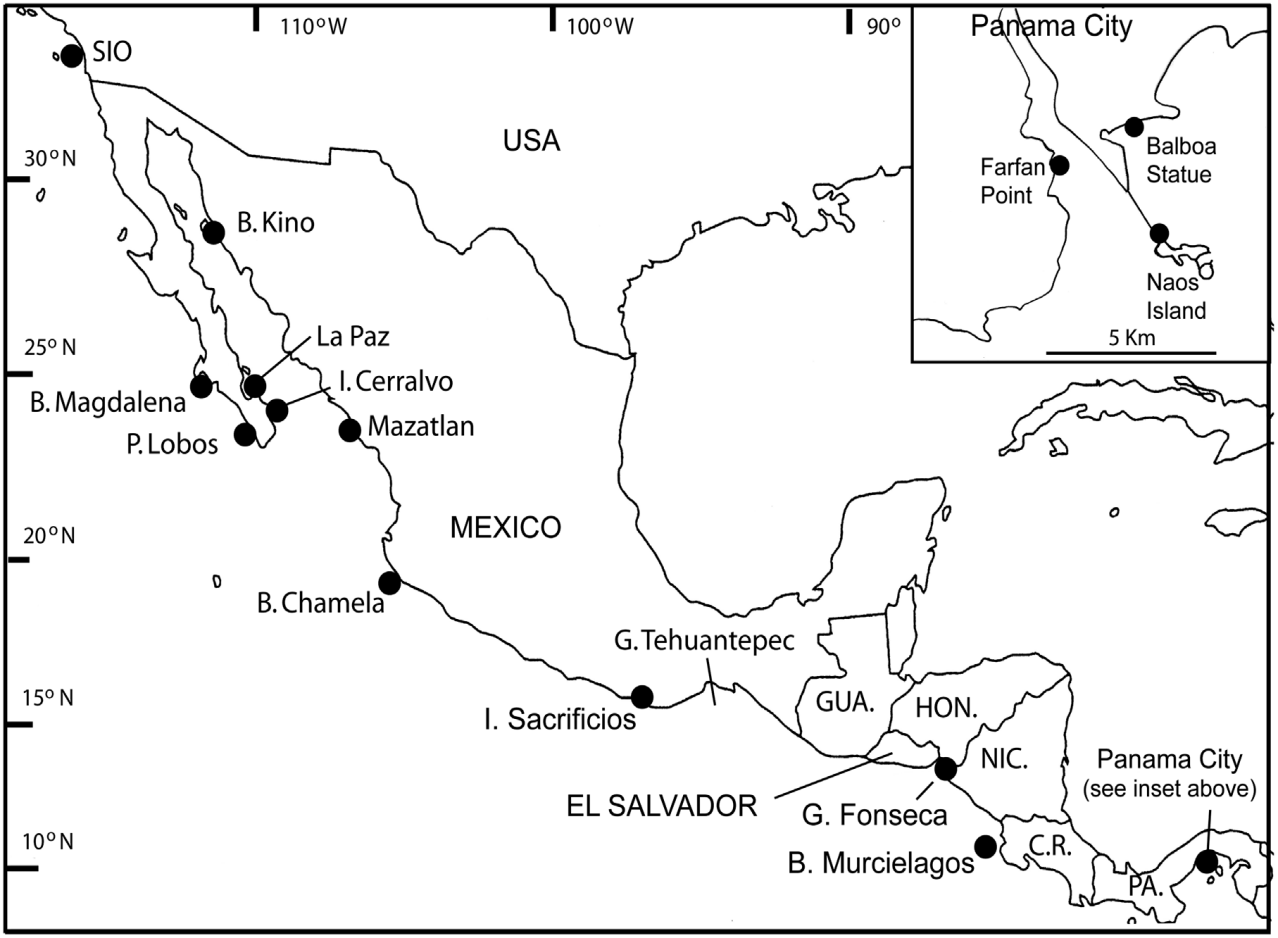
Sampling sites for specimens used in species identification, descriptions and other key locations mentioned in the text. See Table 1 for detailed site locations and analysis. Middle America outline map courtesy Arizona Geographic Alliance http://geoalliance.asu.edu.

Most of the barnacle samples were collected on the TEP *Alpha Helix* Expedition, during March-April, 1978 and in shore-based collections immediately after the expedition and in October 1978 by AJS (Fig 1). These collection stations ranged from 32° 52.27' N in California to 8° 56.37'N in Panama. Subsequently samples were collected from Naos Island (formerly Quarantine Island) Panama (8°55´ N), the type locality for *Chthamalus panamensis* Pilsbry, and from Avenida Balboa, Panama City (8°58´ N) by Dr. P. Glynn in 1979 and sent alive by air. Specimens from La Shalpa, El Salvador (13.33° N) were collected by K. de Reimer in 1978. Samples from Isla Sacrificios (TEPE78-14, 15°40.8´ N 96° 14.6´ W) and Farfan Point were sent frozen, by air freight, by Dr. D. Hedgecock. Preserved samples from these sites were also available for morphological examination. Living samples from British Columbia were collected by P.R. Dando in 1980 and 2013. Slide-mounted and dried specimens, collected by Dora P. Henry (University of Washington) from Mexico, Guatemala, Costa Rica and Panama were examined by AJS. They are now housed at the National Museum of Natural History, Smithsonian Institution, USA The type specimens of *Chthamalus panamensis* Pilsbry were loaned by the Academy of Natural Sciences of Drexel University, Philadelphia. Type and paratypes of the new species described in the present study have been deposited in the Natural History Museum, London (NHMUK) and the Biodiversity Research Museum, Academia Sinica, Taiwan (ASIZCR). The collections used for morphological descriptions, allozyme and mitochondrial DNA studies are listed in Table 1. The descriptions of sites where specimens were examined for details of species distribution and zonation, also where collections were made for allozyme studies of *C*. *dalli* are given in S1 Table.

**Table 1:** Stations where specimens were collected for comparative studies of the species.

TEPE no.	Date	Lat. °N	Long. °W	Place	Details	Morphology	Flap photos	Allozymes	CO1
78–1	30/03/1978	8° 56.1'	79° 34.2'	Farfan Beach, Farfan Point, Panama	Rock platform & vert. face	*	*	*	*
78-7B	03/04/1978	10° 53'	85° 54'	South side of Key Point (= Pta Santa Helena), Murcilago,Costa Rica	Upper shore hard rock; lower shore stones, tide pools	—	*	—	—
78–14	07/04/1978	15° 41'	96° 13.9'	Isla Sacrificios, Oaxaca, Mexico	Exposed rocks and more sheltered boulders	*	—	*	—
78–21	12/04/1978	19° 35'	105° 08.5'	Chamela, Jalisco, Mexico	Rocks N side of sand beach, good barnacle zone	—	*	—	—
78–24	14/04/1978	24° 08'	109° 49'	Isla Cerralvo, Baja California Sur, Mexico	Rocks between Montana Rock and Pt Piedras Gordas	*	*	—	—
78–25	14/04/1978	24° 08'	109° 48'	Isla Cerralvo, Baja California Sur, Mexico	Montana Rock, exposed low rocks	—	*	—	—
Land based *Alpha Helix* stations after leaving the ship									
78–34	17/04/1978	23° 24.5'	110° 14'	Point Lobos, Todos Santos, Baja California Sur	Rocks at S end long sandy beach. Very wave exposed	*	*	—	*
78–35	18/04/1978	24° 09'	110°15'	La Paz, between end of Promenade and Hotel.	Stones, boulders	*	*	—	—
78–36	19/04/1978	23° 11.7'	106° 25.5'	Ocean front, Mazatlan, Sinaloa, Mexico.	Boulders very wave exposed	*	—	—	—
Other Samples									
AJS 1970	31/10/1970	8° 57.9'	79° 32'	Balboa Statue, Panama City, Panama	Rock ledges	—	*	—	*
AJS 2	03/11/1978	24° 13'	110° 8.6'	La Paz Bay,	Rocks ca 5 km N of La Paz	*	*	*	*
AJS 3	04/11/1978	23° 24.5'	110° 14'	Point Lobos,Todos Santos, Baja California Sur, Mexico	Rocks at S end long sandy beach, very wave-exposed	*	*	*	—
PWG 1	7/2/1979	8° 55.1'	79° 32.1'	Naos Island, Panama	Rocks NW of causeway	*	—	*	*
PWG 2	?/06/1979	8° 55.1'	79° 32.1'	Naos Island, Panama	Rocks NW of causeway	—	*	*	*
PWG 3	?/06/1979	8° 57.9'	79° 32'	Avenida Balboa, Panama City, Panama	Rocky intertidal	—	*	*	*

For further details see S2 Table

TEPE = Tropical East Pacific Expedition intertidal barnacle sites (RV *Alpha Helix*)

Collected by: AJS = AJ Southward, PWG = PW Glynn.

* = data acquired

Permission to work and collect in the coastal waters of Mexico, El Salvador, Guatemala, Honduras, Costa Rica and Panama during the Tropical Eastern Pacific Expedition 1978 cruise of the R.V. Alpha Helix was obtained by Scripps Institution of Oceanography. There were no restrictions on collecting intertidal barnacles at the date of collection at any site from which they were removed.

### Nomenclature Acts

The electronic edition of this article conforms to the requirements of the amended International Code of Zoological Nomenclature, and hence the new names contained herein are available under that Code from the electronic edition of this article. This published work and the nomenclatural acts it contains have been registered in ZooBank, the online registration system for the ICZN. The ZooBank LSIDs (Life Science Identifiers) can be resolved and the associated information viewed through any standard web browser by appending the LSID to the prefix “http://zoobank.org/”. The LSID for this publication is: urn:lsid:zoobank.org:pub:0E2F984F-D577-431A-B1FD-DB10B370D383. The electronic edition of this work was published in a journal with an ISSN, and has been archived and is available from the following digital repositories: PubMed Central, LOCKSS, PlymSEA

### Morphological Examination

Barnacles were dissected and the opercular plates and the arthropodal characters, including the cirri and mouth parts, were examined by light microscopy. Samples of *C*. *panamensis* and an undescribed species in Panama were further investigated using scanning electron microscopy, methods following Chan *et al*.[15]. Setal terminology follows Chan *et al*.[16, 17].

### Allozyme Electrophoresis

Living specimens, from Panama, California, the Baja Peninsula and British Columbia, were maintained at Plymouth in a “tidal” tank at 25°C and fed fresh plankton twice a week. Both the live and frozen barnacles were treated as described by Dando *et al*. [18]. Enzymes were extracted by soaking the animals, for 2–3 h at 2°C, in a buffer containing 200 mM triethanolamine, 2 mM disodium EDTA, 1 mM 2-mercaptoethanol and 0.25% (v/v) 1-phenoxy-2-propanol, adjusted to pH 7–0 at 20°C. This left the shells and arthropodal structures available for examination. Enzymes were separated by vertical starch-gel electrophoresis using the buffers listed in Table 2 and staining procedures previously described [4, 18]. Allozyme mobilities were measured with respect to horse-spleen ferritin, given a nominal mobility of 100, which was added to the samples as a visible marker. The enzymes used were mainly associated with the glycolytic cycle and had previously proved useful in separating species of *Chthamalus* in cyprids (P. R. Dando, unpublished studies) and newly metamorphosed individuals [19], as well as in adult stages [6, 18].

**Table 2:** Enymes compared in the present study and electrophoresis conditions.

Enzyme	Abbreviation	Enzyme Catalogue no.	Buffer	Additive
Malate dehydrogenase	Mdh1, Mdh2	1.1.1.37	1	NAD
Malic enzyme	Me	1.1.1.39	2	none
Phosphogluconatedehydrogenase (decarboxylating)	Pgdh	1.1.1.41	2	ATP
Glyceraldehydephosphate dehydrogenase	Gpdh	1.2.1.12	1	NAD
Superoxide dismutase	Sod	1.15.1.1	2	ATP
Hexokinase	Hk	2.7.1.1	2	ATP
Pyruvate kinase	Pk	2.7.1.40	2	FDP
Phosphoglycerate kinase	Pgk	2.7.2.3	2	ATP
Arginine kinase	Ark1, Ark2	2.7.3.3	2	ATP
Phosphoglucomutase	Pgm-1	2.7.5.1	2	FDP
Fructose-bisphosphate aldolase	Ald	4.1.2.13	1	NAD
Enolase	En	4.2.1.11	2	none
Glucosephosphate isomerase	Gpi	5.3.1.9	2	FDP
Mannosephosphate isomerase	Mpi	5.3.1.18	2	FDP

Buffers used: 1. 40 mM citric acid adjusted to pH 7.7 with N-(3-aminopropyl) diethanolamine for electrode compartments, 1 in 20 dilution for the gel. 2. 900 mM 2-amino-2-hydroxymethyl-propane-1, 3-diol, 500 mM boric acid, 20 mM EDTA pH 8.7. Additives added to the gel: 0.1 mM NAD, 1 mM ATP or 0.1 mM FDP

Specimens of *Chthamalus montagui* Southward, 1976 and *C*. *stellatus* were run side by side on the same gels with specimens of the taxa examined in this study. The mobilities for the enzymes in *C*. *montagui* and *C*. *stellatus*, examined in the course of this study, were within the ranges previously reported [18]. This comparison provided an additional check on the validity of the species and population comparisons based on allozyme mobilities. In addition, specimens of the taxa under study were compared with frozen specimens of *C*. *proteus* and *C*. *fragilis* Darwin, 1854 from St. Petersburg, Florida and with specimens of *C*. *dalli* from La Jolla and San Diego, California.

### Mitochondrial DNA Analysis

The samples used were preserved in 75% ethanol and examined by mitochondrial DNA analysis. Total genomic DNA was extracted from soft tissues using a Qiagen DNeasy blood and tissue kit (Hilden, Germany) following the manufacturer’s instructions. The mitochondrial COI gene was amplified using the universal forward primer LCO1490 [20] in combination with the reverse primer COI-R4 [21]. The amplification reaction mix contained 2 μl template DNA, 12 μl Taq DNA Polymerase Master Mix Red (1.5 mM MgCl_2_; Ampliqon, Denmark), 1 μM of each primer with ddH_2_O to a total volume of 20 μl. The PCR profile was as follows: 4 min at 94°C for initial denaturation, 30 or 35 cycles of 30 sec at 94°C, 30 sec at 48°C and 1 min at 72°C, and 5 min at 72°C for final extension. The concentrations of PCR products were first evaluated using gel electrophoresis. If the concentration of PCR products was too low for sequencing, a nested PCR was conducted. The PCR products from the primary amplification were diluted to 1% and used for a secondary PCR. The universal barcode primers LCO1490 and HCO2198 [20] were used for the nested PCR using the same conditions for the amplification reaction mix and PCR profile. The PCR products were sequenced on an ABI3730XL Genetic Analyzer with BigDye terminator cycle sequencing reagents (Applied Biosystems, Foster City, CA).

To compare the *Chthamalus* sequences with those from other regional studies, sequences from Wares *et al*. [14] and Deng & Hazel [22] were downloaded from GenBank (Table 3) for analysis. Sequences of *C*. *southwardorum*, *C*. *panamensis* and *C*. *hedgecocki* (Table 3) from Pitombo & Burton [12] were also used for comparison. All of the sequences were aligned using MUSCLE [23] implemented in MEGA 5.05 [24] using the default setting and adjusted visually. The best-fit nucleotide substitution model of COI was selected by jModelTest 0.1.1 [25].

**Table 3:** Source of COI DNA barcode sequences used in mitochondrial DNA analyses.

Species	Sample locality and reference	GenBank Number
*Chthamalus hedgecocki*	Todos Santos, Mexico. This study.	KU356704—KU356705
*Chthamalus hedgecocki*	Punta Camarón, Mazatlán, Sinaloa, Mexico. Pitombo & Burton 2007	*
*Chthamalus hedgecocki*	Punta da Mita, Baderas Bay, Nayarit, Mexico. Pitombo & Burton 2007	*
*Chthamalus hedgecocki*	Pie de La Cuesta, Acapulco, Guerrero, Mexico. Pitombo & Burton 2007	*
*Chthamalus hedgecocki*	Pie de La Cuesta, Acapulco, Guerrero, Mexico. Pitombo & Burton 2007	*
*Chthamalus “hedgecocki”*	Wares *et al*. 2009#	FJ857983-FJ857991
*Chthamalus panamensis*	Naos Island, Panama. This study.	KU356706—KU356708
*Chthamalus panamensis*	Punta da Mita, Baderas Bay, Nayarit, Mexico. Pitombo & Burton 2007	*
*Chthamalus panamensis*	Punta da Mita, Baderas Bay, Nayarit, Mexico. Pitombo & Burton 2007	*
*Chthamalus panamensis*	Punta Tenacatita, Jalisco, Mexico. Pitombo & Burton 2007	*
*Chthamalus panamensis*	Pie de La Cuesta, Acapulco, Guerrero, Mexico. Pitombo & Burton 2007	*
*Chthamalus panamensis*	Manoel Antonio, Quepos, Costa Rica. Pitombo & Burton 2007	*
*Chthamalus panamensis*	Cabo Matapalo, Peninsula de Osa, Costa Rica. Pitombo & Burton 2007	*
*Chthamalus panamensis*	Cabo Matapalo, Peninsula de Osa, Costa Rica. Pitombo & Burton 2007	*
*Chthamalus panamensis*	Cabo Matapalo, Peninsula de Osa, Costa Rica. Pitombo & Burton 2007	*
*Chthamalus “panamensis”*	Wares *et al*. 2009	FJ857949-FJ857982
*Chthamalus newmani*	Naos Island, Panama. This study.	KU356709—KU356712
*Chthamalus newmani*	Panama City, Panama. This study.	KU356713—KU356722
*Chthamalus newmani*	(*Chthamalus “southwardorum”* B).#Wares *et al*. 2009	FJ857992-FJ858000
*Chthamalus alani*	Mazatlan, Mexico. This study.	KU356723
*Chthamalus alani*	(*Chthamalus southwardorum*). Punta Camarón, Mazatlán, Sinaloa, Mexico. Pitombo & Burton 2007	*
*Chthamalus alani*	(*Chthamalus southwardorum*). San Blas, Nayarit, Mexico. Pitombo & Burton 2007	*
*Chthamalus alani*	(*Chthamalus southwardorum*). Punta da Mita, Banderas Bay, Nayarit, Mexico. Pitombo & Burton 2007	*
*Chthamalus alani*	(*Chthamalus southwardorum*). Punta da Mita, Baderas Bay, Nayarit, Mexico. Pitombo & Burton 2007	*
*Chthamalus alani*	(*Chthamalus “southwardorum”* A). Wares et al. 2009	FJ858001-FJ858020
*Chthamalus alani*	(*Chthamalus southwardorum*). Bahia Kino, Mexico. Deng & Hazel 2010	DQ538442-DQ538449
*Chthamalus anisopoma*		DQ538416
*Chthamalus dalli*	Pitombo & Burton 2007	*
*Chthamalus fissus*	Pitombo & Burton 2007	*

^#^ Wares et al. (2009) did not give site locations for their individual sequences and assigned the species names “through clade identity with type specimen sequences” from Pitombo and Burton (2007), without consideration of any morphological features.

Note that in the text, Wares et al. (2009) erroneously refer to *C*. *“southwardorum*” B as the northern clade (Wares, personal communication).

* Pitombo and Burton (2007) did not upload their sequences to the Genbank but included them in appendix 2 of their paper.

The genealogical relationships of the species were constructed using the Neighbour-joining (NJ) method with the Kimura-2 parameter nucleotide substitution model implemented in MEGA. *C*. *fissus*, *C*. *dalli* and *C*. *anisopoma* were selected as outgroups. The statistical support was tested with 1000 bootstrap pseudoreplicates. The evolutionary distances between sequence pairs were calculated with Kimura-2-parameter (K2P) models on MEGA.

## Results

### Species Descriptions

Order Sessilia Lamarck, 1818

Suborder Balanomorpha Pilsbry, 1916

Superfamily Chthamaloidea Darwin, 1854

Family Chthamalidae Pilsbry, 1916

Subfamily Chthamalinae Darwin, 1854

Genus *Chthamalus* Ranzani, 1817

urn:lsid:zoobank.org:act:D73B8CF6-9EBA-4B90-85CD-73F7D0C2E017

#### Diagnosis

Shell with 6 plates, rostrum and carina with radii, rostrolaterals without radii, carinolateral lacking, mandibles with 4 teeth, bases membranous, Cirri I-II maxillipedes, III-VI ctenopods.

#### Type species

*Chthamalus stellatus* (Poli, 1791)

### *Chthamalus alani* nom. nov.

urn:lsid:zoobank.org:act:4C460612-D362-49BA-9A20-F2895C4CE1F5

the authority for this species name change rests with BKK Chan

**Fig 2:**
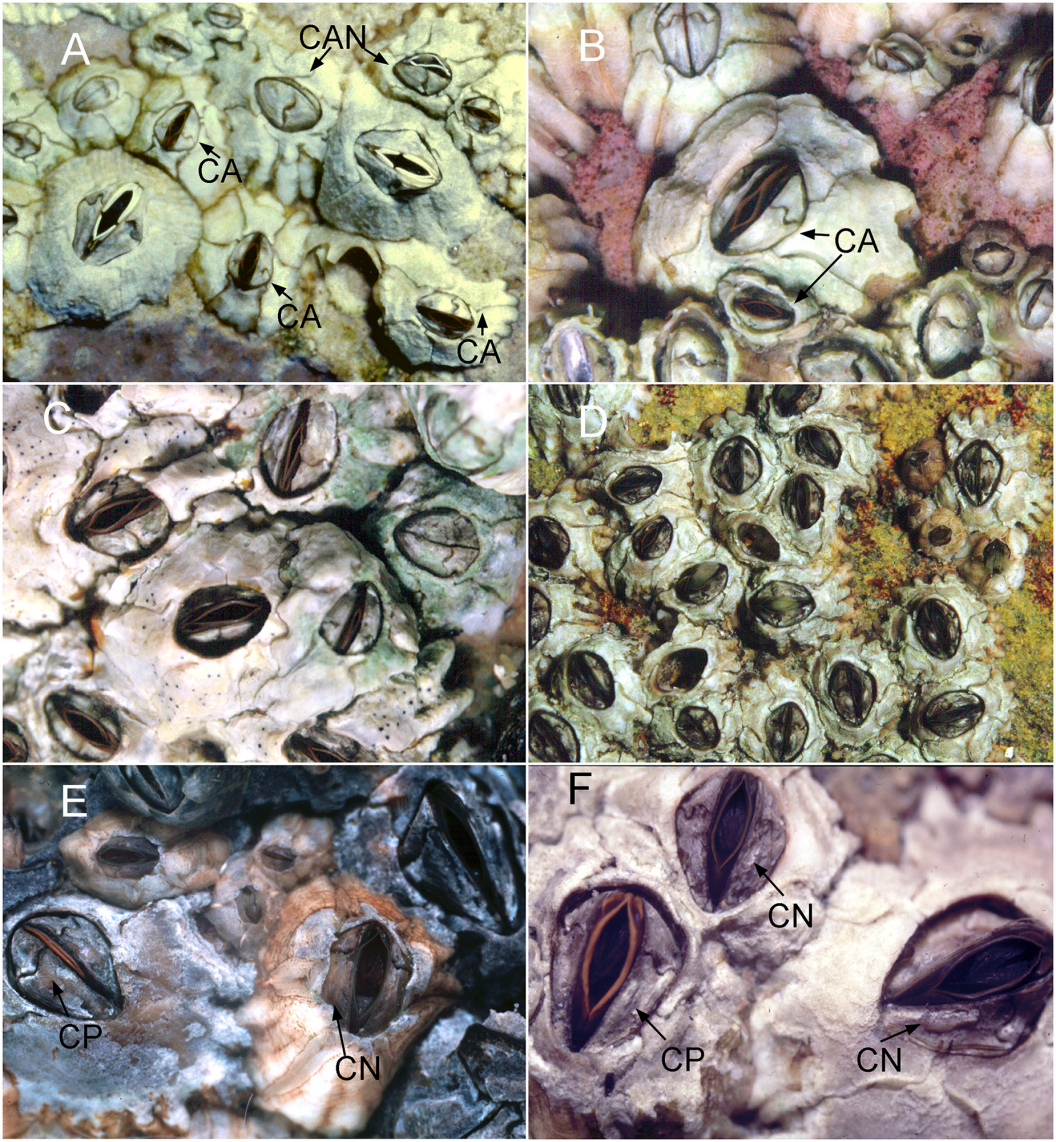
Live *Chthamalus* species photographed underwater. A, mixed populations of *C anisopoma* (CAN), with white-edged tergoscutal flaps, *Balanus inexpectatatus* (BI) with white tergoscutal flaps and *Chthamalus alani* nom. nov. (CA) with orange tergoscutal flaps (from La Paz, Baja California). B, *C*. *alani* showing orange tergoscutal flaps (from La Paz). C, *C*. *hedgcocki* showing orange tergoscutal flaps (from Point Lobos, Todos Santos, Baja California). D, *Chthamalus newmani* sp. nov. showing brown tergoscutal flaps (from Farfan Point, Panama). E, F, mixed population of *C*. *panamensis* (CP) showing orange tergoscutal flaps and *C*. *newmani* (CN) showing brown tergoscutal flaps (E, Balboa Statue. Panama City and F, Naos Island).

**Fig 3:**
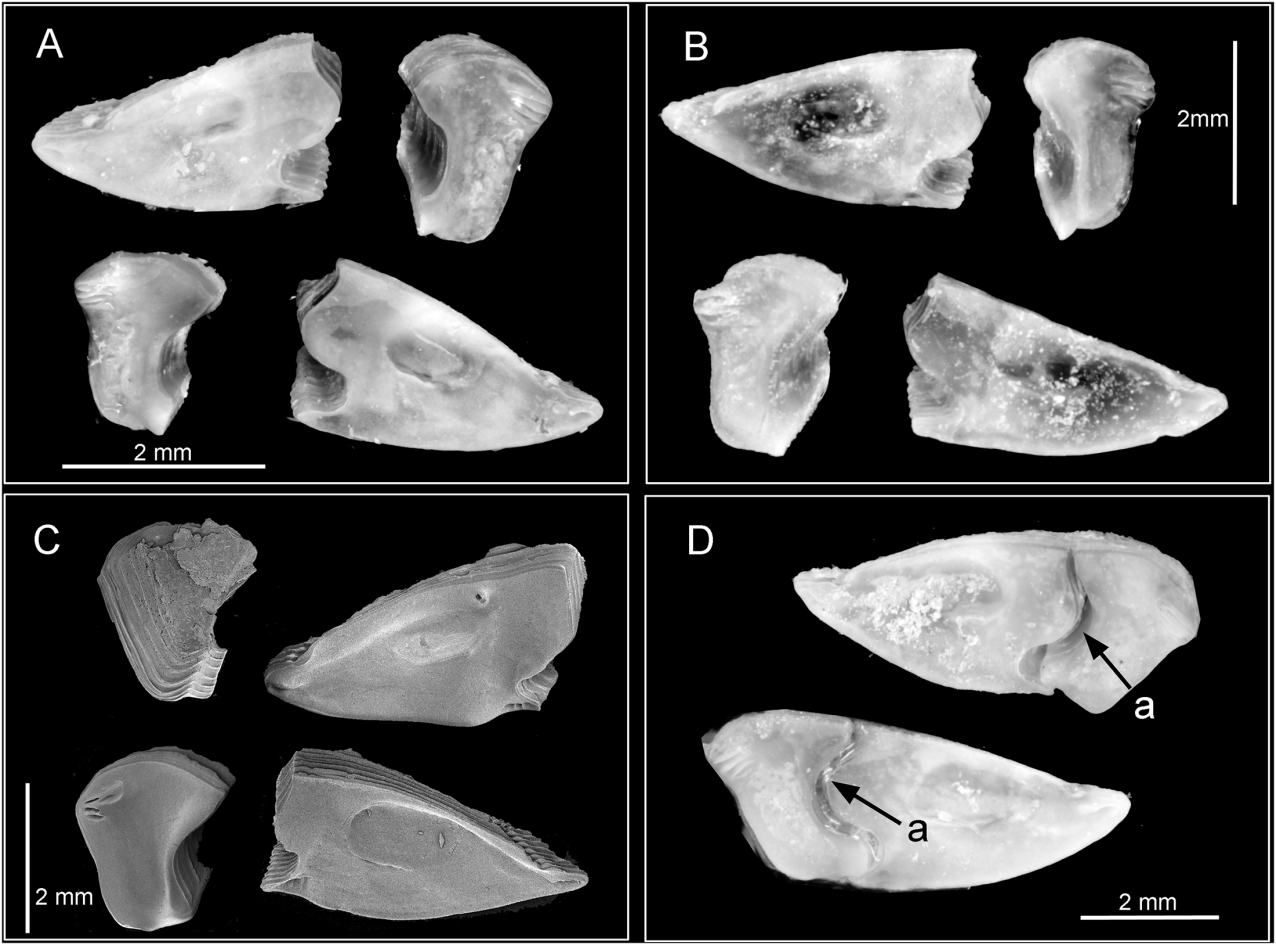
*Chthamalus alani* nom. nov. A, B, light stereomicroscopy showing inner side of paired scutum and tergum, La Paz specimen. C, scanning Electron Micrograph (SEM) showing the inner side of scutum and tergum, Mazatlan specimen. D, paired articulated scutum and tergum, a La Paz specimen, showing the oblique articulation of the plates (a).

**Fig 4:**
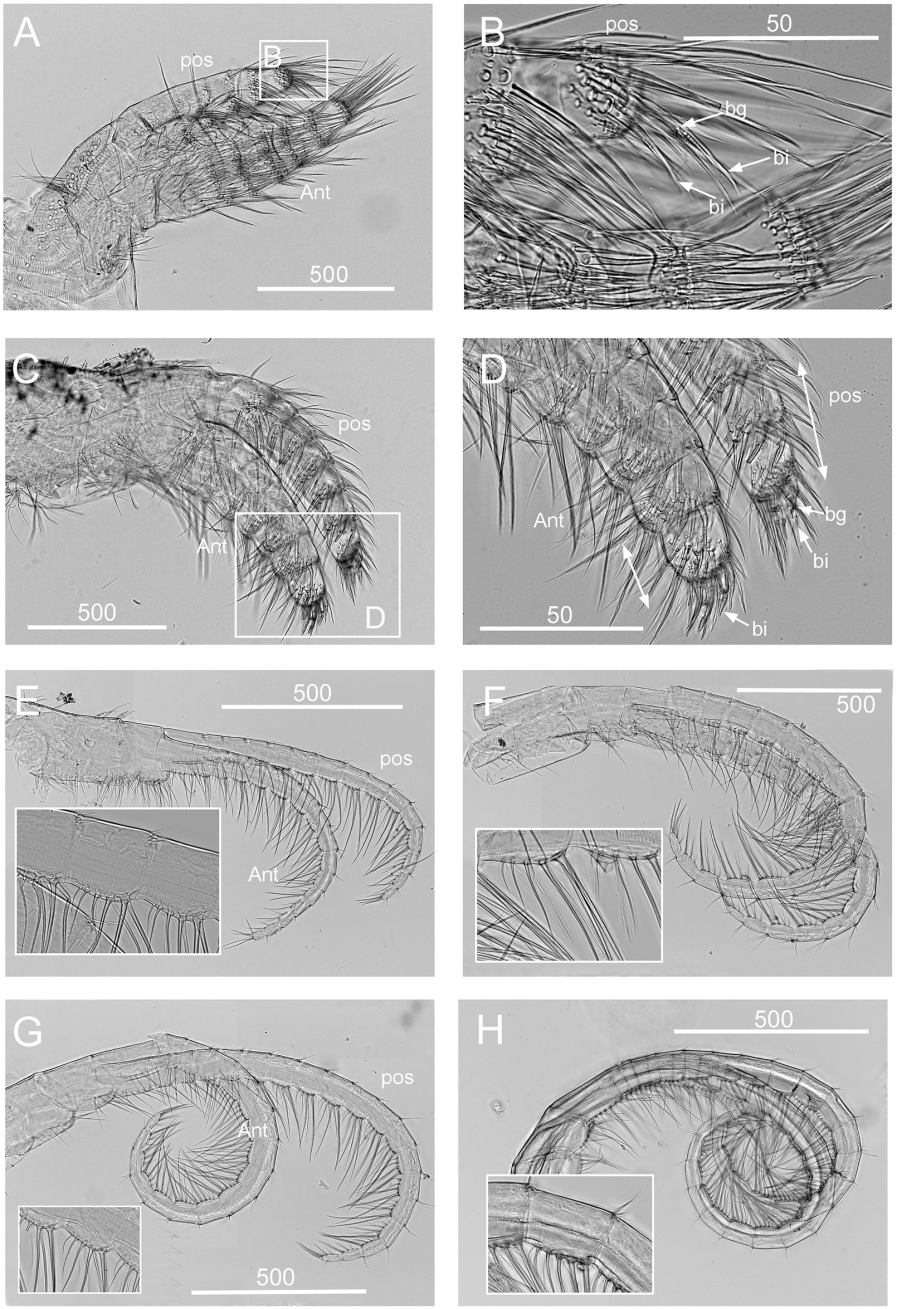
*Chthamalus alani* nom. nov. Matzalan, Mexico. A, B, cirrus I, showing the presence of bidenticulate setae (bi) with or without basal guards (bg), enlarged in B. C, D, cirrus II, showing the presence of bidenticulate setae (bi) with basal guards (bg) on the first few segments (indicated by double end arrows) in D. E, cirrus III. F, cirrus IV. G, cirrus V. H, cirrus VI. Intermediate segments of anterior rami of cirri III-VI are shown as inserts in E-H. Scale bars in μm.

**Fig 5:**
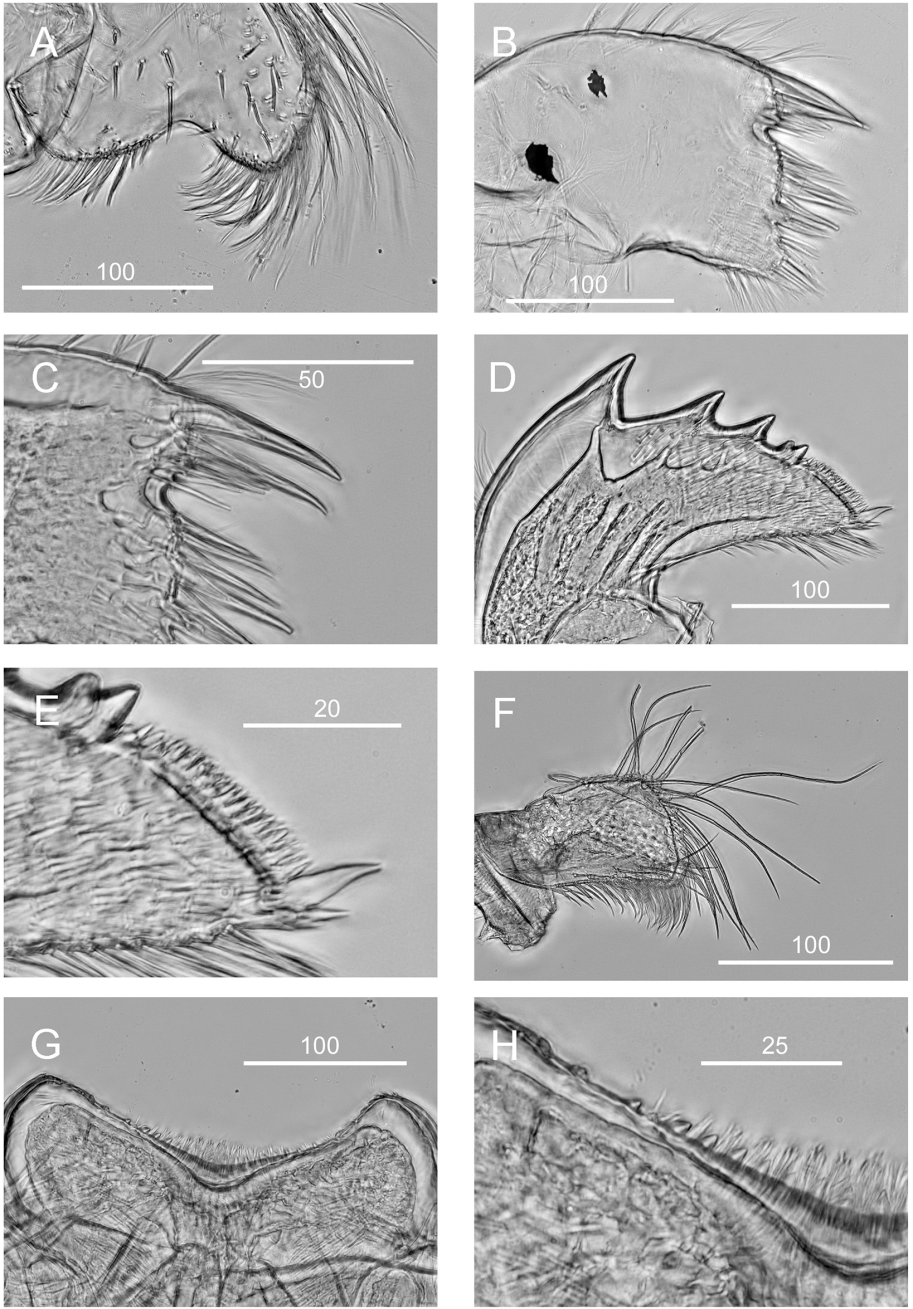
*Chthamalus alani* nom. nov. Matzalan, Mexico. A, maxilla. B, maxillule. C, magnified view showing the notch region of maxillule. D, mandible. E, fourth tooth, pecten and inferior angle of mandible. F, mandibular palp. G, labrum. H, cutting margin of labrum showing the fine teeth. Scale bars in μm.

Figs 2A and 2B and 3–5

*Chthamalus southwardorum* Pitombo & Burton, 2007: 9, figs 9–14 [12]

*Chthamalus southwardorum* A.—Wares *et al*., 2009: Table 1. [14]

#### Etymology

Pitombo & Burton [12] stated explicitly that *Chthamalus southwardorum* is named “in honor of Dr A.J. Southward, for his extensive contribution to the knowledge of chthamalids and his careful collecting during the Tropical eastern Pacific Expedition”. Unfortunately, the suffix *-orum* indicates “for men or for man (men) and woman (women) together” (ICZN, 4^th^ edition, 1999 Article 31.1.2). Since there is clear evidence of a *lapsus calami* in the original publication (ICZN ART 32.5.1) the name *C*. *southwardorum* is not admissible under ICZN Art. 21.13 and has to be treated as an incorrect original spelling that should be corrected to *southwardi*, taking the authorship and date of Pitombo & Burton [12]. However, *Chthamalus southwardi* Pitombo & Burton, 2007 is a junior primary homonym of *Chthamalus southwardi* Poltaruka, 2000 and therefore an invalid name (ICZN Art 57.2) and so a new replacement name is required for *C*. *southwardorum*. In the present study, I (BKKC) rename *C*. *southwardorum* as *Chthamlaus alani*, again in honour of Prof. Alan Southward, but using his first name, for his contributions to barnacle distribution, ecology and taxonomy.

#### Specimens examined

AJS-2, 10 specimens, high intertidal shores, La Paz Bay, 24° 13' N, 110° 18.6' W, 3 Nov 1978; AJS-3, 7 specimens, intertidal shores, Point Lobos, Todos Santos, Baja California Sur, Mexico, 23° 24.8' N, 110° 57' W, 4 Nov 1978; TEPE78-36, 10 specimens, Ocean Front, Mazatlan, Sinaloa, Mexico, exposed rocky shores with large boulders, 23° 11.7' N, 106° 25.5' W, 19 April 1978.

#### Diagnosis

*Chthamalus* with cirrus I having bidenticulate setae with or without basal guards on the distalmost segment of the posterior ramus and cirrus II having bidenticulate setae with basal guards on the two distalmost segments of the posterior ramus and the distalmost segment of the anterior ramus. Tergoscutal flaps orange-brown in colour when alive. COI sequence (Gene Bank Number KU356723).

#### Description

Shell depressed, sutures between plates visible (Fig 2A and 2B). External surface white and smooth, sheath white, with ribs at the basal region, not extending to the apex (Fig 2A and 2B). Internal colour of shell white. Orifice oval, scutum and tergum articulated with a marked angle (Figs 2A and 2B and 3D). Tergoscutal flaps orange-brown when alive (Fig 2A and 2B), colour fades out when preserved in ethanol or formalin. Scutum triangular, basal margin about twice as long as tergal margin (Fig 3). Tergal margin with a wide and elevated articular ridge, ridge extending beyond the tergal margin, articular furrow wide. Occludent margin straight, without teeth. Adductor muscle pit oval and deep, not extending to the basal margin. Lateral depressor muscle scar deep and smooth (Fig 3). Tergum trapezoid, external surface smooth near the apex, basal region with growth lines or striations, spur wide and not obvious, basal margin with 4 rostral depressor muscle crests (Fig 3).

Segment counts on cirri I-III were based on five specimens collected from Mazatlan, Mexico (TEPE-78-36). Segment counts on cirri IV-VI were based on a single specimen from Mazatlan, Mexico (Table 4). Cirrus I: posterior ramus 5 to 6-segmented (Fig 4A), the distalmost segment bears bidenticulate setae with or without basal guards (Fig 4B), serrulate type setae common on all segments of the rami, anterior ramus 6 to 8, serrulate type, setae common on all segments (Fig 4A, Table 4). Cirrus II: posterior ramus 4 to 7-segmented, the two distalmost segments bear bidenticulate setae with basal guards, anterior ramus 5- or 6-segmented, one or two of the distalmost segments have bidenticulate setae with basal guards, serrulate type setae common on both rami (Fig 4C and 4D, Table 4). Cirrus III: anterior and posterior rami similar in length (anterior and posterior rami length ratio = 0.9 ± 0.06), posterior ramus 11 to 16-segmented, anterior ramus 11 to 16-segmented (Fig 4E, Table 4). Cirrus IV: both anterior and posterior rami 16-segmented (Fig 4F). Cirrus V: posterior ramus 18-segmented, anterior ramus 19-segmented (Fig 4G). Cirrus VI: posterior ramus 18-segmented, anterior ramus 19-segmented (Fig 4H). Intermediate segments of both rami on cirri III-VI bear two pairs of long serrulate setae and three pairs of shorter simple setae (Fig 4E–4H).

**Table 4:** Segment counts (n = 5 individuals) of *Chthamalus* in the Tropical Eastern Pacific waters.

	*C*. *hedgecocki*	*C*. *anisopoma*	*C*. *alani*	*C*. *panamensis*	*C*. *newmani*
Cirrus I: No. of segments in posterior ramus	5–7	6–7	5–6	5–7	6–7
Cirrus I: No. of segmentsin anterior ramus	7–9	7–8	6–8	6–8	8–9
Cirrus I: No. of segments in posterior ramus with bidenticulate setae	0 ± 0	1	1	0	1
Cirrus II: No. of segments in posterior ramus	5–6	5	4–7	5–7	5–7
Cirrus II: No. of segments in anterior ramus	5–6	6–7	5–6	6–8	7–8
Cirrus II: No. of segments in posterior ramus with bidenticulate setae with basal guards	1–2	4	2	1	3
Cirrus II: No. of segments in anterior ramus with bidenticulate setae with basal guards	1	2	1–2	1	2
Cirrus III: No. of segments in posterior ramus	14–17	13–14	11–16	12–16	17–19
Cirrus III: No. of segments in anterior ramus	13–19	13–19	11–16	16–22	18–23
Cirris III, anterior/posterior length ratio	0.9–1.0	0.8–1.2	0.8–0.9	1.4–1.5	0.9–1.2

Note: cirrus I of *C*. *anisopoma* has bidenticulate setae without basal guards, whilst cirrus I of *C*. *alani* nom. nov. and *C*. *newmani* sp. nov. has bidenticulate setae with or without basal guards.

Maxilla bilobed, serrulate setae on apex of both lobes and on superior margin (Fig 5A). Maxillule notched, two large setae above notch, notch small, 8 setae on cutting margin below notch, inferior angle protuberant with bundles of simple setae (Fig 5B and 5C). Mandible has 4 major teeth, fourth tooth bidenticulate, pecten with 16 small teeth, inferior angle tipped with two pointed teeth (Fig 5D and 5E). Mandibular palp rectangular, serrulate setae on all margins (Fig 5F). Labrum concave, with 6–7 small teeth on both sides of the cutting edge (Fig 5G and 5H).

Penis without basi-dorsal point.

#### Distribution

Northern limit probably at about 29° N inside the Gulf of California and 24° 40' N on the Pacific coast; southern limit unknown, but probably north of 15° N. The holotype and paratypes of *C*. *southwardorum* Pitombo & Burton, 2007 were collected in the San Cristovan river estuary, San Blas, Nayarit, Mexico (21° 30' 54'' N, 105° 15' 53'' W), attached to an oyster shell, low midlittoral zone. Pitombo and Burton stated that the distribution of *C*. *southwardorum* was from Bahía Magdalena, Baja California Sur, Mexico to Puerto Chicama, Peru, but they did not distinguish it from the new species *C*. *newmani* described below.

### *Chthamalus anisopoma* Pilsbry, 1916

Figs 2A and 6–8

**Fig 6:**
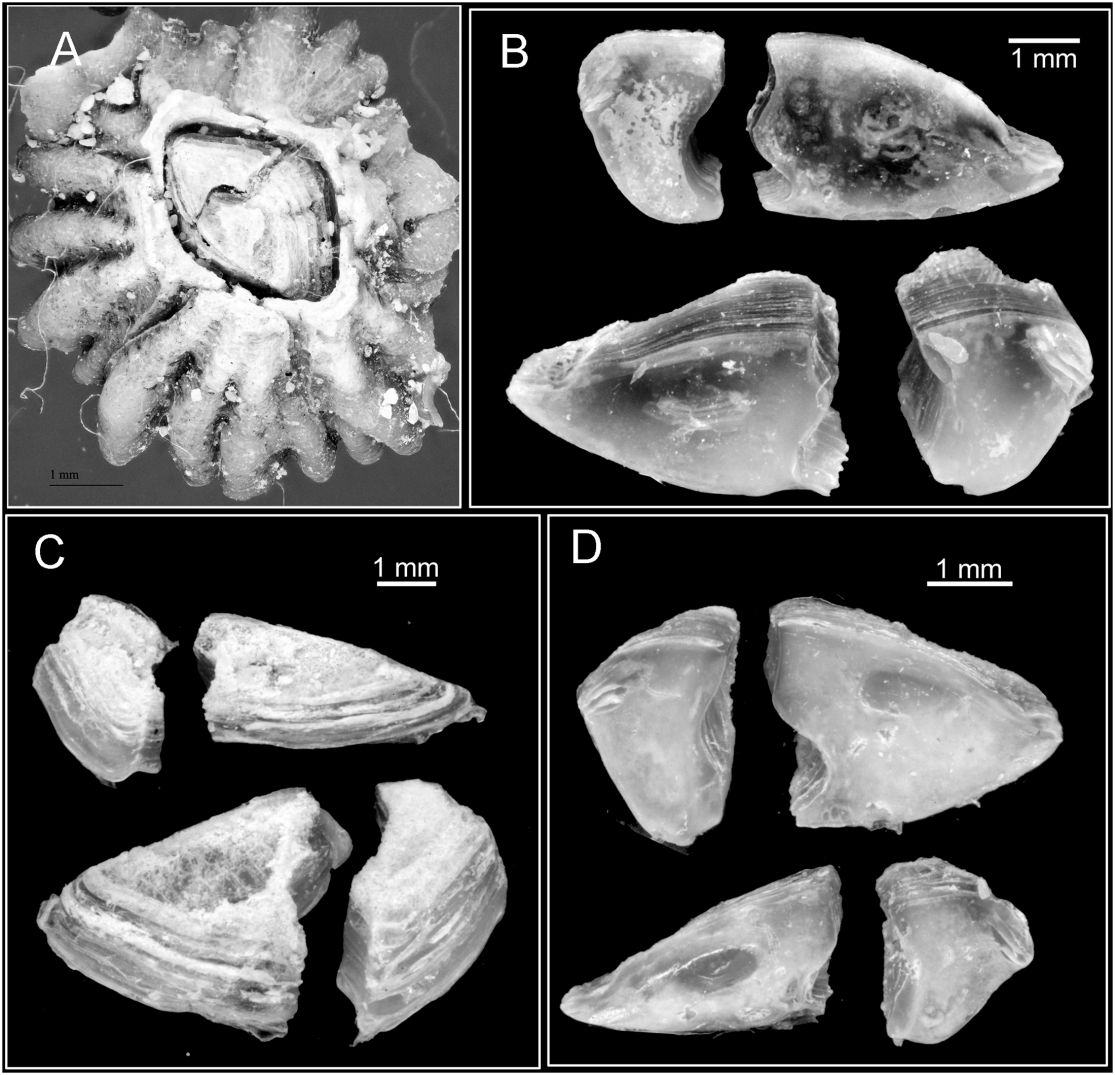
*Chthamalus anisopoma*, Point Lobos,Todos Santos, Baja California Sur, Mexico. A, whole view showing the orifice filled by the left scutum and tergum. B, inner view of scutum and tergum, showing the markedly assymetrical plates, with larger left scutum and tergum. C, outer view of paired scutum and tergum. D, inner view of scutum and tergum, showing a larger right scutum and tergum.

**Fig 7:**
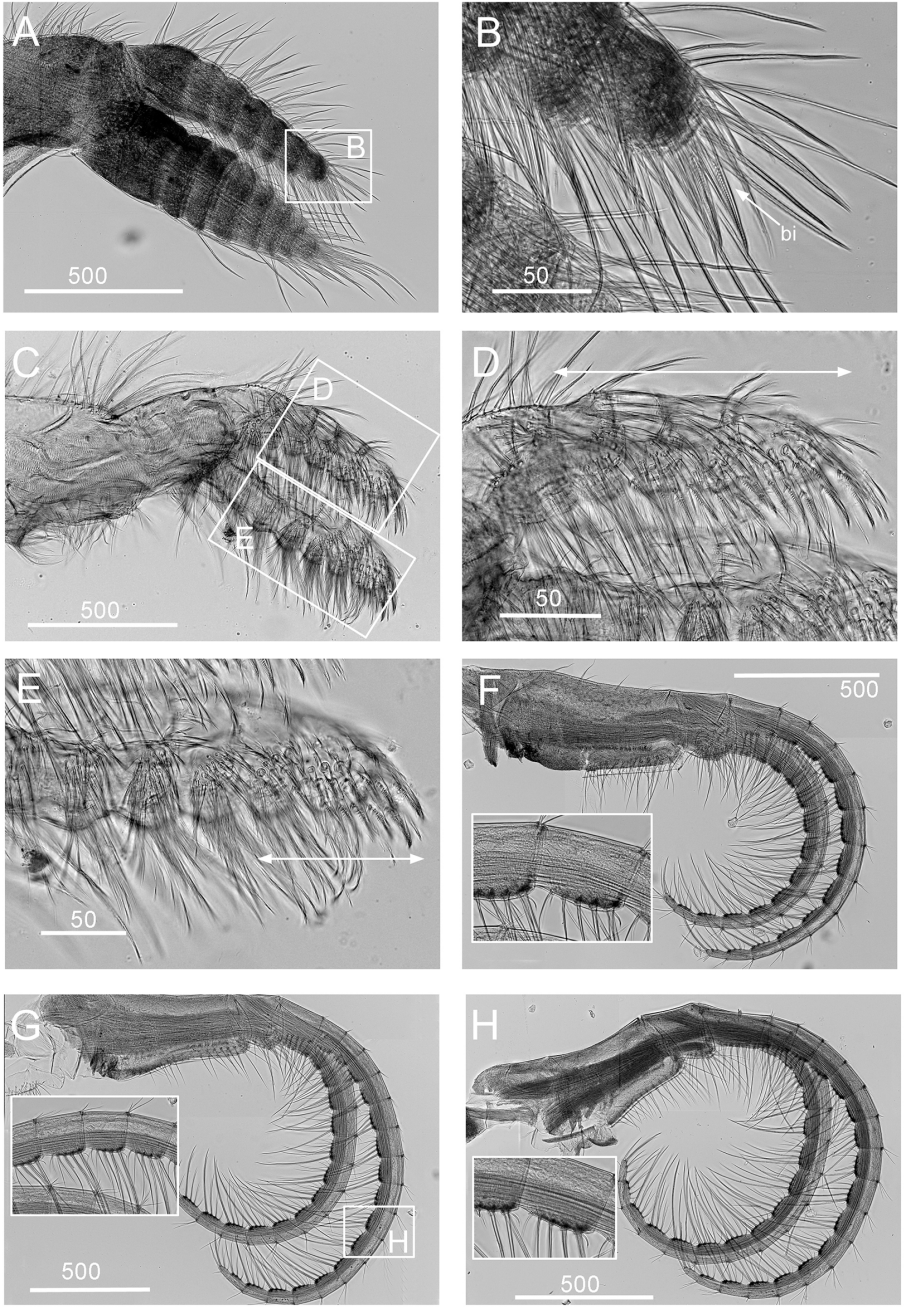
*Chthamalus anisopoma*, La Paz Bay, Baja California. A, B, cirrus I, showing the presence of bidenticulate setae (bi) without basal guards enlarged in B. C, D. E, cirrus II, showing the presence of bidenticulate setae with basal guards on the first few segments (indicated by double end arrows) in anterior ramus (D) and posterior ramus (E). F, cirrus III. G, cirrus IV. H, cirrus V. Intermediate segment of anterior rami of cirri III-V are shown as inserts in E-H. Scale bars in μm.

**Fig 8:**
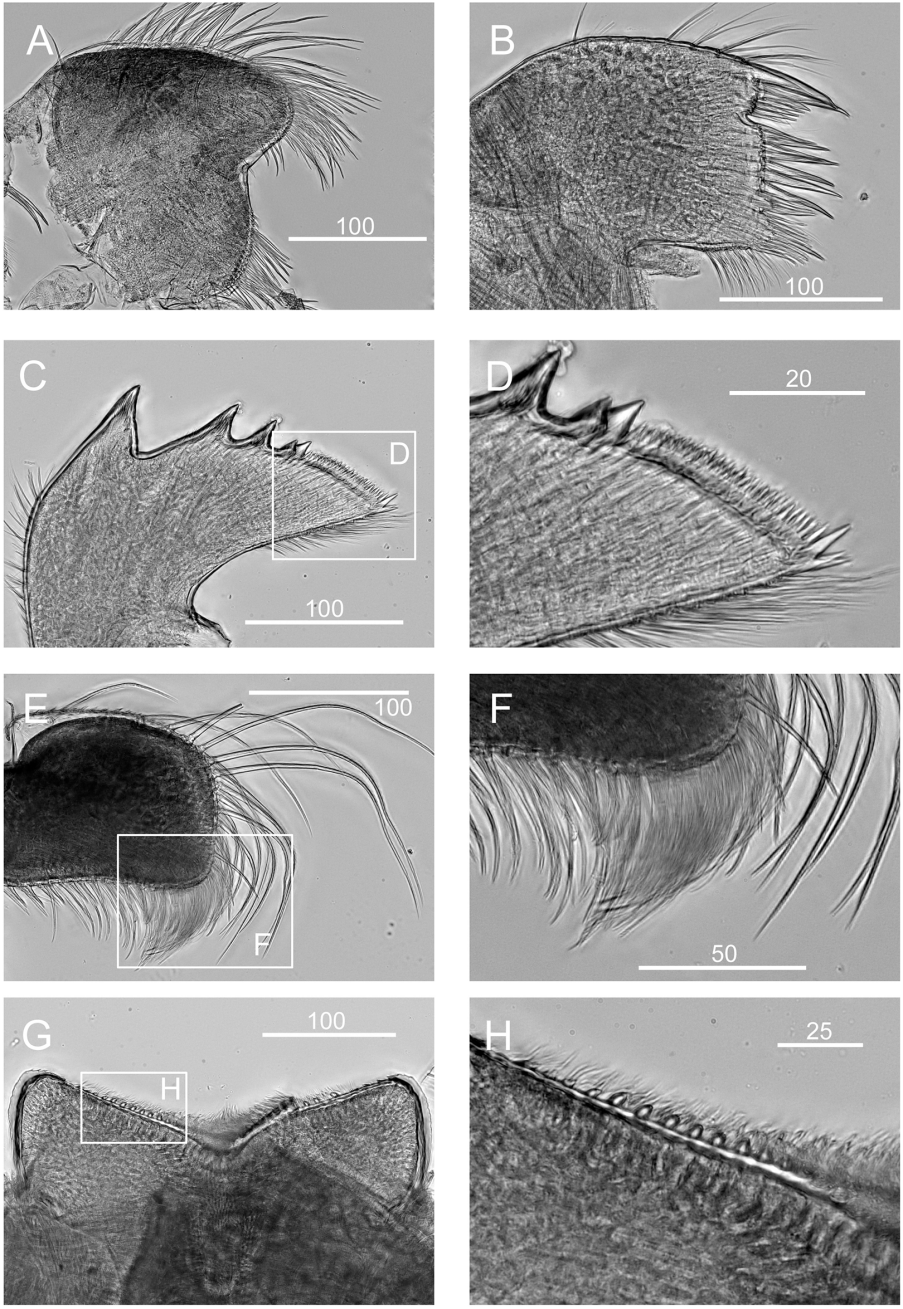
*Chthamalus anisopoma*, La Paz Bay, Baja California. A, maxilla. B, maxillule. C, mandible. D, third and fourth teeth, pecten and inferior angle of mandible. E, mandibular palp. F, serrulate setae on superior margin of mandibular palp. G, labrum. H, cutting margin of labrum showing the fine teeth. Scale bars in μm.

*Chthamalus anisopoma* Pilsbry, 1916: 317, pl 74, fig 2–2f. [2]—Barnes & Barnes, 1965: 392. [26]—Henry, 1942: 127. [27]—Henry, 1943: 372. [28]—Henry, 1960: 144 [29]—Kolosvary, 1941: 70. [30]—Nilsson-Cantell, 1921: 276. [31]—Pitombo & Burton, 2007: 14. [12]

#### Specimens examined

AJS-2, 36 specimens, intertidal shores, La Paz Bay, Baja California, 24° 13' N, 110° 18.6' W, 3 Nov 1978; AJS-3, 6 specimens, intertidal shores, Point Lobos,Todos Santos, Baja California Sur, Mexico, 23° 24.8' N, 110° 57' W, 4 Nov 1978.

TEPE78-24, 12 specimens, intertidal rocks, Isla Cerralvo, Baja California Sur, Mexico, 24° 08' N, 109° 48' W, 14 April, 1978; TEPE78-35, 36 specimens, La Paz Promenade, Baja California Sur, Mexico, 24° 09' N, 110° 15' W, 18 April, 1978.

#### Diagnosis

*Chthamalus* with strongly asymmetrical scuta and terga. Orifice often filled by left or right tergum and scutum. Cirrus I distalmost segment of posterior ramus with 2–3 bidenticulate setae. Cirrus II with bidenticulate setae with basal guards on the 4 distalmost segments of the posterior ramus and with bidenticulate setae with basal guards on the two distal segments of the anterior ramus. White tergoscutal flaps in fresh specimens.

#### Description

Shell white, depressed conic, external surface strongly and irregularly ribbed (Figs 2A and 6A). Eroded specimens often have the basal region of the shell ribbe d but the apical region is smooth. Orifice elliptical and often closed by the left tergum and scutum (Figs 2A and 6A). Tergoscutal flaps have white edge (Fig 2A) (the white colour is still visible after preservation in formalin or ethanol). Scutum and tergum strongly asymetrical (Fig 6B–6D). Individuals can have the left scutum and tergum larger than the right scutum and tergum or vice versa (Fig 6B and 6D). The larger scutum is triangular, with equal sides, the smaller one is narrower. The external surface of the scutum is smooth with growth lines near the basal margin (Fig 6C), occludent margin smooth, tergal margin with shallow articular ridge, not extending beyond the tergal margin. Adductor muscle scar oval, lateral depressor muscle scar oval and smooth. Tergum triangular to trapezoid, 3–4 lateral depressor muscle crests on basal margin (Fig 6B and 6D)

Segment counts on cirri I-III were based on five specimens collected from La Paz Bay, Baja California (AJS-2). Segment counts on cirri IV-VI were based on a single specimen from La Paz Bay, Baja California (AJS-2) (Table 4). Cirrus I: posterior ramus with 6 or 7 segments, anterior ramus 7- or 8-segmented, both rami with serrulate setae dominant, the distalmost segment of the posterior ramus with 2–3 bidenticulate setae (Fig 7A and 7B, Table 4). Cirrus II: posterior ramus 5-segmented, anterior ramus 6- or 7-segmented, serrulate type setae dominant on both rami. Posterior ramus with bidenticulate setae with basal guards on the 4 distalmost segments (Fig 7C and 7D, Table 4), anterior ramus with bidenticulate setae with basal guards on the two distalmost segments (Fig 7C and 7E, Table 4). Cirri III-VI: similar in morphology, serrulate type setae dominant, anterior ramus similar in length to the posterior ramus. Cirrus III: anterior ramus 13 to 19-segmented, posterior ramus 11 to 16- segmented (Fig 7F, Table 4). Cirrus IV: anterior ramus 17-segmented, posterior ramus 15-segmented (Fig 7G). Cirrus V and VI: anterior ramus 18-segmented, posterior ramus 17-segmented (Fig 7H). Intermediate segments of anterior rami of cirri IV-VI have 4 pairs of long serrulate setae and 1 pair of short serrulate setae (Fig 7F–7H).

Maxilla bilobed, with serrulate setae on both lobes (Fig 8A). Maxillule notched, 2 large and 4 medium sized setae above notch, 16 setae below notch of cutting margin (Fig 8B). Mandible has 4 major teeth, first tooth separated from the remainder, fourth tooth bi-dentate. Pecten long and straight, with 30 fine teeth, inferior angle tipped with two pointed teeth (Fig 8C and 8D). Mandibular palp rectangular, with serrulate setae on all margins (Fig 8E and 8F). Labrum V-shaped, with ~ 10 small teeth on both sides of the cutting margin (Fig 8G). The middle notch with dense setae (Fig 8H).

Penis without basi-dorsal point.

#### Distribution

Present in the Gulf of California and on the Pacific coast of Baja California Sur to at least 23° 27' N.

Remarks. *C*. *anisopoma* is sympatric with *C*. *alani* nom. nov. and *C*. *hedgecocki* on some shores. *C*. *anisopoma* can be easily identified *in-situ* by its strongly asymmetrical opercular plates, also by its white tergoscutal flaps when open under water (Fig 2A).

### *Chthamalus hedgecocki* Pitombo & Burton, 2007

Figs 2C and 9–11

**Fig 9:**
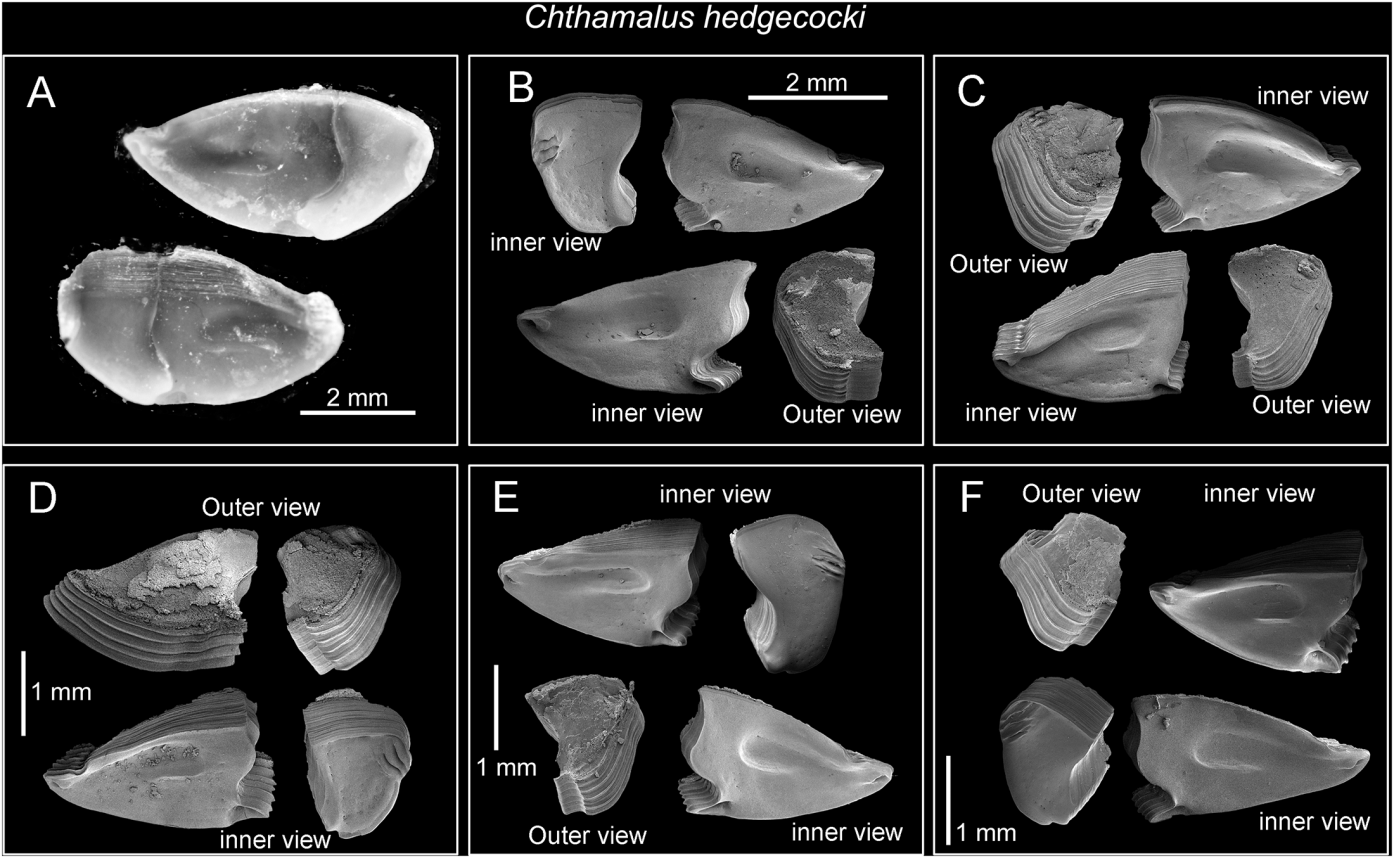
*Chthamalus hedgecocki*, Point Lobos,Todos Santos, Baja California Sur, Mexico. A, light microgaph showing paired articulated scutum and tergum. Note the scutum and tergum are articulated transversely. B-F, SEM photographs. B, C, E, F, Inner views of paired scutum, outer and inner view of tergum. D. Inner and outer view of paired scutum and tergum. Note the absence of longitudinal furrow on outer surface of tergum and the slightly asymmetry of paired scutum and tergum.

**Fig 10:**
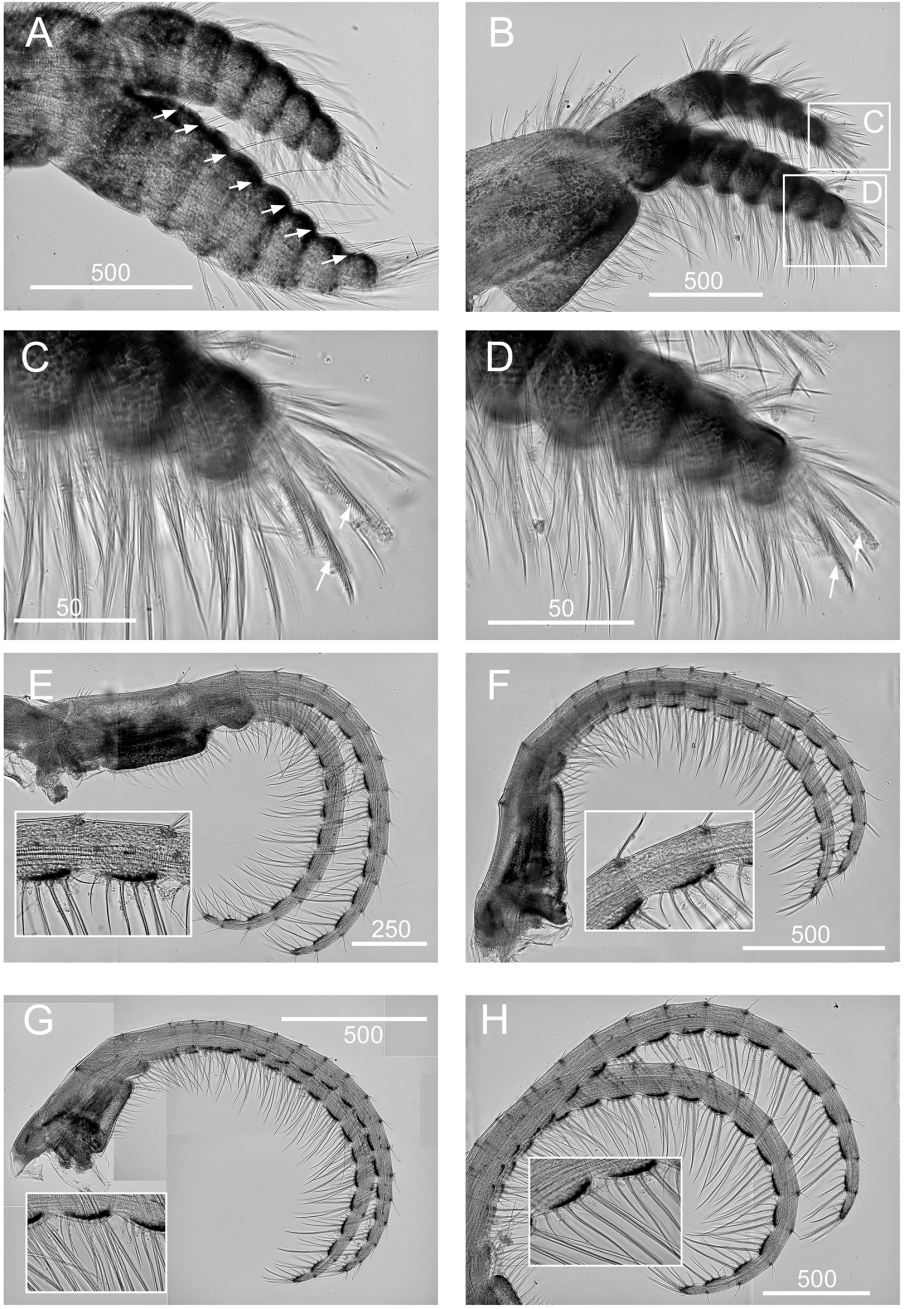
*Chthamalus hedgecocki*, Point Lobos,Todos Santos, Baja California Sur, Mexico. A, cirrus I, white arrows show a longitudinal array of setae on outer surface of posterior ramus. B, C, D, Cirrus II, showing the presence of bidenticulate setae with basal guards on the first few segments (indicated by double end arrows) on: C, anterior ramus; D, posterior ramus. E, cirrus III. F, cirrus IV. G, cirrus V. H, Cirrus VI. Intermediate segments of anterior rami of cirri III-VI are shown as inserts in E-H. Scale bars in μm.

**Fig 11:**
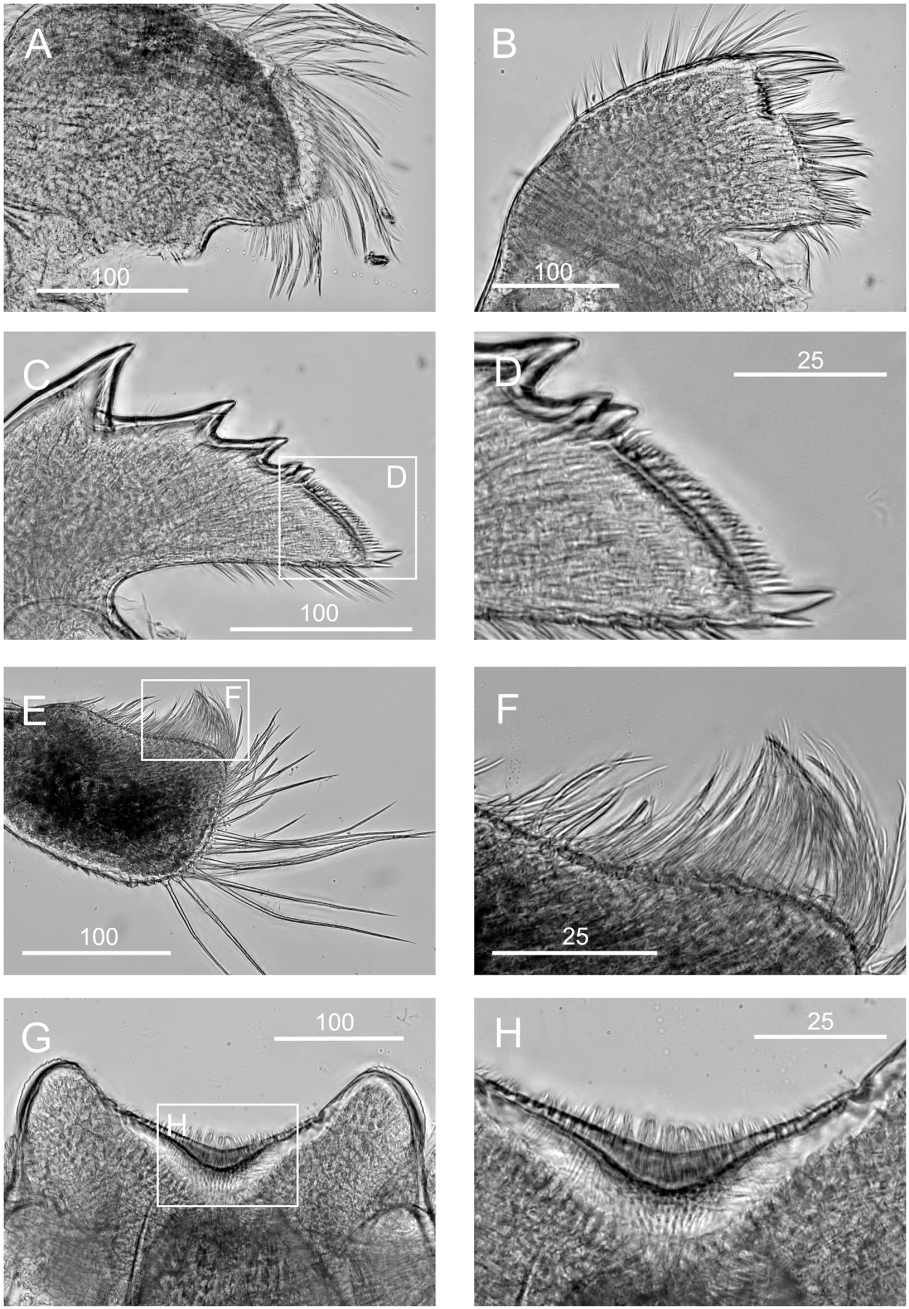
*Chthamalus hedgecocki*, Point Lobos,Todos Santos, Baja California Sur, Mexico. A, maxilla. B, maxillule. C, mandible. D, third and fourth teeth, pecten and inferior angle of mandible. E, mandibular palp. F, serrulate setae on superior margin of mandibular palp. G, labrum. H, cutting margin of labrum showing the fine teeth. Scale bars in μm.

BC *Chthamalus fissus* Hedgecock, 1979: 210, Table 2. [5]

*Chthamalus hedgecocki* Pitombo & Burton, 2007: 4, figs 2–7. [12]—Wares *et al*., 2009: Table 2 [14]

#### Specimens examined

AJS-3, 15 specimens, intertidal shores, Point Lobos,Todos Santos, Baja California Sur, Mexico, 23° 24.8' N, 110° 57' W, 4 Nov 1978; TEPE78-14, 10 specimens, exposed rocks and sheltered boulders, Isla Sacrificios, Oaxaca, Mexico, 15° 40.8' N, 96°14.6' W; TEPE78-35, 30 specimens, La Paz, Promenade, Baja California Sur, Mexico, 24° 09' N, 110° 15' W, 18 April, 1978.

Diagnosis. *Chthamalus* with shell pink-purple to brown externally (white in older and eroded specimens). Outer side of cirrus I with an array of long serrulate setae (one on each segment) on the anterior ramus. Posterior ramus of cirrus I lacks bidenticulate setae. Cirrus II has bidenticulate setae with basal guards on one or two of the distalmost segments of the posterior ramus and the distalmost segment of the anterior ramus. Tergoscutal flaps dull orange-brown in colour, when alive. COI sequence (Gene Bank Numbers: KU356704—KU356705).

#### Description

External shell surface pink-purple to white in eroded or bleached specimens, surface ribbed but ribs do not extend to apex (Fig 2C). Inside of shell pale brown to white. Orifice elliptical. Scutum and tergum slightly asymmetrical, scutum and tergum articulated transversely (Fig 9A). In older specimens the outer surface of the scutum and tergum was eroded, horizontal growth lines visible on the basal region (Fig 9B–9F). Scutum triangular, tergal margin about half the length of the basal margin, articular ridge not, or seldom, extending beyond the tergal margin, articular furrow deep. Occludent margin without teeth, smooth. Basal margin slightly convex, with obvious lateral depressor muscle crest (Fig 9). Tergum trapezoid, scutal margin slightly convex, spur wide and blunt, carinal margin with 4–5 lateral depressor muscle crests (Fig 9B and 9E).

Segment counts on cirri I-III were based on five specimens collected from Point Lobos, Baja California Sur, Mexico (AJS-3). Segment counts on cirri IV-VI were based on a single specimen from Point Lobos, Baja California Sur, Mexico (AJS-3) (Table 4). Cirrus I: posterior ramus 5–7 segmented, anterior ramus 7- to 9-segmented. Outer side of anterior ramus with a longitudinal array of long, serrulate setae, a single seta on each segment, posterior ramus setae serrulate, no bidenticulate setae (absent from all specimens examined, Fig 10A). Cirrus II: posterior ramus 5- to 6-segmented, anterior ramus 5- to 6-segmented, first or first and second most distal segments of the posterior and the most distal segment of the anterior ramus with bidenticulate setae with basal guards (Fig 10B and 10D, Table 4). Cirrus III: anterior and posterior rami similar in length, anterior ramus 13- to 19-segmented, posterior ramus 14- to 17-segmented, intermediate segments have 3 pairs of long setae and 2 pairs of short simple setae (Fig 10E, Table 4). Cirrus IV and V: anterior and posterior rami 16-segmented, intermediate segments of both rami carry 3 pairs of long serrulate setae and 2 pairs of short simple setae (Fig 10F and 10G). Cirrus VI: anterior and posterior rami 19-segmented (Fig 10H).

Maxilla with serrulate setae (Fig 11A). Maxillule notched, two large setae above notch, notch wide and shallow, with 4–5 setae in notch, region below notch protuberant, with > 14 setae along the protuberant cutting edge (Fig 11B). Mandible has 4 major teeth, fourth tooth bi-dentate (Fig 11C). Pecten long, with 24 very fine teeth, inferior angle with a pair of pointed teeth (Fig 11D). Mandibular palp rectangular, with serrulate setae on superior margin and tip (Fig 11E and 11F). Labrum with V-shaped cutting edge, without sharp teeth on either side of the cutting margin, central part of the notch carries dense setae (Fig 11G and 11H).

Penis without basi-dorsal point.

#### Distribution

Southward from 23° 39' N inside the Gulf of California, on the east coast, and 32° 52' N on the western outer coast of the Baja California Peninsula. The southern limit is north of 15° N on the Mexican Pacific coast. The type material of *Chthamalus hedgecocki* Pitombo & Burton, 2007 came from Punta Camarón, Mazatlan, Sinaloa, Mexico (23° 14' 15'' N, 106° 26' 45'' W) attached to rocks on a wave exposed shore, upper midlittoral zone. The geographical distribution was stated by Pitombo and Burton to extend from Baja California to Acapulco in southern Mexico (16° 50' 19'' N).

#### Remarks

Pitombo & Burton [12] reported that the outer surface of the tergum in *C*. *hedgecocki* posesses a weak longitudinal furrow, frequently only on one plate. In the present study of specimens from Point Lobos, Baja California, the tergum of *C*. *hedgecocki* is smooth externally and without any longitudinal furrows. The arthropodal characters of *C*. *hedgecocki* in the present study agree with the descriptions in Pitombo & Burton [12]. The shallow longitudinal furrow in at least one of the the terga of *C*. *hedgecocki* may be a variable character and may not be a diagnostic character for species identification.

### *Chthamalus newmani* sp. nov.

urn:lsid:zoobank.org:act:37C27151-25BE-4F94-AABD-6F06FEF8C7B2

**Fig 12:**
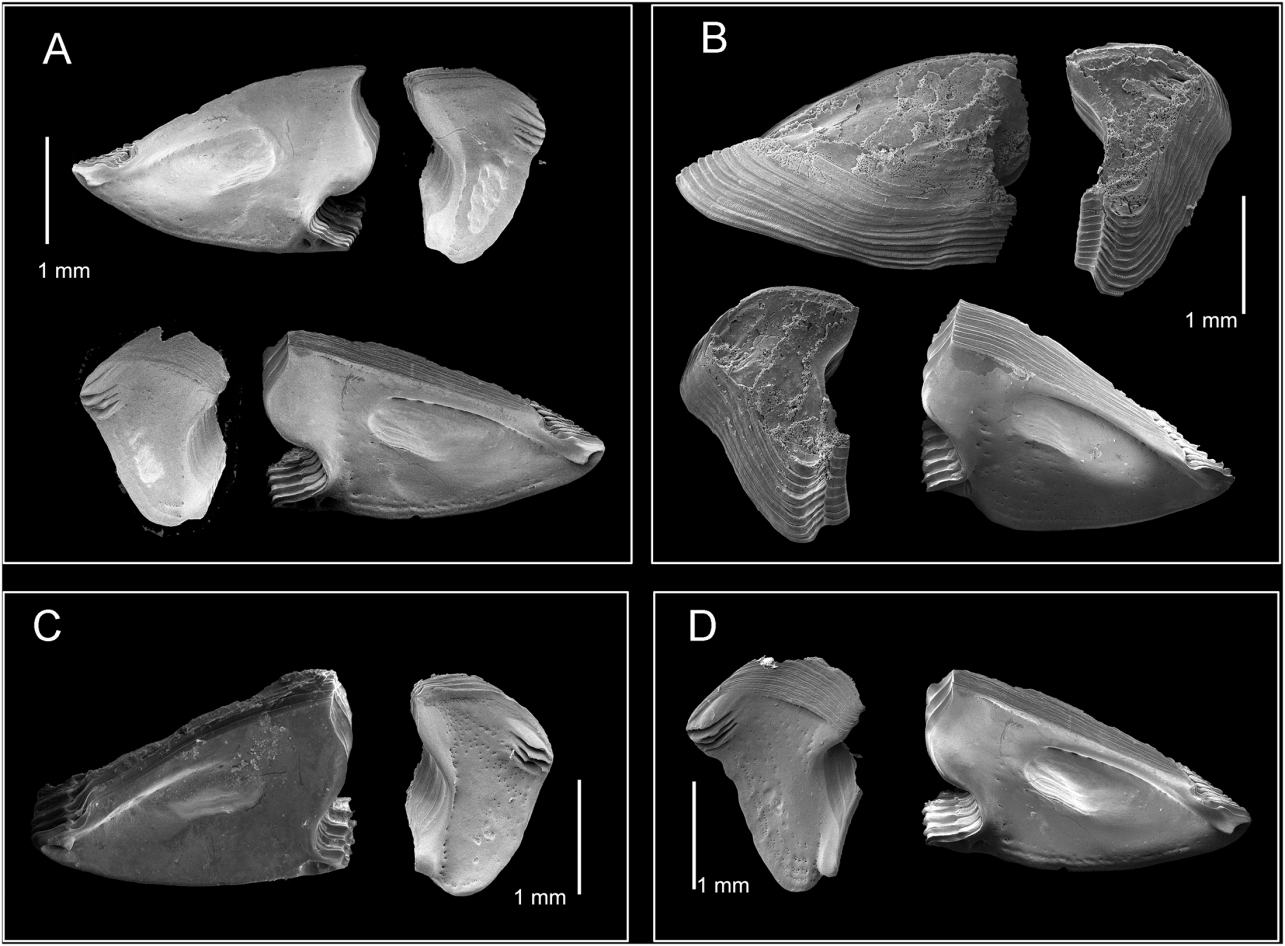
*Chthamalus newmani* sp. nov. SEM showing scutum and tergum. A, holotype (NHMUK 2016. 9), Panama City, inner view of paired scutum and tergum. B, additional specimens (TEPE78-1), Farfan Point. Inner view and outer view of scutum and outer view of tergum. C, D, paired scutum and tergum from additional specimens (TEPE78-1), Farfan Point. Note the variation in the shape of the scutum and tergum among samples.

**Fig 13:**
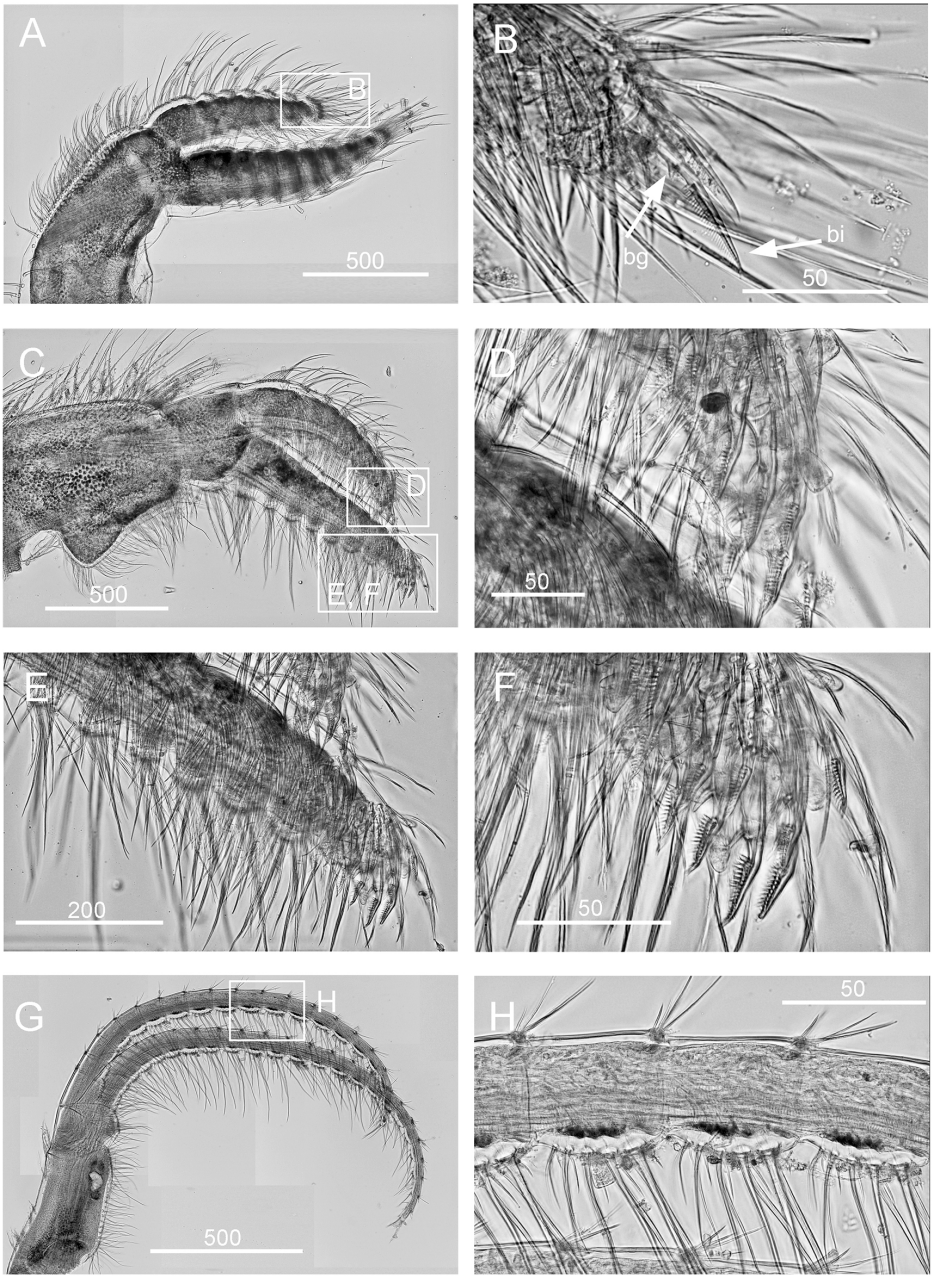
*Chthamalus newmani* sp. nov. Holotype (NHMUK 2016. 9), Panama City. A, B, cirrus I, showing the presence of bidenticulate setae (bi) with or without basal guards (bg), enlarged in B, C, D, E. F, cirrus II, showing the presence of bidenticulate setae with basal guards on the first few segments (indicated by double end arrows) of the anterior ramus (D) and the posterior ramus (E) and magnified view of bidenticulate setae with basal guards on the tip of the posterior ramus (F). G, cirrus III. H, intermediate segment of anterior ramus of cirrus III. Scale bars in μm.

**Fig 14:**
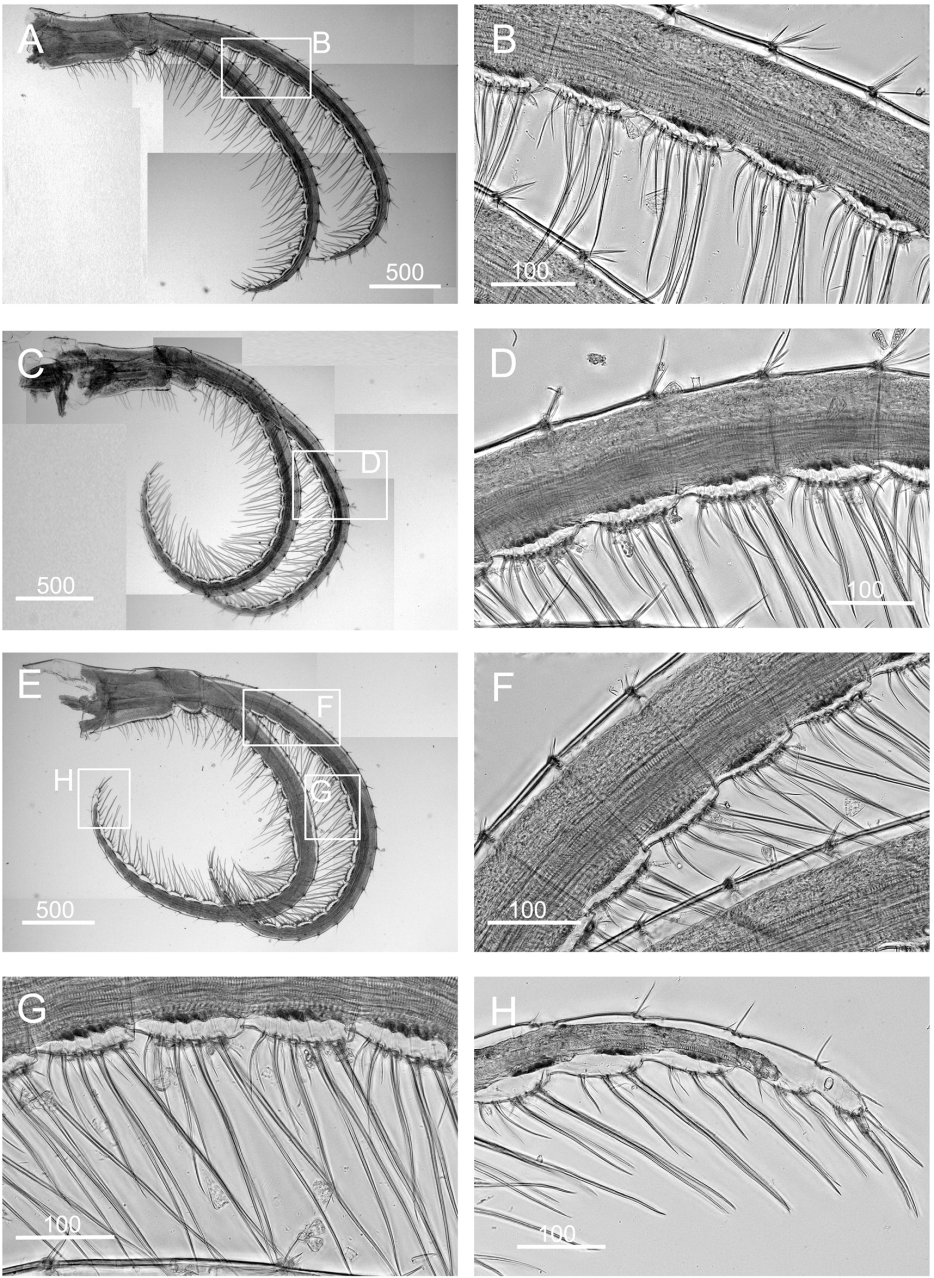
*Chthamalus newmani* sp. nov. Holotype (NHMUK 2016. 9), Panama City. A, B, cirrus IV. B, enlarged intermediate segment of anterior ramus of cirrus IV. C, D, cirrus V. D, enlarged intermediate segment of anterior ramus of cirrus V. E, F, cirrus VI F, enlarged intermediate segment of anterior ramus of cirrus VI. G, serrulate setae on basipodite of cirrus VI, H, setae on tip of anterior ramus of cirrus VI. Scale bars in μm.

**Fig 15:**
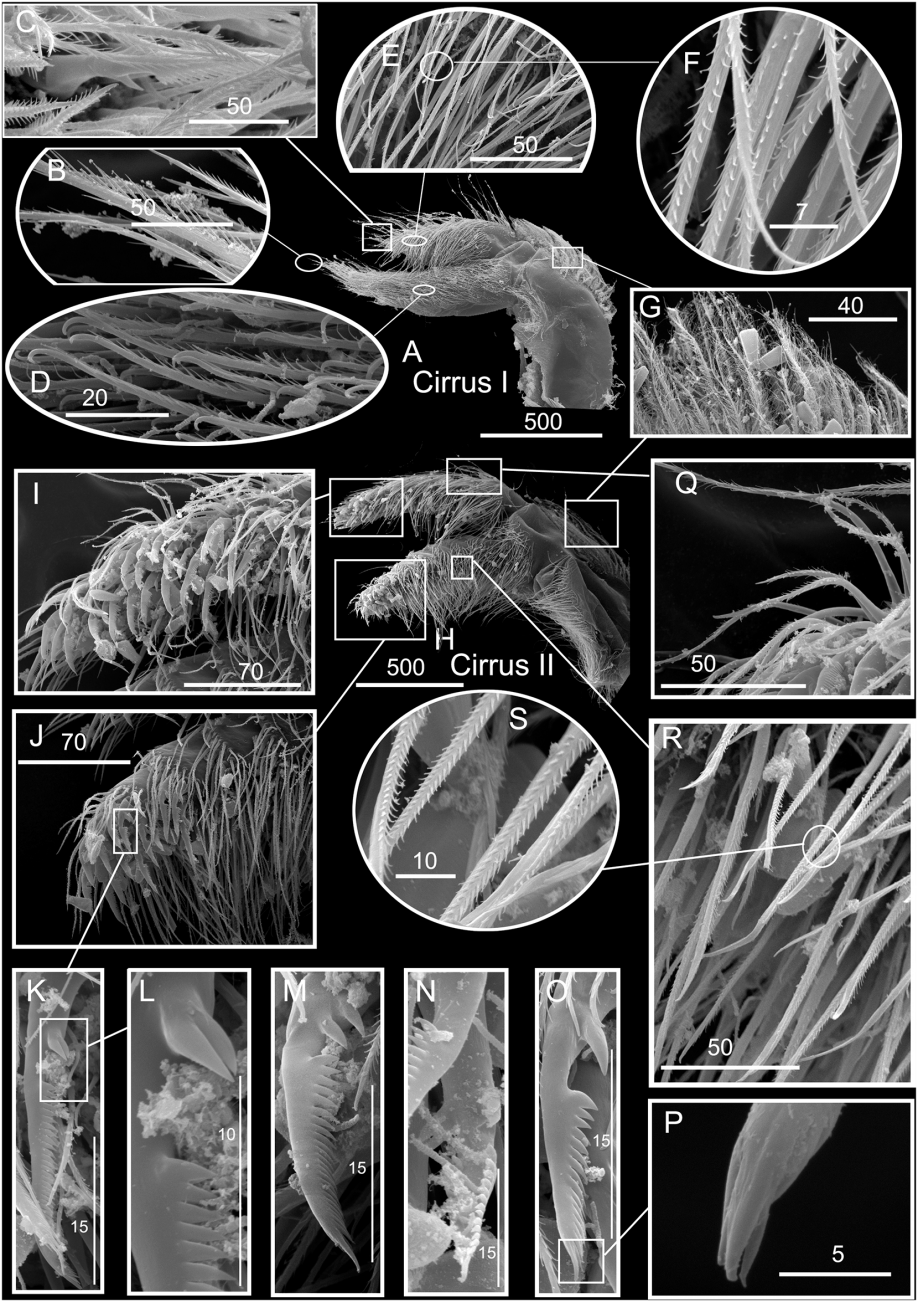
*Chthamalus newmani* sp. nov. Paratype (ASIZCR-000341), Panama City. SEMs. A–G, cirrus I. B, serrulate setae. C, bidenticulate setae on anterior ramus. D, F, serrulate setae on anterior and posterior rami. G, pappose setae on basipodite. H- Q, cirrus II. Bidenticulate setae with basal guards on anterior (I) and posterior (J) rami. Variation in the structure of bidenticulate setae with basal guards (note the number of basal guards) (K, P). serrulate setae (Q, R, S). Scale bars in μm.

**Fig 16:**
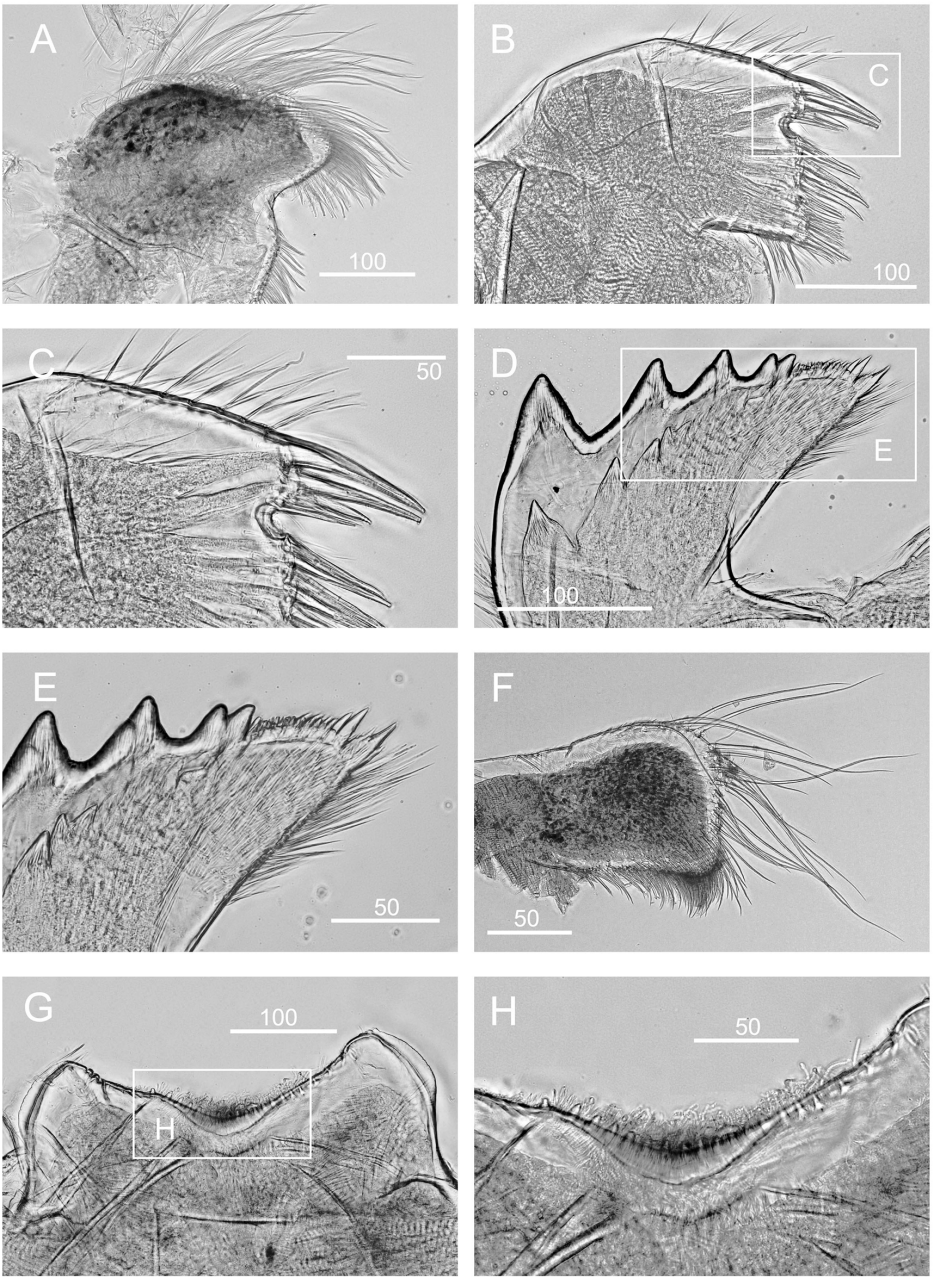
*Chthamalus newmani* sp. nov. Holotype (NHMUK 2016. 9), Panama City. A, maxilla. B, maxillule. C, notch region of maxillule. D, mandible. E, second to fourth teeth, pecten and inferior angle of mandible. F, mandibular palp. G, labrum. H, cutting margin of labrum showing the fine teeth. Scale bars in μm.

**Fig 17:**
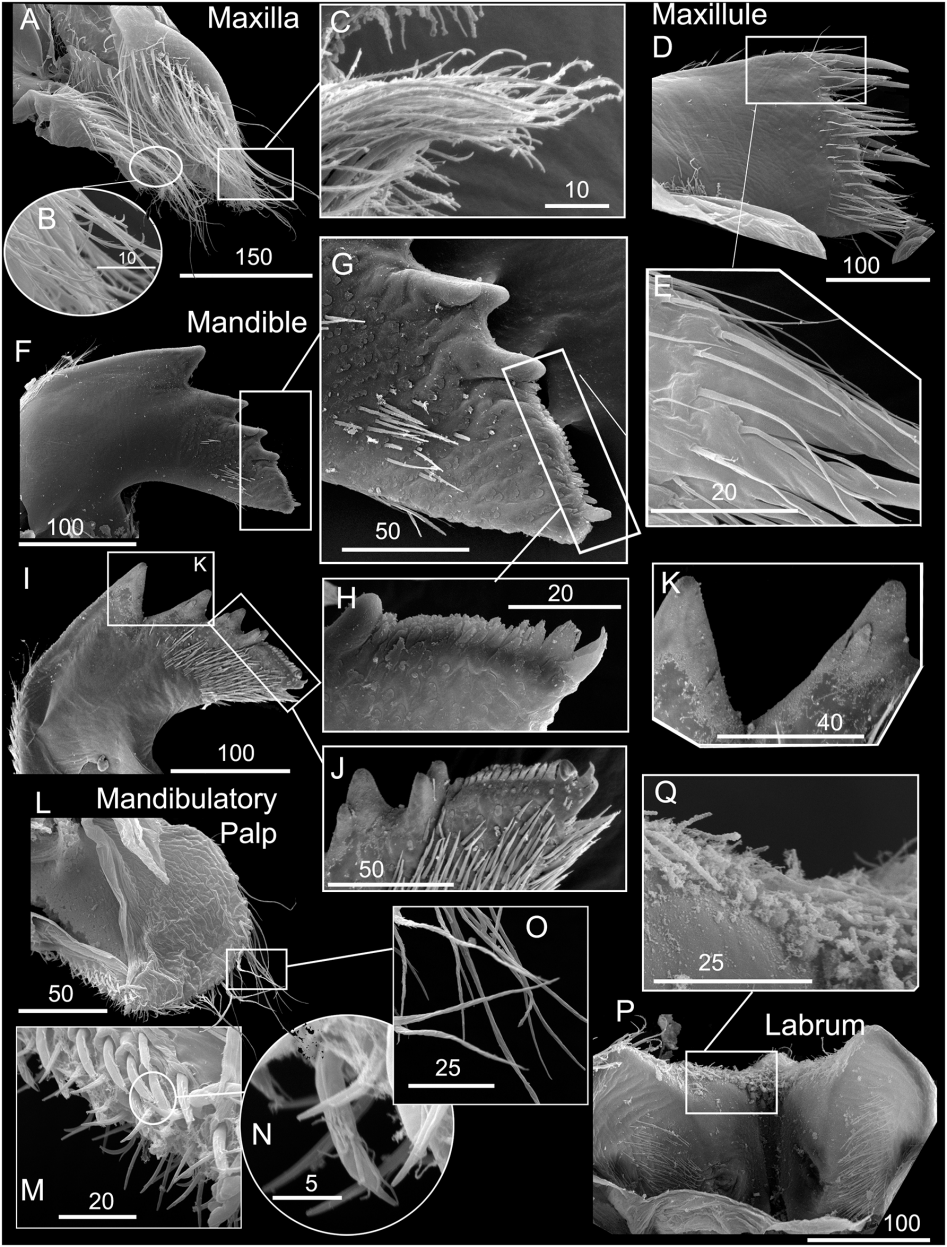
*Chthamalus newmani* sp. nov. Paratype (ASIZCR-000341), Panama City. SEMs of mouth parts. A, B, C, maxilla, showing the serrulate type setae enlarged in B and C. D, E, maxillule showing simple setae on surface of maxillule, enlarged in (E), F, G, mandible showing pecten (G). I—K, mandible of additional specimen, details of the bidenticulate teeth (K) and pecten (J). L–N, mandibular palp with details of serrulate setae on the superior margin (M, N) and on the palp tip (N). P- Q, labrum, showing teeth on cutting margin enlarged (Q). Scale bars in μm.

Figs 2D, 2E and 2F and 12–17

FB *Chthamalus fissus*.—Hedgecock, 1979: 210, Table 3 [5]

*Chthamalus southwardorum* B (in Table, not in text).—Wares *et al*., 2009: Table 2. [14]

*Chthamalus southwardorum* “Farfan” form.—Meyers *et a l*., 2013: 77, fig 2. [11]

#### Specimens examined

holotype, NHMUK 2016. 9 (= AJS 1970) 1 specimen from rock ledges, Balboa Statue, Panama City, Panama, 1970; paratypes, NHMUK 2016.10, 2016.11, 2016.12, 2016.13, 2016.14. 5 specimens, same data as holotype. ASIZCR-000334, 000335, 000336, 000341. 4 specimens, same data as holotype; other materials, TEPE78-1, 20 specimens, intertidal rocks, Farfan Beach, Farfan Point, Panama, 8° 56.4' N, 79° 34.1' W, 30 Mar 1978. PWG1, 6 specimens, intertidal rock, 200 m N of Naos Island on the west side of Avenida Amador, 8° 54.63' N, 79° 31.74' W, 3 Feb 1979.

#### Diagnosis

*Chthamalus* with cirrus I having bidenticulate setae with or without basal guards on the distal segment of the posterior ramus and cirrus II having bidenticulate setae with basal guards on the distalmost two or three segments of the posterior ramus and two distalmost segments of the anterior ramus. External surface of tergum without tergal furrow. Tergoscutal flaps brown when alive. COI sequence (Gene Bank Numbers: KU356709—KU356722).

#### Description

The hard parts were described mainly based on the holotype, supplemented with information from paratypes. Shell depressed, white to pale brown, composed of six plates, radii absent. Orifice elliptical (Fig 2D, 2E and 2F). Inside of shell white to pale brown. Scutum and tergum symmetrical, with tergoscutal flaps brown in colour when alive (Fig 2D, 2E and 2F), dull brown when preserved in formalin or ethanol. Scutum triangular, outer surface of scutum with upper part eroded, with growth lines as striation patterns on the lower basal region. Inner side of scutum white, tergal margin with wide articular ridge and deep articular furrow. Basal margin slightly convex, lateral muscle scar present as 1 or 2 pits. Occludent margin straight without dentations, adductor muscle scar oval and deep (Fig 12). Tergum narrow, height of tergum about 1.5 times greater than the width, tergal margin about 0.6 of the length of the basal margin (Fig 12). External surface eroded with growth lines visible near the basal margin, inner side white and smooth, scutal margin strongly concave, carinal margin convex, basal margin with 4 rostral depressor muscle crests. Spur wide and bluntly convex (Fig 12).

Segment counts on cirri I-III were based on five specimens collected from rock ledges, Balboa Statue, Panama City, Panama (AJS-1970) (Table 4). Segment counts on cirri IV-VI were based on the holotype. Cirrus I: posterior ramus with 6 or 7 segments, anterior ramus with 8 or 9 segments (Fig 13A). Distalmost segment of posterior ramus with 1–2 bidenticulate setae with or without basal guards (Figs 13B, 15A and 15C). Serrulate setae dominate on both rami (Fig 15A–15F). Basipod of cirrus I with pappose setae (Fig 15G). Cirrus II: posterior ramus with 5 to 7 segments, anterior ramus with 7 or 8 segments (Fig 13C) (Table 4), posterior ramus with bidenticulate setae with basal guards on the three distalmost segments (Fig 13D), anterior ramus with bidenticulate setae with basal guards on the two distalmost segments (Fig 13E). The number of basal guards on the bidenticulate setae ranges from 1–2 on different specimens (Fig 15H–15P). Serrulate setae are dominant on both rami (Fig 15R–15Q). Basipod with pappose setae. Cirrus III: anterior ramus about equal to posterior ramus, posterior ramus 17 to 19 segments, anterior ramus 18 to 23 segments (Fig 13G, Table 4). Intermediate segments of cirrus III with 3 pairs of long setae and 2 pairs of short setae (Fig 13H). Cirrus IV: anterior and posterior rami similar in length, anterior ramus 23 segments, posterior ramus 20 segments (Fig 14A) intermediate segments of anterior ramus with 3 pairs of long serrulate setae and 2 pairs of short simple setae (Fig 14B). Cirrus V: anterior and posterior rami similar in length, anterior ramus 21 segments, posterior ramus 20 segments (Fig 14C), intermediate segments of both rami with 3 pairs of long serrulate setae and 2 pairs of short simple setae (Fig 14D). Cirrus VI: anterior and posterior rami similar in length, anterior ramus 25 segments, posterior ramus 26 segments (Fig 14E), intermediate segments of both anterior and posterior rami with 3 pairs of long serrulate and 2 pairs of short simple setae (Fig 14F). Basipodite (Fig 14G) and tip of cirrus VI with serrulate setae (Fig 14H).

Maxilla, two lobes are separated by a notch without setae, serrulate setae on both lobes (Figs 16A and 17A–17C). Maxillule cutting margin notched, with two large setae above notch, five setae below notch, inferior angle protuberant, with dense setae (Figs 16B, 17D and 17E). Mandible has 4 major teeth, first tooth separated from the remainder, the third and fourth teeth bi-dentate, pecten with 14 very small teeth, inferior angle tipped with a pair of pointed teeth (Figs 16D and 16E and 17G–17K). Mandibular palp rectangular, superior margin with dense serrulate setae, inferior margin with long serrulate setae (Figs 16F and 17L, 17M and 17N). Labrum concave, with six teeth on each side of the cutting margin, central part of notch with dense setae (Figs 16G and 16H and 17P and 17Q).

Penis without basi-dorsal point.

#### Distribution

Found on the Pacific coast of Central America from 13° 55' N, 90° 47' W to 8°51' N, 79° 44' W (range further south unknown).

Etymology. Named (by BKKC) in honour of Prof. William Newman, for his extensive contributions in barnacle taxonomy and his leadership of the TEP Expedition (see Southward [32]).

#### Remarks

Hedgecock [5] collected specimens which he designated “FB *Chthamalus fissus*” from Farfan Beach, Panama and found identical specimens at Isle del Tigre, Golfo de Fonseca. Using allozyme electrophoresis, he showed these to be a separate species from *C*. *fissus* of California and the species collected at Isla Sacrificios and Chamela Bay. Specimens from the Farfan Beach collection of Hedgecock were examined by electrophoresis and by morphology in the present study and found to be *C*. *newmani*.

### *Chthamalus panamensis* Pilsbry, 1916

**Fig 18:**
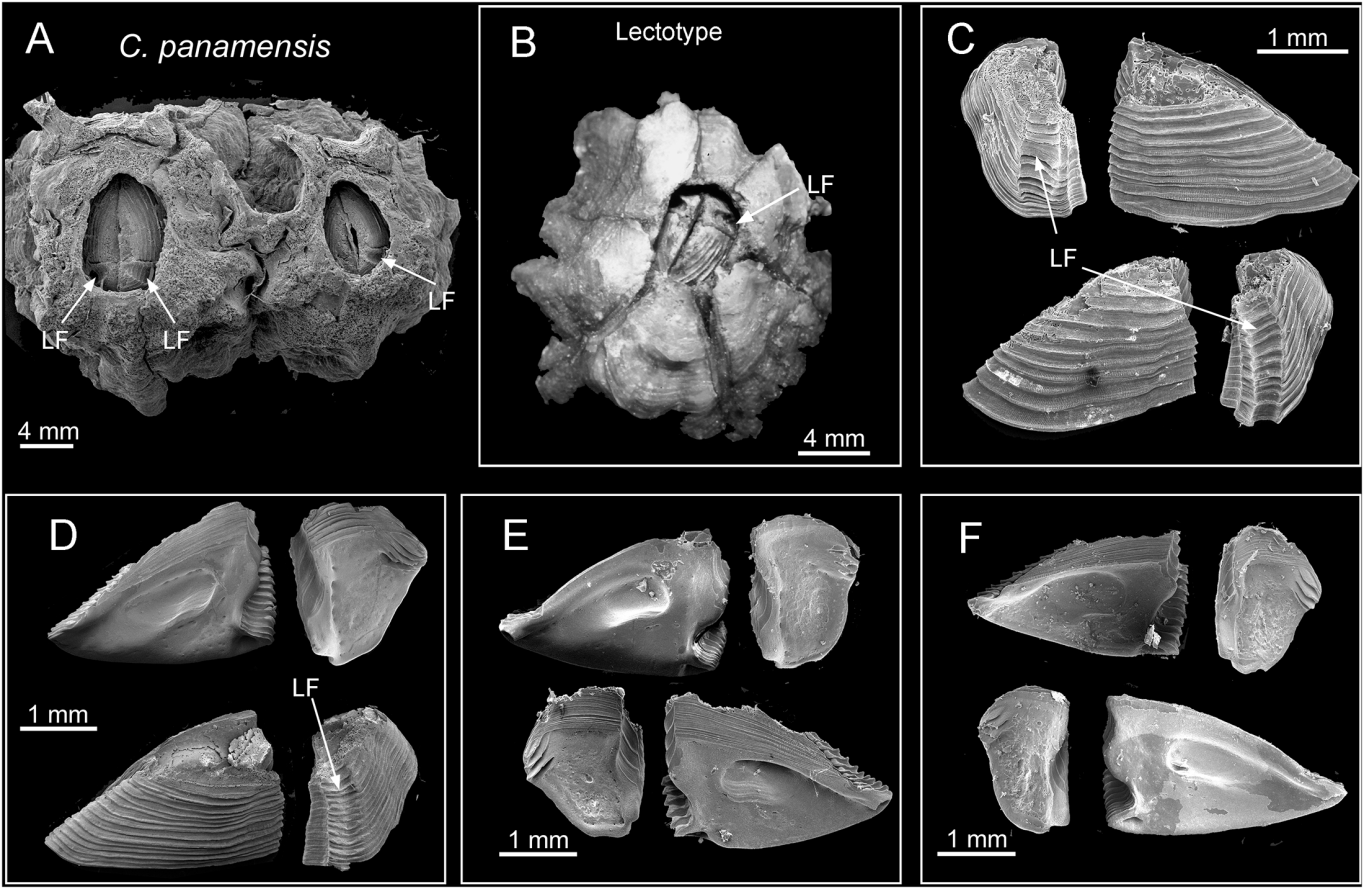
*Chthamalus panamensis* specimens. A. from Avenida Amador, Naos Island, Panama, Panama, showing the top view of shell and the appearance of the longitudinal furrow (LF) on tergum, through the orifice. B, The intact specimen illustrated in Pilsbry’s Plate 75, fig 2a is selected by the present authors as the Lectotype, showing the longitudinal furrow (LF) on the outer surface of the tergum. C-F, from Avenida Amador, Naos Island, Panama. SEMs of scutum and tergum from 4 specimens. Note the variation in the shape of the tergum among individuals. C, outer view of scutum and tergum, showing horizontal growth lines and longitudinal furrow on tergum (LF). D, scutum and tergum (inner and outer view) showing longitudinal furrow on tergum outer surface. E, F, inner side of scutum and tergum.

**Fig 19:**
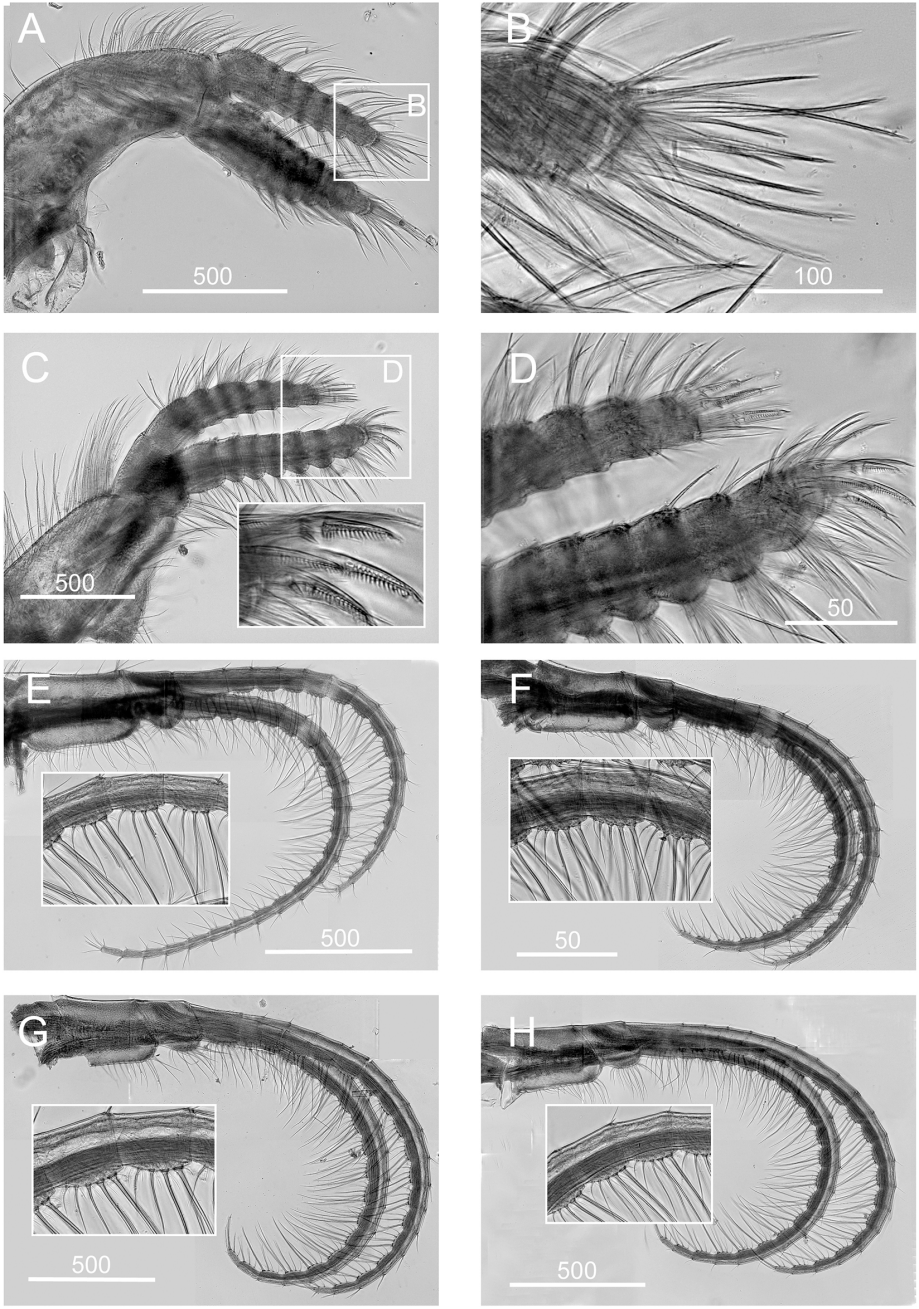
*Chthamalus panamensis* from Avenida Amador, Naos Island, Panama. Light micrographs. A, B, cirrus I. B, detail showing the absence of a bidenticulate seta on the anterior ramus. C, D, cirrus II. D, showing the bidenticulate setae with basal guards, in the insert, and the presence of bidenticulate setae with basal guards on the first segment of anterior and posterior rami. E, cirrus III: note the difference in lengths of the two rami. F, cirrus IV. G, cirrus V. H, cirrus VI. Intermediate segments of anterior rami of cirri III-VI are shown as inserts in E-H. Scale bars in μm.

**Fig 20:**
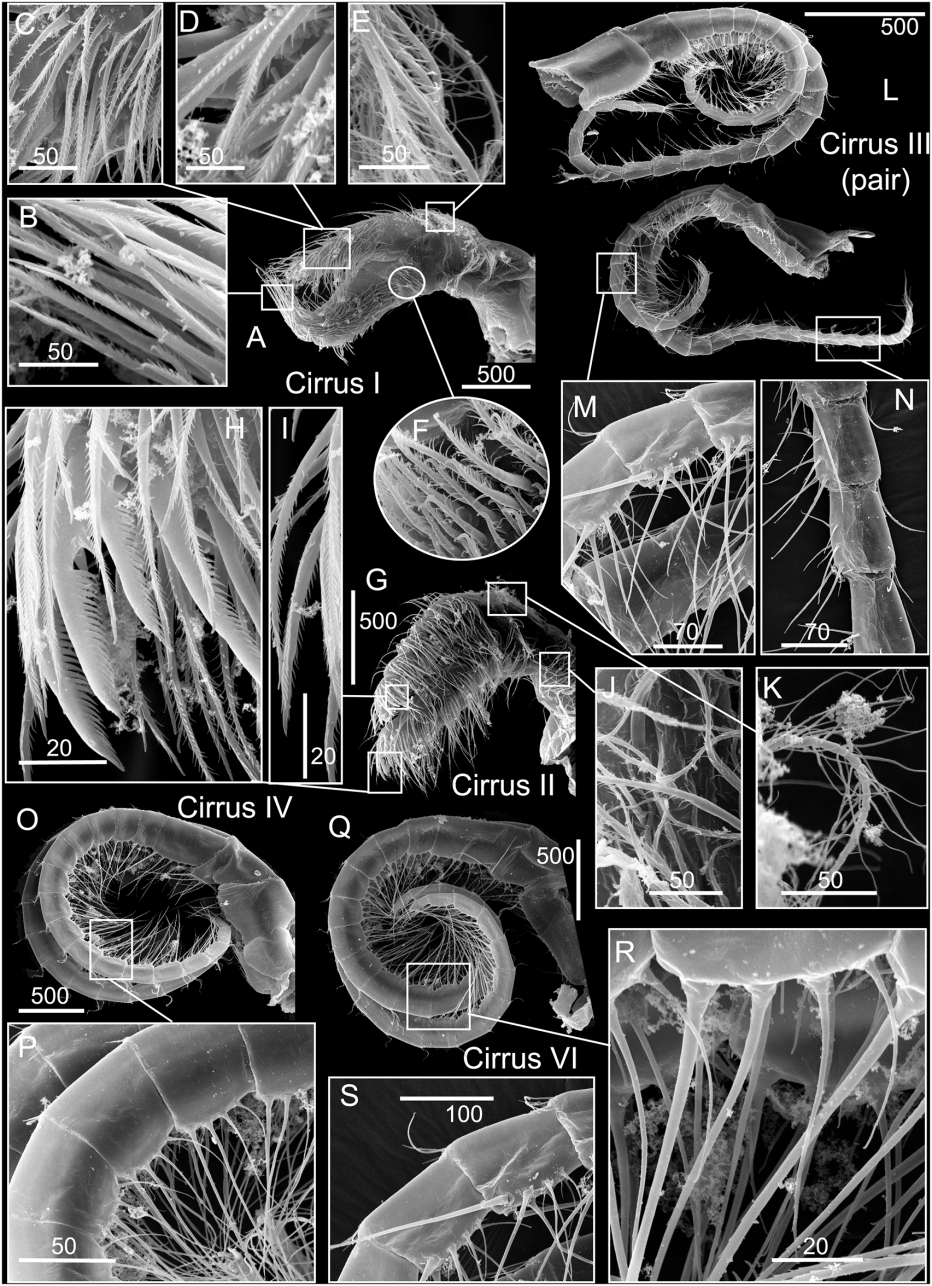
*Chthamalus panamensis* from Avenida Amador, Naos Island, Panama. SEMs. A-F, cirrus I, with details of serrulate setae in B, C, D, F on rami and pappose setae (E) on basipodite. G–K, cirrus II, showing details of: H, bidenticulate setae with basal guards; I, serrulate setae on first segments of anterior and posterior rami; J, K, pappose setae on basipodite. L, cirrus III (left and right pair), note the difference in length of anterior and posterior rami. M, intermediate segments of posterior ramus of cirrus III. N, intermediate segmenst of anterior ramus of cirrus III. O-P, cirrus IV, with detail of intermediate segment in P. Q-S, cirrus VI, with details of intermediate segments in (S) and (R). Scale bars in μm.

**Fig 21:**
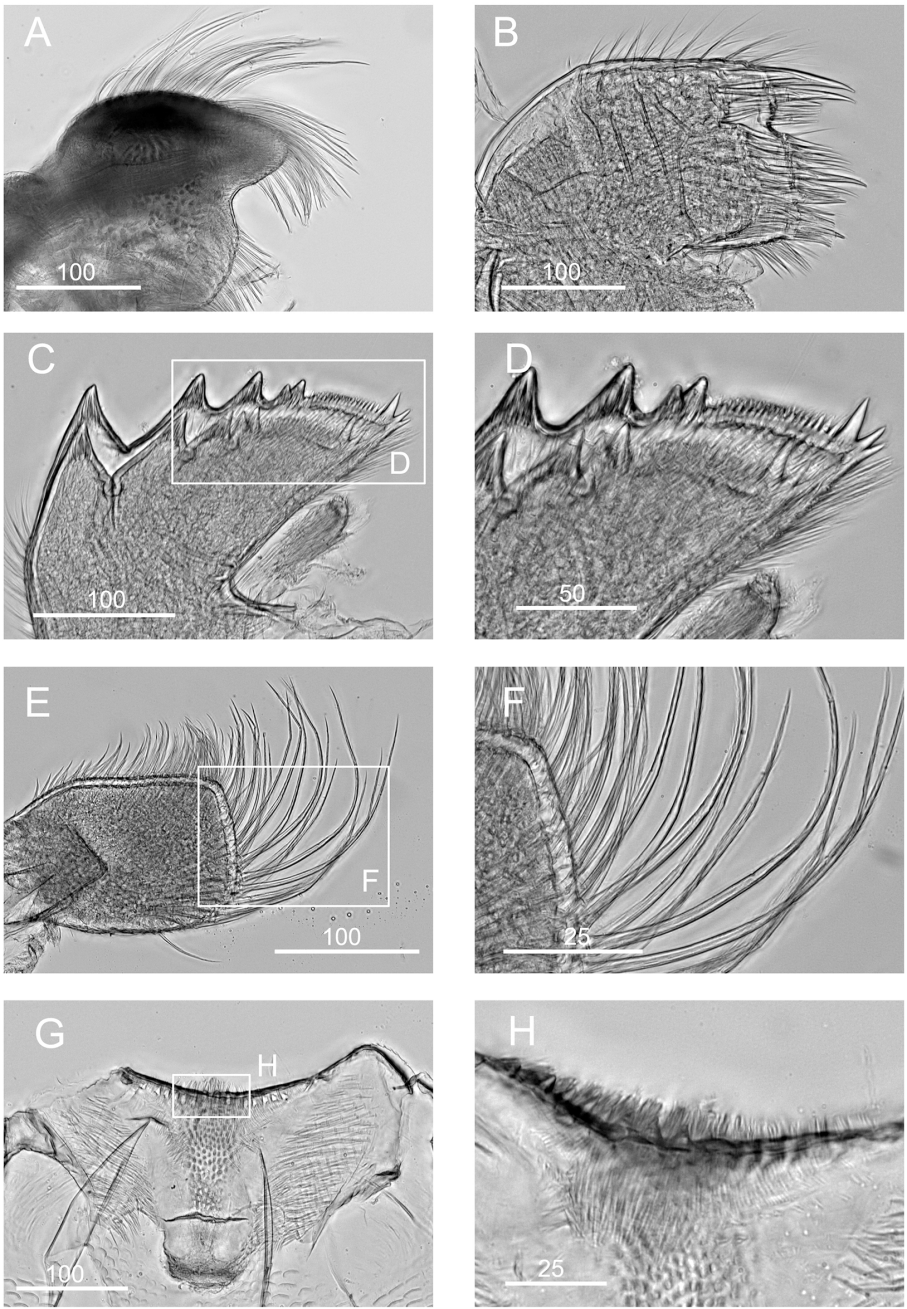
*Chthamalus panamensis* from Avenida Amador, Naos Island, Panama. Light Micrographs. A, maxilla. B, maxillule. C. mandible. D, second to fourth teeth, pecten and inferior angle of mandible. E- F, mandibular palp, with details ofserrulate setae on superior margin of mandibular palp (F). G, labrum. H, cutting margin of labrum showing the fine teeth. Scale bars in μm.

**Fig 22:**
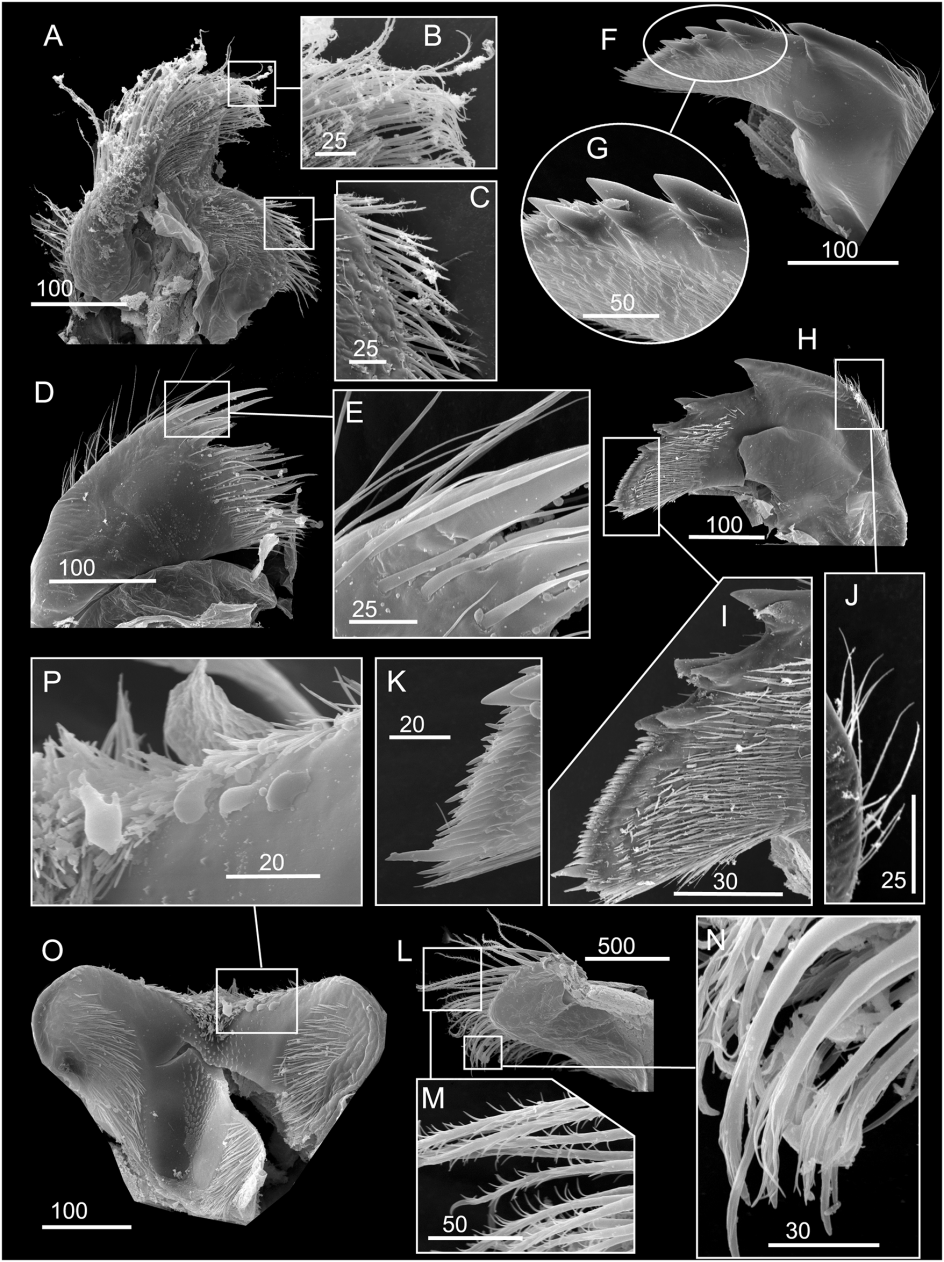
*Chthamalus panamensis* from Avenida Amador, Naos Island, Panama. SEMs. A-C, maxilla. B, C, details of serrulate setae on tips of the two lobes of maxilla. D, E, maxillule, with detail of simple setae (E). F-H, mandible. G, details of the bidenticulate second, third and fourth teeth on the mandible. H-J, Mandible, with detail of pecten (I) and simple setae on outer margin (J). K, Pecten of mandible from additional specimen. L-N, Mandibular palp with details of serrulate setae on tip (M) and on superior margin (N). O, P, Labrum with detail of cutting edge (P). Scale bars in μm.

Figs 2E and 2F and 18–22

***Chthamalus panamensis* Pilsbry, 1916:** pp 319–320, Plate 75 fig 2–2e [2]

#### Specimens examined

ANSP(Crustacea)-2008, type collection of 7 syntypes deposited by Pilsbry 1916, Quarantine Island, Panama (= Naos Island), the intact specimen illustrated in Pilsbry’s Plate 75, fig 2a is selected by the present authors as the Lectotype; AJS-1970. 12 specimens, rock ledges, Balboa Statue, Panama City, Panama. 8° 58.1' N, 79° 32' W, 31 Oct 1970; PWG-1, 10 specimens, intertidal rocks, 200 m N of Naos Island on the west side of Avenida Amador, 8°58.12' N, 79° 31.81' W, 3 Feb 1979.

Diagnosis. *Chthamalus* with a deep longitudinal furrow on outer surface of the tergum, scutal margin of tergum straight. Bidenticulate setae absent from cirrus I. Outer surface of anterior ramus of cirrus I with a longitudinal array of setae. Cirrus II, distalmost segment of both anterior and posterior rami bears bidenticulate setae with basal guards, anterior ramus of cirrus III is about 1.5 times longer than the posterior ramus. Tergoscutal flaps orange. COI sequence (Gene Bank Numbers: KU356706—KU356708).

Description. Shell pink to purple in unbleached or uneroded specimens, otherwise white, with six plates (Figs 2E and 2F and 18A and 18B). Surface with ribs extending to the apex. Orifice elliptical. Scutum and tergum articulated slightly obliquely. Scutum triangular, basal margin twice the length of the tergal margin, external surface with horizontal growth lines, and the upper portion often eroded, tergal margin with shallow articular ridge, not extending beyond the tergal margin, occludent margin straight to slightly convex, with teeth on proximal 1/3 of the margin in uneroded specimens (Fig 18C and 18D). Adductor muscle pit oval, not extending to basal margin. Lateral depressor muscle pit deep and smooth (Fig 18D–18F). Tergum triangular to trapezoid, external surface with horizontal striations and a deep longitudinal furrow is visible in the basal region (Fig 18D–18F). Such a furrow can be observed from above through the orifice of uneroded, undissected specimens (Fig 18A and 18B). Inner surface of tergum smooth, scutal margin straight, spur flat and not obvious, depressor muscle crests 3–4 (Fig 18).

The original description of *Chthamalus panamensis* Pilsbry, 1916, (pp. 219–320 and Plate75 fig 2, 2a, 2b, 2c, 2d and 2e) describes only the shell and opercular plates (also see Fig 18B). Pilsbry’s figure of the exterior of the tergum (Plate 75 fig 2e) is closely similar to the exterior of terga in our new material. The following description of the arthropodal characters is based on new material [Avenida Amador, Naos Island].

Quantitative data on segments counts in cirrus I-III were based on five specimens collected from Avenida Amador, Naos Island (PWG-1). Segment counts on cirri IV-VI were based on a single specimen from Avenida Amador, Naos Island (PWG-1) (Table 4). Cirrus I: posterior ramus 5- to 7-segmented, anterior ramus 6- to 8-segmented, outer surface with a longitudinal array of long setae, with almost one seta per segment, serrulate setae common on both rami (Figs 19A and 19B and 20A–20D). Cirrus I without bidenticulate setae (absent from all specimens examined), pappose setae on basipodite (Fig 20E). Cirrus II: posterior ramus 5 to 7 segments, anterior ramus 6 to 8 segments (Figs 19C and 20G), the most distal segment of each ramus with bidenticulate setae with basal guards, serrulate setae common on both rami (Figs 19C and 19D and 20G–20I, Table 4). Pappose setae on basipodite (Fig 20J and 20K). Cirrus III: posterior 12- to 16-segmented, anterior ramus 16- to 22-segmented, 1.4 to 1.5 times the length of the anterior ramus (Fig 19E), intermediate segments bear 3 pairs of long and 1 pair of short setae (Figs 19E and 20L–20N). Cirrus IV: anterior and posterior rami similar in length (Fig 19F), anterior and posterior rami 16 segmented (Figs 19F and 20O and 20P). Cirrus V: anterior and posterior rami 16-segmented (Fig 19G). Cirrus VI: anterior ramus 19-segmented, posterior ramus 20-segmented (Figs 19H and 20Q). The intermediate segments of cirri IV-VI bear three pairs of long and one or two pairs of short simple setae (Figs 19F–19H and 20S and 20R).

Maxilla bilobed, with dense serrulate setae on each lobe (Figs 21A and 22A–22C). Maxillule notched, notch weak, two large setae above notch, 10 setae below notch (Figs 21B and 22D and 22E). Mandible has 4 major teeth, second to fourth teeth bidenticulate, pecten with 21 very small teeth, inferior angle tipped with 3 pointed teeth (Figs 21C and 21D and 22F–22K). Mandibular palp rectangular, with long simple setae on tip and serrulate setae on superior margin (Figs 21E and 21F and 22L, 22M and 22N). Labrum concave, with 6 or 7 small fine teeth on cutting edge (Figs 21G and 21H and 22O and 22P).

Penis without basi-dorsal point.

#### Distribution

Pacific coast of Central America from about 14° N to 8° 15' N (southern limit unknown).

Remarks. Specimens of *C*. *panamensis* collected for the present study agreed with the syntypes of *C*. *panamensis* described by Pilsbry [2] in having a deep groove on the outer surface of the tergum, which is the key diagnostic feature of *C*. *panamensis*. This morphological feature of the tergum was also reported in Pitombo & Burton [12].

The sympatric *C*. *newmani* sp. nov is distinguishable from *C*. *panamensis in situ* by its lack of deep tergal grooves. In addition, it has has brown tergo-scutal flaps while those of *C*. *panamensis* are orange. The flaps are visible in the living barnacle when the orifice is open under water (Fig 2E and 2F). Arthropodal character differences are: Cirrus I of *C*. *newmani* has bidenticulate setae, Cirrus I of *C*. *panamensis* does not. Cirrus III of *C*. *newmani* has equal rami. Cirrus III of *C*. *panamensis* has unequal rami. This feature of cirrus III in *C*. *panamensis* was not reported by Pilsbry [2] or Pitombo & Burton [12].

### Distribution and Zonation of *Chthamalus* spp. in the Eastern Pacific

**Fig 23:**
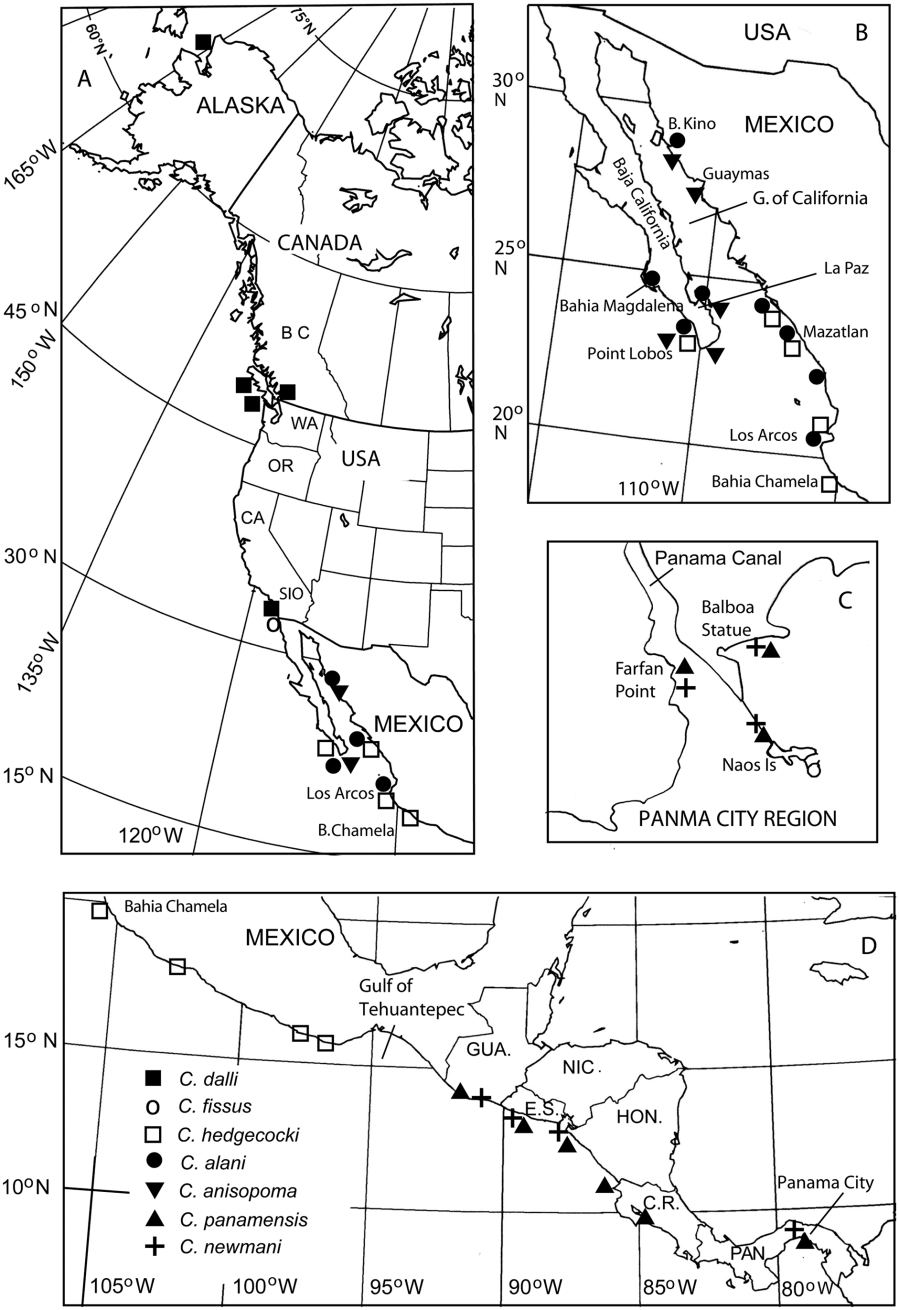
Geographical distribution of species identifications in this study. A. Alaska to northern Mexico; B. Gulf of California; C. Details of Panama City region; D. Southern Mexico to Panama. Locations and other details in Table 1 and S1 Table, North America outline map courtesy Arizona Geographic Alliance, http://geoalliance.asu.edu.

The distribution and intertidal zonation of the chthamalids at the sites studied, on the TEPE78 cruise and from other collections, are listed in in S1 Table, with particular reference to the zonation of other barnacle species and the species’ geographical distribution is shown in Fig 23. At the northern sites, in Alaska, and British Columbia, no species in the *Chthamalus ‘fissus’* group were present and only *Chthamalus dalli* was found. The range of *Chthamalus dalli* extends south to southern California, where it co-occurs with the true *C*. *fissus* and is confined to sheltered crevices on the most exposed rocks on the open coast.

*Chthamalus anisopoma* was mainly restricted to the Gulf of California with a few present on the Pacific coast of Baja California Sur. In the Gulf of California, *C*. *anisopoma* was concentrated above the zone of *Tetraclita* sp., from the main zone at mid-tide level (MTL) to high water spring tide level (HWS), where it was restricted to crevices and under overhangs.

*Chthamalus alani* was found on the eastern side of the Gulf of California from Bahia Kino (28° 51.55' N 112° 1.70' W) southwards. On the western side of the Gulf it was found in the La Paz region. Where *Tetraclita* sp. co-occurred with *C*. *alani* at more exposed sites, *C*. *alani* was restricted to crevices. On the Pacific coast of Baja California the distribution of *C*. *anisopoma* overlapped with that of *C*. *hedgecocki*, at least as far north as Pt. Lobos, 23° 26' N, where *C*. *alani* was also found in sheltered locations.

South of the Gulf of California as far as the Gulf of Tehuantepec, most of the rocky coastal sites are very exposed. *Chthamalus hedgecocki* was the dominant chthamalid present, with *C*. *alani* occurring only in sheltered locations. There was an abrupt species change south of the Gulf of Tehuantepec, with *C*. *panamensis* and *C*. *newmani* replacing C. *hedgecocki* and *C*. *alani*, respectively.

Both *Chthamalus alani* and *C*. *newmani* were generally absent from shores that were exposed to heavy wave action. When present they were found in crevices and on the sheltered side of rocks. In contrast, *C*. *panamensis* and *C*. *hedgecocki* were absent from the most sheltered shores. All four species were most abundant between MTL and high water neap tide level (HWN), often being confined to crevices or shady areas at HWS.

### Molecular Analyses

#### Species identification by enzyme electrophoresis of specimens collected from British Columbia, California and Mexico

*Chthamalus* specimens from north of Scripps Institution of Oceanography and Pt. Lobos, Todos Santos (both on the Pacific coast) were compared, by enzyme electrophoresis, with specimens from British Columbia, to the north, and from La Paz in the Gulf of California and from Isla Sacrificios, Oaxaca, Mexico to the south (Figs 1 and 23, Table 5 and in S2 Table). The enzyme abreviations used are given in Table 2 and allozymes were named by their mobilities with respect to ferritin, which was given a nominal mobility of 100.

**Table 5:** Diagnostic enzyme mobility differences between species of *Chthamalus* in the North-eastern Pacific.

Enzyme	Species of *Chthamalus*						
	*C*. *anisopoma*	*C*. *alani*	*C*. *newmani*	*C*. *hedgecocki*	*C*. *panamensis*	*C*. *fissus*	*C*. *dalli*
Mdh1						83, 93 (96%)	
Mdh2			72 (100%)*		46 (100%)*		
Pgdh	115 (100%)						
Gpdh		80 (100%)**	78 (100%)				68 (99%)
Hk							147, 152, 160 (99%)
Pk							39, 42, 51 (100%)
Pgk		72, 105 ((15%)	97, 110 (14%)				
Pgm	76, 80, 87, 94 (93%)					66, 74 (100%)	
Ark1							93, 106 (100%)
Ark2			61 (45%)	16 (100%)	18, 31, 55 (100%)***		34 (100%)
Ald1						80 (100%)	98 (100%)
En	115 (100%)					128 (100%)	108, 126 (100%)
Gpi			84 (28%)			9, 17, 25, 38 (98%)	
Mpi						88, 100, 109 (100%)	

The enzyme designations are those used in Table 1. Allozyme mobilities were measured with respect to horse spleen ferritin (100), under the buffer conditions in Table 1. Percentages represent the percentage of individuals in the species that had the diagnostic allozyme(s) for the given enzyme.

*These Mdh2 allozymes differentiate *C*. *newmani* from *C*. *panamensis*

**This Gpdh allozyme separates *C*. *alani* from *C*. *newmani*

***These Ark2 allozymes differentiate *C*. *panamensis* from all the other species with the exception of *C*. *anisopoma* and *C*. *fissus*, from both of which *C*. *panamensis* can be separated on the basis of En allozymes

In British Columbia, *C*. *dalli* Pilsbry, a species in the *challengeri* sub-group [33], was the only chthamalid species present and there was no evidence of cryptic species, or significant genetic variation from the specimens of this species sampled there or in California (S2 Table). *C*. *dalli* was the most distinctive of the seven species identified along the Pacific coast of North and Central America. It had unique allozymes for Ark1, Ark2, Ald1 and En in all individuals examined and over 99% of specimens could also be identified as a result of unique allozymes for Gpdh, Hk and Pk (Table 5, S2 Table).

*Chthamalus fissus* Darwin, was common in the Californian samples but was not observed in the specimens screened for electrophoresis from the other sites (S2 Table). The dominant species in the La Paz HWS sample. It was the most distinctive of the six species in the *‘fissus’* group examined, with four of the14 enzymes studied showing unique allozyme(s) in all individuals, Ald1 (80) En (128), Pgm (67, 74),) and Mpi (88, 100, 109), another two Mdh1 (83, 93) and Gpi (9, 17, 25, 38) showed unique allozymes in over 96% of individuals (Table 5). The allozyme relative mobilities are given in parentheses.

*Chthamalus anisopoma* Pilsbry was found in samples screened from Pt. Lobos and La Paz, being particularly abundant at the latter site, but comprising less than 4% of the chthamalids analyzed at Pt. Lobos (Table 6). This species had unique allozyme relative mobilities for Pgdh (115) and En (115) as well as characteristic allozymes, in the majority of individuals, for Gpi (62) and Pgm (76, 80, 87, 94 and 117) Table 5.

**Table 6:** Distribution of the Mexican species of *Chthamalus* examined by electrophoresis.

Site	No. examined	% *C*. *hedgecocki*	% *C*. *alani*	*% C*. *anisopoma*
La Paz HWS	59	0	93	7
La Paz HWN	30	0	17	83
La Paz (all levels)	162	0	80	20
Pt. Lobos	143	93	3.5	3.5
Isla Sacrificios MHWS	20	100	0	0

Two other chthamalids present were more similar morphologically to one another than to the three above species. The species at Isla Sacrificios and the dominant species at Pt. Lobos differed absolutely from the species found at La Paz in the mobilities of 2 of the 14 enzymes tested, Ark2 (16) and Ald1 (64, 100). The species found at La Paz had Ark2 (42, 47, 66, 68, 80), and Ald1 (75, 108). In addition the allozymes of Mdh2, Pgk Pgm and Gpi, could be used to separate > 98% of specimens into one of these two species (Table 5, S2 Table). The dominant species at Pt. Lobos, comprising 93% of the chthamalids present among the specimens sampled by electrophoresis, was absent among the individuals examined from La Paz, while the La Paz species constituted less than 5% of the chthamalids examined from Pt. Lobos (Table 6). The Pt. Lobos and La Paz species both differed in the allozyme mobilities of 5 enzymes, Gpi, Pgm, En, Pgk and Ark2, from *C*. *anisopoma* and *C*. *fissus* (S2 Table). The dominant chthamalid species from Pt. Lobos was identical with 20 frozen specimens, sent by Dr. Hedgecock, collected from Isla Sacrificios (TEPE-14). Since these latter chthamalids were found to be of the same taxon as that found at Bahia Chamela (BC), TEPE78-21, 19°35.0'N, 105° 08.5'W [5], the Pt. Lobos species equates to the BC population of Hedgecock and *C*. *hedgecocki* of Pitombo & Burton [12] that the latter authors also identified from TEPE-14 specimens (Appendix 1 in [12]). *C*. *hedgecocki* could be distinguished from all the other TEPE chthamalids by the presence of the unique allozyme Ark2 (16) in all specimens examined.

The dominant species in the La Paz HWS sample was *Chthamalus alani* nom. nov. (= *C*. *southwardorum* Pitombo & Burton 2007). It could be distinguished from the other five TEP chthamalids in the *‘fissus’* sub-group by the absence of the specific allozymes that characterise these five species and from the closely related *C*. *newmani* by the presence of the allozyme Gpdh 80 in all *C*. *alani* individuals. The distribution of the species in the Mexican samples examined by electrophoresis, is given in Table 6.

#### Species identification by enzyme electrophoresis of specimens collected from Panama

The chthamalids from Naos Island, Panama City and Farfan Point could be separated into two groups on the basis of the enzyme mobility patterns for three of the 16 enzymes tested, namely Mdh-2, Gpdh and Ark-2 (Table 5 and S2 Table). Any one of these enzymes could be used to assign a specimen to one of two presumed species. In addition the allozymes of Gpi, Pgdh, Pk and Ald could be used to separate > 95% of specimens into one of these two groups (S2 Table).

An examination of the specimens in the two groups revealed that they also showed morphological differences. The species dominant at Naos Island had, in live specimens, an orange tergo-scutal tissue flap and a deep longitudinal tergal furrow, “from apex to spur”, as noted by Pilsbry [2] in his description of *Chthamalus panamensis*. The second species dominated at more sheltered sites, such as Avenida Balboa and Farfan Point. It lacked a tergal groove and live individuals had brown tergoscutal flaps. This species is named *Chthamalus newmani* sp. nov. in the present paper.

*C*. *panamensis* had characteristic allozymes for Mdh2 (46), Gpdh (80) and Ark2 (18, 31, 55). In contrast, the comparable allozymes for *C*. *newmani* were Mdh2 (72), Gpdh (78) and Ark2 (42, 61, 68). The specimens from Farfan Point were part of the sample examined by Hedgecock [5] and were referred to by him as FB. Thus *Chathamalus* FB in Hedgecock [5] is *C*. *newmani*. The distribution of the two Panamanian species, in samples examined by electrophoresis, is shown in Table 7.

**Table 7:** Distribution of species of *Chthamalus* in Panama by enzyme electrophoresis.

site	no. in sample	% *C*. *panamensis*	% *C*. *newmani*
Farfan Pt. HW-MTL, sheltered site with turbid water	20	0	100
Panama City, Avenida Balboa HWN—MTL less sheltered than Farfan	35	0	100
Naos Is. site partially exposed from SW	190	76	24

#### Comparison of *Chthamalus* species in the ‘fissus’ sub-group

Side by side electrophoretic comparisons of the enzymes of *C*. *fissus* Darwin, *C*. *alani* nom. nov., *C*. *newmani* sp. nov., *C*. *panamensis* Pilsbry, *C*. *hedgecocki* Pitombo & Burton and *C*. *anisopoma* Pilsbry with *C*. *proteus* Dando & Southward and *C*. *fragilis* Darwin from the Gulf of Mexico and *C*. *dalli* Pilsbry from California showed that all were quite distinct. Nei’s statistics of genetic identity and distance [34] can be used to quantify the average differentiation between species based on allozyme data. These statistics (Table 8) clearly demonstrate the close relationship of *C*. *panamensis* with *C*. *hedgecocki* and of *C*. *alani* with *C*. *newmani*. No specimen showed a pattern for any of these enzymes that would suggest that it might be a hybrid between two species. This was additional evidence that the species are genetically distinct.

**Table 8:** Nei’s genetic distances (above diagonal) and genetic identities (below diagonal, in italics) between Tropical East Pacific *Chthamalus* species.

**species**	***C*. *anisopoma***	***C*. *fissus***	***C*. *hedgecocki***	***C*. *panamensis***	***C*. *newmani***	***C*. *alani***
***C*. *anisopoma***		1.831	0.668	0.622	1.128	0.926
***C*. *fissus***	*0*.*160*		2.531	1.843	2.419	2.375
***C*.*hedgecocki***	*0*.*512*	*0*.*080*		0.262	0.737	0.743
***C*. *panamensis***	*0*.*537*	*0*.*158*	*0*.*769*		0.785	0.691
***C*. *newmani***	*0*.*323*	*0*.*089*	*0*.*479*	*0*.*456*		0.212
***C*. *alani***	*0*.*396*	*0*.*093*	*0*.*476*	*0*.*501*	*0*.*809*	

The observed mean heterozygosity for *Chthamalus hedgecocki* was 0.075 compared with 0.100 *for C*. *alani* and 0.093 for *C*. *fissus* and 0.139 for *C*. *anisopoma*. The mean heterozygosities for the Panamanian species were 0.077 for *C*. *panamensis* and 0.212 for *C*. *newmani*.

#### Mitochondrial DNA analyses

**Fig 24:**
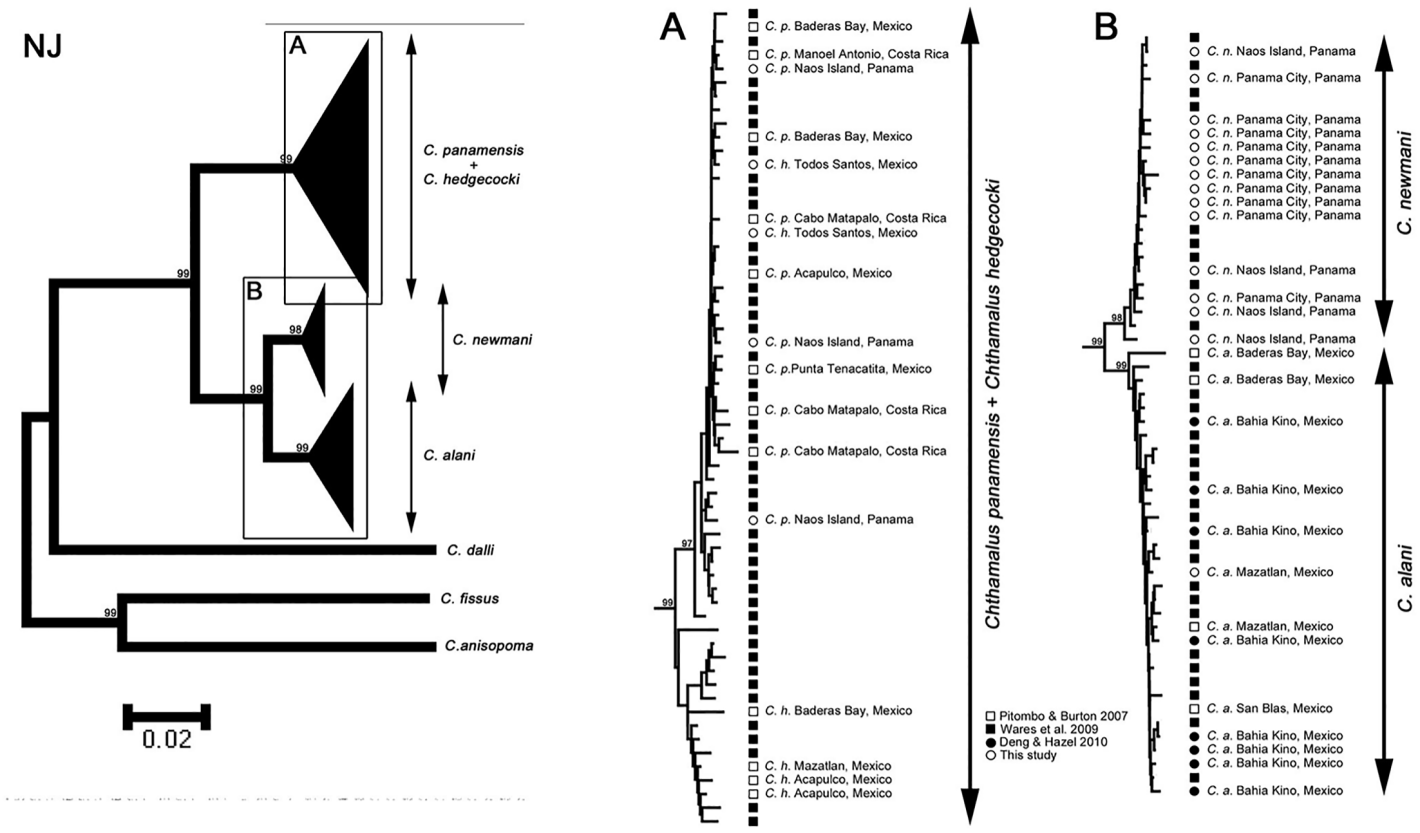
Maximum likelihood phylogeny of mitochondrial gene sequences. Nodal supports are denoted on the corresponding branches for a bootstrap value > 50% for ML analysis. Black squares indicate sequence from Wares *et al*. 2009. White squares indicate sequences from Pitombo and Burton, 2007.

A total of 621 COI nucleotide sequences were obtained (Table 3). Of these, 136 nucleotides were variable and 98 nucleotides were parsimoniously informative. The best-fit nucleotide substitution models for the COI data set were TIM1+G. The genealogical topology showed three reciprocal monophylies, corresponding to *C*. *panamensis* and *C*. *hedgecocki*, *C*. *newmani* sp. nov. and *C*. *alani* (Fig 24). The evolutionary distances based on mean K-2-P distance were 2.1% within *C*. *panamensis* and *C*. *hedgecocki*, 0.6% within *C*. *newmani*, and 0.8% within *C*. *alani*. The evolutionary distances based on mean K-2-P distance between three lineages were 3.6% between *C*. *alani* and *C*. *newmani*, 8.2% between *C*. *hedgecocki* & *C*. *panamensis* and *C*. *newmani*, and 8.6% between *C*. *hedgecocki* & *C*. *panamensis* and *C*. *alani*.

## Discussion

### Morphological Identification of *Chthamalus* Species in the North-Eastern Pacific

The taxonomy of *Chthamalus* species is often confusing, due to the high diversity of species and their similarities in morphology. In the present study it was found that all seven species of *Chthamalus* in the North-Eastern Pacific, *C*. *anisopoma* Pilsbry, 1916, *C*. *alani* nom. nov. (= *C*. *southwardorum* Pitombo & Burton, 2007 in part), *C*. *dalli* Pilsbry, 1916, *C*. *fissus* Darwin, 1854, *C*. *hedgecocki* Pitombo & Burton, 2007, *C*. *newmani* sp. nov.and *C*. *panamensis* Pilsbry, 1916, could be distinguished by: 1) the presence or absence of bidenticulate setae on cirrus I. However, given that in the related Atlantic species, *C*. *proteus* and *C*. *fragilis*, individuals vary in the presence or absence of complex seae on cirrus I and in the presence or absence of basal guards [4] these characters on cirrus I should be used with caution until more specimens from a variety of sites have been examined. 2) the number of segments bearing bidenticulate setae with basal guards on rami of cirrus II. 3) the colour of the tergoscutal flaps. 4) the tergum morphology. 5) the shape and number of teeth on the labrum (Table 9). Among the five species described in detail in this paper, *Chthamalus panamensis* and *C*. *hedgecocki* are morphologically similar, in that both species lack bidenticulate setae on the rami of cirrus I. They both have a longitudinal array of setae on the outer surface of the anterior ramus of cirrus I, as in *C*. *fissus* but unlike the four other *Chthamalus* species in the North-Eastern Pacific. *C*. *panamensis* is unique among the seven northern Pacific species in having a deep longitudinal furrow on the external surface of the tergum and in having the anterior ramus of cirrus III about 1.5 times the length of the posterior ramus. In the other species. the two rami of cirrus III are about equal in length.

**Table 9:** Diagnostic morphological comparisons of arthropodal characters of Chthamalus in TPE.

	*C*. *anisopoma*	*C*. *alani*	*C*. *newmani*	*C*. *hedgecocki*	*C*. *panamensis*	*C*. *fissus*	*C*. *dalli*
Cirrus I	2–3 bidenticulate setae present on the most distal segment of posterior ramus	Bidenticulate setae (with or without basal guards) present on the most distal segment of posterior ramus	Bidenticulate setae (with or without basal guards) present on the most distal segment of posterior ramus	Bidenticulate setae absent Longitudinal array of setae, on outer surface of anterior ramus	Bidenticulate setae absent Longitudinal array of setae on outer surface of anterior ramus	Bidenticulate setae absent. Longitudinal array of setae on outer surface of anterior ramus	Bidenticulate setae on the two most distal segments of posterior ramus
Cirrus II (Posterior ramus)	Bidenticulate setae with basal guards on the 4 most distal segments	Bidenticulate setae with basal guards on the 2 most distal segment	Bidenticulate setae with basal guards on the 3 most distal segments	Bidenticulate setae with basal guards on the 1–2 most distal segment	Bidenticulate setae with basal guards on the most distal segment	Bidenticulate setae with basal guards on the most distal segment	Finely bidenticulate setae (without basal guards) on the most distal segment
Cirrus II (Anterior ramus)	Bidenticulate setae with basal guards on the 2 most distal segments	Bidenticulate setae with basal guards on the1-2 most distal segment	Bidenticulate setae on the 2 most distal segments	Bidenticulate setae with basal guards on the most distal segment	Bidenticulate setae with basal guards on the most distal segment	Bidenticulate setae with basal guards on the most distal segment	Bidenticulate setae (without basal guards) on the two most distal segments
Cirrus III	Ant/Post rami similar in length	Ant/Post rami similar in length	Ant/Post rami similar in length	Ant/Post rami similar in length	Ant ramus 1.4–1.5 times the length of Post ramus	Ant/Post rami similar in length	Ant/Post rami similar in length
Tergo-scutal flaps	White	Orange	Brown	Orange-brown	Orange	Brown	Brown
Tergum	Longitudinal deep furrow absent	Longitudinal furrow absent	Longitudinal deep furrow absent	Longitudinal deep furrow absent	Longitudinal deep furrow present	Longitudinal deep furrow absent	Longitudinal deep furrow absent
Labrum	V-shaped, ~10 teeth on each side of cutting margin	Concave, 6–7 teeth on each side of cutting margin	Concave, 6 teeth on each side of cutting margin	V-shaped, teeth almost invisible	Slightly concave, 6–7 teeth on each side of cutting margin	V-shaped, 6–7 teeth on each side of cutting margin	

*Chthamalus anisopoma*, *C*. *newmani* sp. nov and *C*. *alani* nom. nov. all have bidenticulate setae on the posterior ramus of cirrus I. *C*. *anisopoma* can be distinguished from *C*. *alani* and *C*. *newmani* by having bidenticulate setae without basal guards on the distalmost segments of the posterior ramus of cirrus I, bidenticulate setae with basal guards on the distalmostfour segments of posterior ramus of cirrus II and by having strongly asymmetrical terga and scuta. *C*. *newmani* and *C*. *alani* are morphologically alike and their major differences are the colour of the tergoscutal flaps in life and the different number of segments with bidenticulate setae with basal guards on the posterior ramus of cirrus II. (See Key below and Table 9).

### **Key for identification of *Chthamalus* species in the North-Eastern Pacific**.

1a) Tergum with deep longitudinal furrow on outer surface, anterior ramus of cirrus III approximately 1.5 x the length of the posterior ramus……………….*Chthamalus panamensis*
1b) Tergum without deep longitudinal furrow on outer surface, both ramii of cirrus III of equal length …………………………………………………………………………………………………….. (2)
2a) Left and right scuta and terga strongly asymmetrical, tergoscutal tissue flaps white…………………………………………………………………………………….*Chthamalus anisopoma*
2b) Left and right scuta and terga symmetrical or only slightly asymmetrical, tergoscutal tissue flaps not white …………………………………………………………………………………………… (3)
3a) Cirrus II, with bidenticulate setae without basal guards on the distalmost segment ………………………………………………………………………………………………*Chthamalus dalli*
3b) Cirrus II, with bidenticulate setae with basal guards on the distalmost segment ………………………………………………………………………………………………………………… (4)
4a) Labrum with small, almost invisible, teeth……………………….*Chthamalus hedgecocki*
4b) Labrum with up to 10 teeth on either side of cutting margin………………………………….(5)
5a) Bidenticulate setae with basal guards on the 3 distalmost segments of the posterior ramus of cirrus II ……………………………………………………*Chthamalus newmani* sp. nov.
5b) bidenticulate setae with basal guards on less than 3 of the distalmost segments of the posterior ramus of cirrus II …………………………………………………………………………………….(6)
6a) Tergoscutal flaps dark brown in life, bidenticulate setae with basal guards on the distalmost segment of the posterior ramus of cirrus II ………………………*Chthamalus fissus*
6b) Tergoscutal flaps orange in life, bidenticulate setae with basal guards on the 2 distalmost segments of the posterior ramus of cirrus II …………………….*Chthamalus alani* nom. nov.

### Molecular Analysis

We have shown that two sympatric chthamalid species of the ‘*fissus’* group exist in the Gulf of Panama and that a related pair of species occur along the Mexican coast. These four species were found to be distinct by enzyme electrophoresis and morphology but two of them could not be separated by DNA barcoding.

DNA barcoding is one of the most popular methods for species identification in recent years, especially for people who are not familiar with traditional morphological-based taxonomy. In this study, DNA barcoding revealed three reciprocal monophylies, corresponding to 1) *C*. *panamensis* and *C*. *hedgecocki*, 2) *C*. *newmani* sp. nov. and 3) *C*. *alani*. These four species could also be separated from, *C*. *anisopoma*, *C*. *fissus* and *C*. *dalli* (Fig 24). The DNA barcoding is appropriate to confirm the species status of *C*. *newmani* sp. nov. and *C*. *alani* nom. nov. from the other members of the ‘*fissus’* subgroup. However, in the case of *C*. *panamensis* and *C*. *hedgecocki* in this study, the DNA barcoding is not suitable for species identification. Wares *et al*. [14] used DNA barcoding to identify *Chthamalus* species in the tropical eastern Pacific and then used 16S, nNAKAS and nEF1 genes for phylogenetic analysis. Their DNA barcoding results for *C*. *hedgecocki* and *C*. *panamensis* were similar to the pattern in the present study, in that the bootstrap value and posterior probability in their barcoding analysis was low (63–64 for *C*. *hedgecocki*; 73–87 for *C*. *panamensis*) but the bootstrap value and posterior probability for *C*. *panamensis* plus *C*. *hedgecocki* in the Neighbour-Joining and Bayesian methods were both 100, which is also similar to the pattern in our study. In our results, *C*. *panamensis* and *C*. *hedgecocki*, identified by morphology, were mixed in the same COI clade with low sequence divergence, which suggested that these two species are highly similar in their COI genes. Possible explanations include introgression, as these two species might have hybridised in nature, since they have been reported to be sympatric at two sites in Mexico, sampled in 2002 [12]. Molecular analysis of these two species needs further investigation, using more markers for analysis. Meyers *et al*. [11], based their analysis on the results of Wares *et al*. [14] and suggested that *C*. *panamensis* and *C*. *hedgecocki* separated relatively recently, about 400,000 years ago, and therefore it may be possible for them to hybridise. The above difficulties in establishing phylogenetic relationships, based on mitochondrial genes, between species in the *Chthamalus ‘fissus’* subgroup are analogous to those between species of *Mytilus* in the Atlantic, Baltic and Mediterranean. In the latter genus, haplotype frequency distributions and mtDNA phylogenetic relationships were at variance with systematic hypotheses based on morphological and allozyme data due to hybridization and differential mtDNA introgression [35].

There are clear morphological and allozyme differences between the *Chthamalus* species found on the Pacific coast between Canada and Panama. All seven *Chthamalus* species in this region could be separated by their unique allozymes (Table 5). Nei’s genetic distances for the allozyme data were 0.262 between *C*. *hedgecocki* and *C*. *panamensis* and 0.212 between *C*. *alani* and *C*. *newmani*. In contrast, the genetic distance between *C*.

*hedgecocki* vs *C*. *alani* was 0.743 *and* 0.691 in *C*. *panamensis* vs *C*. *alani*, with the distances between these two outer coast species and *C*. *newmani* being 0.737 and 0.785, respectively (Table 8). This confirms the close relationship between *C*. *alani* and *C*. *newmani* and between *C*. *hedgecocki* and *C*. *panamensis*.

### Identification of Species on the Shore

Considerable plasticity in the shell morphology of cirripeds was recognised by Darwin [1]. Species of *Chthamalus*, especially those of the ‘fissus’ group, can be especially variable in shell morphology, hence the naming of *Chthamalus proteus* (Dando & Southward, 1980) from the Atlantic coast. On the Pacific Coast, *C*. *fissus* exhibits three morphs in opercular form [36] and similarly *C*. *anisopoma* exhibits two [37].

South of the Gulf of California, all *Chthamalus* mentioned in published ecological surveys, even from sheltered shores, have hitherto been called either *C*. *fissus* [38,39] or *Chthamalus panamensis* [40, 41]. On the Californian coast, where *C*. *dalli* and *C*. *fissus* are sympatric [42], the two species are still not usually differentiated on the shore, e.g. [43, 44], even though Miller *et al*.[45] described differences in shell coloration, opercular shape and shell smoothness in the two species from Hopkins Marine Station, Monterey Bay, that allowed the identification of 90%, or more, of specimens to their correct species on the shore. Miller et al. [45] also showed that there were slight differences in size, but not morphology, between the larval stages of the two species. *C*. *fissus* has also frequently been erroneously stated to be present in the Gulf of California [46].

**Fig 25:**
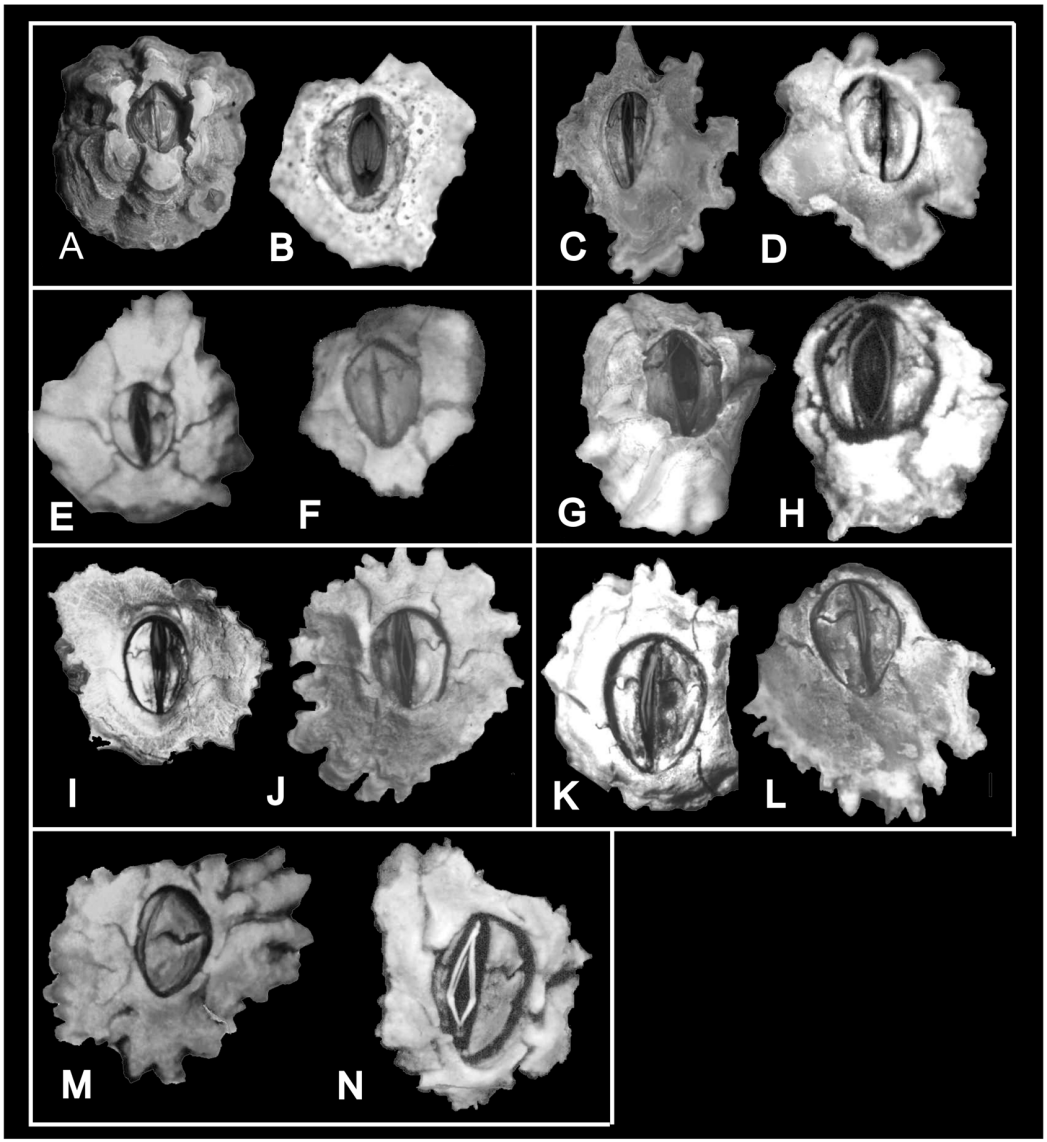
Field photographs of whole barnacles of *Chthamalus* species from the Eastern Pacific. *C*. *dalli*, Cape Thompson; B. *C*. *dalli*, Dillon Beach, CA; C. *C*. *fissus*, Pacific Grove, CA; D. *C*. *fissus*, La Jolla, CA; E, F. *C*. *alani*, La Paz, Mexico; G. *C*. *newmani* Panama City; H. *C*. *newmani*, Naos Is., Panama; I. *C*. *hedgecocki*, Bahia Chamela, Mexico; J. *C*. *hedgecocki*, Pt. Lobos, Mexico; K. *C*. *panamensis*, Naos Is., Panama; L. *C panamensis*, Panama City; M, N. *C*. *anisopoma* La Paz, Mexico.

Although specimens of *Chthamalus* on the Pacific coast of North and Central America can be identified to species by dissection or by enzyme electrophoresis or DNA sequencing, a guide is needed for identification in the field by shore ecologists. Individual plasticity in shell morphology, due to environmental factors, shell crowding, shell erosion, shell fouling and changes in shape with size, means that not all specimens can be identified *in situ*. However, the majority of adult specimens can be identified using the comparative features shown in Table 10 and Figs 2 and 25. These include shell colour, the presence or absence of a crenulated margin, the shape of the opercular opening, the presence of a tergal groove and the colour of the exposed part of the tergoscutal tissue flaps. The latter can be viewed after splashing the barnacles with seawater in order to encourage the barnacles to open. The most difficult species to separate, using shell characters, *C*. *alani* and *C*. *newmani*, may be distinguished by flap colour (Table 10, Fig 2).

**Table 10:** Guide to the field identification of adults of species of *Chthamalus* on the Pacific Coast of North and Central America.

Species	Shell colour	Tergo-scutal flap	Tergal groove	Parietal plates	Orifice shape
*C*. *dalli*	off-white to pale brown	dark brown	absent	margin smooth	angular at carinal end (80–102° mean 93°)
*C*. *fissus*	grey-brown	dark brown	absent	margin crenate	rounded at carinal end, orifice shape variable, can be slit-shaped
*C*. *anisopoma*	white	white	absent	margin crenate, plates often strongly ribbed	oval
*C*. *hedgecocki*	pink-purple in young and non-eroded specimens	dull orange-brown	slight or absent	typically with crenate edges	egg-shaped
*C*. *panamensis*	pink in young and non-eroded specimens	bright orange	deep	smooth, to crenate at edge, ribs do not extend to apex	some egg-shaped but most angular at carinal end (80–140° mean 110°)
*C*. *alani*	white	orange-brown	absent	margin smooth to crenate, any ribs do not extend to apex	kite-shaped to hexagonal, with some rounding at corners (angle 70–113°, mean 91°, at carinal end)
*C*. *newmani*	white	dull brown	absent	margin frequently smooth, occasionally crenate at the edge	kite-shaped to hexagonal, with some rounding at corners (angle 80–105°, mean 93° at carinal end)

Two Atlantic coast species, *Chthamalus proteus* and *C*. *fragilis*, especially the former, could potentially colonize the Pacific coast in the future. These species both have brown shells, in non-eroded specimens, and brown tergoscutal tissue flaps with, usually, a lighter brown edge and can thus be distinguished from the Pacific coast species. A guide to identification of the west-Atlantic species is given by Dando & Southward [4].

### Ecology and Distribution

*Chthamalus* species often occur in sympatric species pairs: one preferentially occupying more sheltered shores and the other found at more wave-beaten sites [47]. On the coast of California *C*. *fissus* is dominant in protected sites while *C*. *dalli* dominates on more wave-beaten shores [48]. This is also true for the species within the *‘fissus’* subgroup, with *C*. *alani* nom. nov. and *C*. *newmani* sp. nov. being the sheltered shore species and *C*. *hedgecocki* and *C*. *panamensis* being the equivalent exposed shore species. In Panama, Laguna [9] found that *C*. *panamensis* was the dominant barnacle of the upper rocky shore at both coastal sites and on offshore islands, but was only present in the higher salinity areas of estuaries.

The two sympatric species pairs, *C*. *panamensis* with *C*. *newmani* and *C*. *hedgecocki* with *C*. *alani*, showed a similar ecological separation to that shown by another sympatric pair in the NE Atlantic, *Chthamalus stellatus* with *C*. *montagui* [18, 47, 49]. In all three pairs the first mentioned species preferentially occupies the more open coast sites whereas the second species is found in more sheltered situations including estuaries and sheltered inlets. Similarly, the three open coast species had low mean enzyme heterozygosities (0.075–0.087) compared with 0.100–0.212 for the sheltered coast species. *C*. *anisopoma*, with a mean heterozygosity of 0.139, would come within the sheltered coast group and is confined to the Gulf of California and sheltered sites on the Pacific Coast of Baja California Sur. The higher mean heterozygosities in the sheltered-shore species are probably an effect of the more variable inshore environment, for example in temperature, salinity and turbidity, resulting in a more heterogeneous population.

A similar situation occurs in the Gulf of Mexico, with the equivalent species pair being *C*. *proteus* and *C*. *fragilis* (Dando & Southward, 1980). Like *C*. *fragilis*, *C*. *alani* and *C*. *newmani* (but not *C*. *hedgecocki* and *C*. *panamensis*) can be found on mangroves. Records of *C*. *panamensis* on mangroves in low salinity areas, e.g. [50], almost certainly refer to *C*. *newmani*. The distribution of the species at different tidal heights at the studied locations is detailed in SI Table. In the intertidal, *Chthamalus hedgecocki* and *C*. *panamensis* preferentially occur on wave-beaten rocks, with their major zone being from MTL to HWN, with lower densities up to EHWS. In contrast, *C*. *alani* and *C*. *newmani* were scarce at such sites, dominating the shore at sheltered sites between MTL and HWN, with somewhat lower densities up to HWS and to EHWS in sheltered crevices. The latter two species occur in sheltered locations, such as in crevices among the *C*. *hedgecocki* at Pt. Lobos, Baja California and inside the lagoon on mangroves at Estero el Zapote, El Salvador. They occur in sheltered locations, including those with higher turbidity and/or lower salinity than open coast positions, although apparently not being as tolerant of low salinities as *Amphibalanus amphitrite*, as suggested by the distribution of *C*. *alani* in Pichilingue Creek La Paz, although the salinity tolerance of the Chthamalus species has not been investigated. In the high intertidal, the *Chthamalus* spp. overlap with *Microeuraphia imperatrix* and *M*. *eastropacensis*, especially in sheltered depressions and crevices. However, the degree of exposure to waves and the presence of sheltered crevices and gulleys is a better guide than shore height to the distribution of the *Chthamalus* species.

We do not have enough detailed observations of the distribution of the exposed and sheltered coast forms where they both occur in sufficient numbers to examine the detailed distributions between the species pairs. In Panama City, on the shore protected from the prevailing SW swells by the San Felipe promontory, below the statue to Vasco Nuñez de Balboa in 1970, a site since infilled, the *Chthamalus* present at MTL-HWS consisted almost entirely of *C*. *newmani*. At Paitilla, 2 km to the west near the Club Union de Panama, the zone of *Chthamalus* lies above the LW zone of hydroids and bryozoans but below a several metre wide zone of *Tetraclita stalactifera panamensis*, *Amphibalanus amphitrite*, *B*. *inexpectatus* and *B*. *tintinnabulum*, i.e. below MTL [51]. It is likely that this *Chthamalus* zone consists of *C*. *panamensis*, although the sheltered-shore species has been reported alongside *C*. *panamensis* at Paitilla Point [12]. North of Naos Island, on the west side of the Avanida Amador causeway, a partially exposed site, both *C*. *panamensis* and *C*. *newmani* occured side-by-side in the main barnacle zone at MTL, in a ratio of approximately 3:1. In contrast, at the sheltered site of Farfan Point, on the western side of the entrance to the Panama Canal, *C*. *newmani* was dominant, with only occasional *C*. *panamensis*, from EHWS to below HWN.

Studies on intertidal zonation in the Golfo de Nicoya, Costa Rica showed a bimodal distribution of *Chthamalus* in the intertidal zone with chthamalids being present higher on the shore at the most estuarine site at Isla Yuca than at the open coast sites at Montezuma and Agujas [40], suggesting the presence of both sheltered and exposed-shore species. Isla del Coco has both sheltered and exposed shores [52] and at Wafer Bay there is also a bimodal zonation of chthamalids [53].

In the present study of samples collected between 1960 and 1980, we found no overlap between the northern species pair, *Chthamalus alani* nom. nov. and *C*. *hedgecocki*, and the southern pair, *C*. *newmani* sp. nov. and *C*. *panamensis*. The approximately 350 km long sand bar in the Gulf of Tehuantepec separated these two species pairs. As well as a lack of suitable habitat in the Gulf, the strong, dominating, winds are northerly [54] and opposing current systems [55, 56] result in surface water flow out into the ocean. This south-trending current will take planktonic *Chthamalus* larvae away from the coast in the Bay of Tehuantepec, whether they arrive from northern or southern populations. They will be dispersed far from land and have no chance of return within their presumed short planktonic life span of 30–40 days [57]. Although Hedgecock [5] claimed to have found a few of the “BC form of *C*. *fissus*” (= *C*. *hedgecocki*) south of the Bay of Tehuantepec, in the Golfo de Fonseca, these were probaby a mis-identification of *C*. *panamensis*, since Hedgecock did not examine any of the allozymes that we found were definitive characters for separating the two species nor did he examine morphological characters. Laguna [9] found *C*. *panamensis*, but not *C*. *hedgecocki*, at this location.

Within the Gulf of California north of the barrier islands, the only *Chthamalus* species present was *C*. *anisopoma* [22]. This species was mainly distributed at MTL, although height on the shore is partly determined by the rock type, as well as the degree of shade [58]. Along the eastern shore *C*. *alani* nom. nov. first appears from Bahia Kino southwards, a distribution since confirmed by samples collected in 2003 and 2006 [22]. *C*. *hedgecocki* first appeared on the eastern shore from Punta San Miguel, Sonora, southwards. It was found together with *C*. *alani* at Mazatlán, where *C*. *alani* was found in tide pools while *C*. *hedgecocki* occured outside pools in positions of greater exposure [12]. At that time, the western shore of the Gulf of California had not been well sampled north of the La Paz area (S1 Table) and the distribution of these species further north along this shore was uncertain.

The southern limit of the species in the *Chthamalus panamensis* complex, south of Panama, is uncertain. Pitombo & Burton [12] reported *C*. “*southwardorum*” from Peru (Punta Colon, Paita, and Punta Chicama, Trujillo). However, observations on samples collected south of Panama suggest that from Southern Ecuador southwards different, but related, species of *Chthamalus* occur (AJS unpublished studies). The northern limit of the *C*. *panamensis* complex is probably in California close to 32° 52' N, where *C*. *hedgecocki* has been reported (the BC form in Hedgecock [5]). Here, *C*. *fissus* and *C*. *dalli* were the dominant *Chthamalus* species. The overlap between *C*. *hedgecocki* and *C*. *fissus* extends southwards along the length of the Pacific coast of the Baja Peninsula to Cabo San Lucas, 22° 52' N [59]. *C*. *alani* is scarce along this section of coast due to the lack of sheltered shores, the known northern limit for this species being at Bahia Magdalena, 24° 38' N.

*Chthamalus fissus* has a northern limit in the region of San Francisco, 37° 35' N [59]. From north of San Franciso to the Aleutians only a single *Chthamalus* species, *C*. *dalli*, is found [59]. Our allele frequency studies on *Chthamalus* collected from a variety of habitats in British Columbia confirm that sympatric species of *Chthamalus* are absent from this region S2 Table.

The density of *Chthamalus* on the shore along the Pacific coast has been considered to be largely determined by larval supply, so that areas with upwelling and with offshore currents have poor recruitment [39, 60–63]. On a local scale factors such as shading, rock type, surge channels, fresh water run off, predation and overgrowth can be important [38, 39, 44, 56, 64]. Observations on the development of the larvae of the individual species are needed, since Miller et al. [45] have shown that larval stages of *C*. *fissus* cannot develop fully at a temperature of 13°C, suggesting that the distribution of *Chthamalus* species along the coast may be partly determined by the water temperatures required for larval development.

The *Chthamalus* specimens from the coast of Mexico examined in the present study were collected mainly in 1978, with one sample from 1980. No specimens of *C*. *panamensis* were among these Mexican samples. Similarly Laguna [9, 10] did not find *C*. *panamensis* at any site along the Mexican coast in any collection made between 1971 and 1980, including the TEPE-78 collections. The species distributions reported here (Fig 23, S1 Table) thus form a baseline against which future intertidal surveys can be compared. The densities and distributions of *Chthamalus* species have been shown to be particularly sensitive to changes in the environment [65, 66].

Since 1980, there is evidence for the presence of *Chthamalus panamensis* at Mexican sites. Pitombo & Burton [12] found *C*. *panamensis* in 6 collections from Mexico made in 2002, at Punta da Mita, Banderas Bay, Nayarit; Punta Tenacatita, Jalisco and one from Pie de La Cuesta, Acapulco, Guerrero, but not in specimens from the 1978 TEPE collections made at Mexican sites, where only *C*. *hedgecocki* was found. This may indicate a recent northward expansion of *C*. *panamensis* in the tropical Eastern Pacific waters.

Most *Chthamalus* species are confined to the intertidal and they are not common fouling organisms on ships’ hulls, although they have been found above the waterline on ships, barges and floating structures [67–70] as well as on floating plastic debris in mid-ocean [71]. Among species in the *“fissus* group”, *C*. *fragilis* has colonized shores north of Cape Cod, possibly transferred by ships from Carolina in the 1800’s [68]. *C*. *proteus* spread rapidly among Pacific islands from the west Atlantic post-1973 [69, 72] and is expected to reach the Pacific coast of America, if it is not already present [68]. This species, together with *C*. *panamensis* and *C*. *“southwardorum”*, has been found on vessels visiting the Pacific Coast ports of the USA [70].

## Conclusions

Our results provide a pre-1980 baseline for the distribution of *Chthamalus* species along the Pacific coast of North and Central America from Cape Thompson in the subarctic to Panama in the tropics. Seven species were present within this range. *C*. *dalli* and *C*. *fissus*, as already known, reached their southern limits close to the Californa/Mexico border. Five other species inhabited the warmer regions from Mexico to Panama, each with a distinct distribution and habitat preference. Future intertidal surveys can compare results with the data presented here, since changes in the range and relative abundance of barnacle species are known to be useful early indicators of ecosystem change in response to environmental change. Colonization by species from other geographic areas, especially the Atlantic species *C*. *proteus*, may be expected to occur.

## Supporting Information

S1 Table Distribution and intertidal zonation of east Pacific chthamalid species, from Alaska to Panama, with particular reference to other cirripedes. (PDF)

S2 Table Numbers of allozymes and allozyme frequencies in *Chthamalus* species examined by electrophoresis. (XLS)
