# Supplementary material for: Biodiversity and Biogeography of Chthamalid Barnacles from the North-Eastern Pacific (Crustacea Cirripedia)
Source: PLoS One. 2016 Mar 9;11(3):e0149556. doi: 10.1371/journal.pone.0149556 (PMC4784953; doi:10.1371/journal.pone.0149556)
Supplement: S1 Table — (PDF) [file pone.0149556.s001.pdf]

S1 Table

Distribution and intertidal zonation of east Pacific chthamalid species, from Alaska to Panama, with particular reference to other cirripedes.

(The basis of identifications of the species of *Chthamalus* at the sites is identified by superscripts: [1]Sequence studies of mitochondrial COI, [2]Enzyme electrophoresis, [3]Light microscopy and SEM of arthropodal characters, [4]Annotated field and laboratory notes, manuscripts and photographs from Alan Southward (AJS), [5]Identification, by AJS, of ethanol-preserved specimens collected and sent by Kathy DeReimer, [6]Identification, by AJS, of slide-mounted and dried specimens collected by Dora Henry, specimens noted as DH followed by the collection number, now housed at the National Museum of Natural History, Smithsonian Institution, USA), [7]field notes and photographs from PRD, [8]specimens collected by Dennis Hedgecock, [9]specimens collected by Peter Glynn, *Southward, A.J. & Southward, E.C., Arctic 20: 8-20 (1967).

Crowbill Point, Ogoturuk Creek, Cape Thompson, Alaska (68° 06' N 165° 48' W), 20 Jul. 1963[*]

Slabs of fallen rock below cliff
MLLW: 0.4 m zone of isolated barnacles on seaward side of slabs, some crowded in pits and depressions.
all *Chthamalus dalli*.

Jackpine Point, Lighthouse Park, B.C. (49° 19.98' N 123° 16.09' W) 25 Jul. 1980[2, 7]

Rocky intertidal,
all *Chthamalus* were *C. dalli*.

Starboat Cove, B.C. Canada (49° 20.0' N 123° 15.69' W) 25 Jul. 1980[7]

Rocky intertidal
HW: *Chthamalus dalli* 57 dm^{-2}, *Balanus glandula* 58 dm^{-2} *Semibalanus cariosus* 18 dm^{-2}
LW: *C. dalli* 2 dm^{-2}, *Balanus* + *Semibalanus* 156 dm^{-2}

Point Atkinson, B.C. Canada (49° 19.86' N 123° 15.95' W) 25 Jul. 1980[7]

Rocky intertidal
HWS: *Chthamalus dalli* 9 dm^{-2}, *Balanus glandula* 161 dm^{-2}
1m below HWS: horizontal platform, *C. dalli* 64 dm^{-2}, *B. glandula* 159 dm^{-2}
1m below HWS: vertical face, *C. dalli* 19 dm^{-2}, *B. glandula* 150 dm^{-2}
2m below HWS: horizontal platform, *C. dalli* 43 dm^{-2}, *B. glandula* 308 dm^{-2}
3m below HWS: horizontal platform, *C. dalli* 36 dm^{-2}, *B. glandula* 540 dm^{-2}
LW: *C. dalli* 33 dm^{-2}, *B. glandula* 367 dm^{-2}

Tower Beach, Vancouver, B.C. Canada (49°16.36' N 123° 15.52' W) 24 Jul. 1980[7]

Rocky intertidal
HWN: *Chthamalus dalli* 6 dm^{-2}, *Balanus glandula* 144 dm^{-2}
LWS: no *C. dalli*, *B. glandula* was the only barnacle present

Tofino, Vancouver Is. B.C. Canada (49° 9.22' N 125° 54.78' W) 29 Jul. 1980[2,7]

Rocky intertidal,
all *Chthamalus* were *C. dalli*.

Hypophocus Is., Vancouver Is., B.C. Canada (48° 55.8' N 125° 31.6' W) 28 Jul. 1980[2,7]

Rocky intertidal,
all *Chthamalus* were *C. dalli*.

Terrace Beach, Ucluelet, B.C. Canada (48° 55.71' N 125° 22.36' W) 25 Jul. 1980[22,7]

Rocky intertidal
all *Chthamalus* were *C. dalli*.

Mill Bay, Vancouver Is. B.C. Canada (48° 37.88' N 123° 31.75' W) 26 Jul. 1980[2,7]

Rocky intertidal
all *Chthamalus* were *C. dalli*.

Botanical Beach, Vancouver Is. B.C. Canada (48° 31.56' N 124° 26.66' W) 23 Aug. 2013[7]

Wave-exposed rocky shore with large pebble-scour of sedimentary rocks.
Chthamalus dalli scarce at wave-exposed sites and mainly found on sheltered rocks on vertical faces or in crevices.
HWS: no barnacles
0.45 m lower: *C. dalli* 5 dm^{-2}, *Balanus glandula* 23 dm^{-2}
0.7 m lower: *C. dalli* 31 dm^{-2}, *B. glandula* 22 dm^{-2}
0.85 m lower: *C. dalli* 11 dm^{-2}, *B. glandula* 18 dm^{-2}
MHW: wave exposed vertical face, *C. dalli* 8 dm^{-2}, sheltered side of same rock, *C. dalli* 37 dm^{-2}, on horizontal rock platforms all barnacles were confined to deep crevices
MLW: *B. nubilus* and *Pollicipes polymerus*
LWN: *P. polymerus* among large *Mytilus*.

Victoria, Vancouver Is. B.C. Canada, North end of Holland Point (48° 24.63' N 123° 22.72' W) 24 Aug. 2013[7]

Rocky outcrops
Chthamalus dalli was absent from rocks facing waves, unless in crevices but was present on the shoreward side of rocks and scarce at HWS.
EHWS: exposed rocks *C. dalli* and *Balanus glandula* confined to crevices. *Lottia scabra* on vertical faces
HWS: *Pollicipes polymerus* in deep, draining, crevices, with a few *C. dalli* on the rock-face above
MHWS W-facing rock crevice: *C. dalli* 4 dm^{-2}, *B. glandula* 62 dm^{-2}
0.1 m lower: *C. dalli* 9 dm^{-2}, *B. glandula* 144 dm^{-2}
0.15 m lower: *C. dalli* 76 dm^{-2}, *B. glandula* 58 dm^{-2}
0.1 m lower: *C. dalli* 92 dm^{-2}, *B. glandula* 85dm^{-2}
0.1 m lower: *C. dalli* 78 dm^{-2}, *B. glandula* 23 dm^{-2}
1.0 m lower on horizontal platform: *C. dalli* 56 dm^{-2}, *B. glandula* 16 dm^{-2}
0.3m lower to LW: zone of *B. nubilus*

Victoria, Vancouver Is. B.C. Canada (48° 24.5'N 123° 22.6'W) 27 Jul. 1980[2,7]

Rocky intertidal

all *Chthamalus* were *C. dalli*.

Victoria, Vancouver Is. B.C, SE end of Beacon Hill Park ((48° 24.38' N 123° 21.85' W) 18 Aug. 2013[7]

Rocky intertidal, W-facing, semi-sheltered rocks.

HHWS: C*hthamalus dalli* 18.5 dm^{-2} *Balanus glandula* 2.9 dm^{-2}, barnacles concentrated under overhangs and in crevices.

MHWS: C. *dalli* 35.5 dm^{-2} *B. glandula* 15.3 dm^{-2} the *C. dalli* preferentially occupied the depressions.

MHW: vertical surface C. *dalli* 31.1 dm^{-2} *B. glandula* 21.1 dm^{-2}

MHW: horizontal surface C. *dalli* 34.5dm^{-2} *B. glandula* 3.7 dm^{-2}

Rocks north of Scripps Institution of Oceanography, California (32° 52.27' N 117° 15.20' W) 01 Nov. 1978 [2,4]

HW *Chthamalus fissus* 800 dm^{-2}

MTL *C. fissus* 680 dm^{-2} *C. dalli* 28 dm^{-2}

LW *C. fissus* 4.5 dm^{-2} *C. dalli* 12 dm^{-2}

Bahía Magdalena, Baja California Sur (24° 38' N 112° 08' W)[6]

Bay rocks protected from the Pacific waves by barrier islands

(All chthamalids were *Chthamalus alani* DH7288-7290; DH 8396, DH8397, DH8399)

Pt. Lobos, Todos Santos, Baja California Sur TEPE78-34 (23° 24.5 N 110° 14' W)[4]

Rocky headland 17 Apr.1978

HWS: in crevices, a few *Microeuraphia imperatrix* (≤ 20 dm^{-2}) together with *C. hedgecocki* (92 dm^{-2})

20 cm below: upper limit of the main *Chthamalus* zone with *C. hedgecocki* (172 dm^{-2}) and *C. anisopoma* (12 dm^{-2})

50 cm below HWS: *C. hedgecocki* (140 dm^{-2}) and *C. anisopoma* (8 dm^{-2})

Scattered *Tetraclita stalactifera* occurred just below the main *Chthamalus* zone.

LW: some *Megabalanus* sp.

04 Nov.1978[1,2,3,4] AJS-3

HWS: in crevices, *C. hedgecocki* (160 dm^{-2})

HHWN: *C. hedgecocki* (240 dm^{-2})

LHWN: *C. hedgecocki* (250 dm^{-2}) + *C. anisopoma* (15 dm^{-2})

N.B. The predominantly *C. hedgecocki* population contained 5% *C. alani*. *C. alani* was found in sheltered crevices or hollows in the *C. hedgecocki* zone.

Cabeza Belleza, Baja California Sur TEPE78- 29 (22° 53.78' N 109° 52.03' W) 16 Apr. 1978[4]

Rocky point 3 km E of Cabo San Lucas

HWS: in deep crevices, a few *Microeuraphia imperatrix* (≤ 2 dm^{-2}) above *C. anisopoma* that was restricted to the lichen zone.

Gulf of California (sites from S to N)

Ocean front, Mazatlan, Sinaloa, Mexico. TEPE78-36 (23° 11.5' N 106° 25.7' W) 19 Apr. 1978[3, 4]

Boulders with barnacles present under stones and in gullies
EHWS: *Microeuraphia imperatrix* (20-30 dm^{-2}) and *Chthamalus alani* mixed with some *C. hedgecocki* (360 dm^{-2})
HWN: *C. alani* + *C. hedgecocki* (>450 dm^{-2})
LWN: *C. hedgecocki* + a few *C. alani* (100 – 240 dm^{-2})

Bahia de las Olas Altas, Mazatlan, Sinaloa, Mexico TEPE78-38 (23° 11.9' N 106° 25.7'W) 19 Apr. 1978[4]

Platform of concrete and volcanic rock
HWS: *Microeuraphia imperatrix*
HWN: *Chthamalus hedgecocki* and *C. alani*

Punta Camaron, Mazatlan, Sinaloa, Mexico TEPE78-39 (23° 14' N 106° 26' W) 19 Apr. 1978[4]

Platform of volcanic conglomerate with extensive flats and rock pools
HWS: 0.3 m zone of *Microeuraphia imperatrix* (up to 200 dm^{-2}) with a few young *Chthamalus* (8 dm^{-2}).
MHWN to MTL: dense zone of *C. hedgecocki* + *C. alani* (290 – 400 dm^{-2}).
LWN: mainly *C. hedgecocki* (600 – 700 dm^{-2}), many juveniles

Large vertical blocks of conglomerate
HWS: 0.3 m wide band of *M. imperatrix* (up to 400 dm^{-2})
HWN: *C. hedgecocki* + *C. alani* (360 dm^{-2}).
LWN: *C. hedgecocki* + *C. alani* including juveniles (100-250 dm^{-2}).

Punta San Miguel, Sonora, Mexico TEPE78-40 (23° 39.4' N 106° 48.6' W) 20 Apr. 1978[4]

Volcanic conglomerate in sand
HWS: *Microeuraphia imperatrix* under ledges (4 dm^{-2})
HWN – LW: *C. alani* + *C. hedgecocki* (100-360 dm^{-2}), 15 *C. hedgecocki*, 8 *C. alani* in sample

La Paz Bay TEPE78-33 (24° 06.6' N 110° 21.6' W) 17 Apr. 1978[4]

Mangroves on mudflat
Dead mangroves: a 30 cm barnacle zone, the upper 10 cm contained *C. alani* only, below this *C. alani* was mixed with a few *Amphibalanus amphitrite*.
Live mangroves: the barnacle zone comprised *C. alani* with a line of *B. amphitrite* at the lower end.

No *C. anisopoma* were observed at this site

Montana Rock, Isla Cerralvo TEPE78-25 (24° 08' N 109°48' W) 14 Apr. 1978[4]

Exposed south-facing site

HWS, 100-150 cm above CD: bare rock with a few *Littorina* sp., a few *C. anisopoma* in crevices but no *Microeuraphia*
20-80 cm above CD: main zone of *C. anisopoma* with a few small limpets and vermetids. *C. anisopoma* densities: 252 dm^{-2} towards top of zone, 232 dm^{-2} towards base
Overlapping the base of this zone were large *Megabalanus tintinnabulum* and *Tetraclita* sp.with *Lithothamnion*, grapsids and limpets.
0-20 cm above CD: *Megabalanus* sp. and *Acmaea* sp.

No *C. alani or C. hedgecocki* were observed at this station

Isla Cerralvo TEPE78-24 (24° 08' N 109°49' W) 14 Apr. 1978[4]

Partly sheltered reef, unshaded rocks
90-110 cm above chart datum (CD): *Microeuraphia* sp. in crevices with a few *C. anisopoma*
80–90 cm above CD: *C. anisopoma* was dispersed at a of density 6 dm^{-2}
55-80 cm above CD: main zone of *C. anisopoma* 140 dm^{-2}
50 cm above CD: *C. anisopoma* 10 dm^{-2}
45-65 cm above CD: zone of *Tetraclita stalactifera* (40 dm^{-2}) *overlapped with C. anisopoma*
10- 45 cm above CD: *Megabalanus tintinnabulum* with *Lithothamnion*
10 cm above CD: *Megabalanus* sp. 2-4 dm^{-2}
>10 cm below – 10 cm above CD: *Strongylocentrotus*

Inside of reef in the shade
90-110 cm *Microeuraphia imperatrix* (16 dm^{-2}) and *C. anisopoma* (20 dm^{-2})

No *C. alani* or *C. hedgecocki* were observed at this station

La Paz, NW end of promenade TEPE78-35 (24°10.35' N, 110°18.42' W) 17 Apr. 1978 [3, 4]

Stones and boulders on shore
HWS: large boulder with mainly *Chthamalus alani* and 1 *Microeuraphia imperatrix*
LW: *C. anisopoma* (up to 100 dm^{-2}) and C. *alani*+ *C. hedgecocki* (up to 20 dm^{-2})

La Paz beach AJS 2 (24° 12.44' N 110° 1.06' W) 03 Nov. 1978 [2,4]

Rocks at beach edge 5 km N of town
EHWS: a few *Microeuraphia imperatrix*, but not more than 4 m^{-2}, in crevices or under stones
HWS: *C. alani* in crevices above main barnacle zone
HWN: mainly *C. anisopoma* with some small and a few older *C. alani* in drier places or on projecting rocks.

Pichilingue Creek, N. of La Paz TEPE78-33 (24° 14.8' N 110° 18.8' W) 17 Apr. 1978[4]

Mangroves at Head of creek: *Amphibalanus amphitrite amphitrite* only.

HW, half way along creek: no barnacles but some *C. anisopoma* just below this
HW –0.4 to –0.5 m: *C. alani* on sides of, and underneath, boulders.

Pta. Arranca Cabellos, Baja California Sur (24° 21.1' N 110° 17.8' W) 3 Nov. 1978[4]
Wave-beaten rocks
HWS: *Tetraclita* sp. with *C. anisopoma* below this. A few *C. alani*, mainly in crevices.

Miramar, Guaymas, Sonora, Mexico TEPE78-46 (27° 55.6' N 110° 57.1' W 26 Apr. 1978[4]
Boulders on channel into lagoon
Mainly *C anisopoma* with some *B. inexpectatus* and a few *A. amphitrite*.

Bahia Bacochibampo, Guaymas, Sonora, Mexico TEPE78-47 (27° 55.7' N 110° 58.0' W) 26 Apr. 1978[4]
Towards the mouth of the Bay, N. side on fishing beach
Large boulders on the upper shore: *C. anisopoma* only
Below these: a few *Balanus inexpectatus*.

Bahia Kino, Sonora Mexico TEPE78-44 (28° 51.5' N 112° 1.7' W) 25 Apr. 1978[4]
Volcanic rock, slightly turbid water.
EHWS: *C. alani* in crevices in shade + some *Tetraclita* sp.
HWS in shade: *C. alani* (70-280 dm^{-2}), *C. anisopoma* (40 dm^{-2}), *Tetraclita* (1-4 dm^{-2})
HWS in sun: *C. alani* (270 dm^{-2}), no *C. anisopoma* and only dead *Tetraclita*
HWN: *C. alani* (200 dm^{-2})), *C. anisopoma* (80 dm^{-2}) + zone of *Tetraclita* (most dead)
MTL: *C. alani* (120 dm^{-2}), *C. anisopoma* (40 dm^{-2}), lower limit of *Tetraclita* zone
HLWN: *C. alani* (10-50 dm^{-}2), main zone of *C. anisopoma* (160 dm^{-2})
LLWN: few *C. anisopoma*, overgrown with algae and mytilids.

South of the Gulf of California

San Blas, Nayarit, Mexico (21° 32.5' N 105° 14.4' W) 1980[8]
Dry sample on rocks from the harbour, young *Chthamalus alani*

Sayulita, Nayarit, Mexico TEPE78-42 (20° 52.2' N 105° 26.7' W) 22 Apr. 1978[4]
Boulders on edge of lagoon
MTL – HWN: mixed zone of *C. alani* and *C. hedgecocki* (5.2-5.5 dm^{-2})
LW: young *Chthamalus* with dense *Tetraclita* (up to 15 dm^{-2}) and *Megabalanus tintinnabulum*

Los Arcos, Nayarit, Mexico TEPE78-43 (20° 32.7' N 105° 17.2' W) 23 Apr. 1978[4]
Boulders at W end of Bay
EHWS: *Microeuraphia imperatrix* (up to 40 dm^{-2})
HWS: mixed zone of *C. alani* and *C. hedgecocki* (100-620 dm^{-2})

HWN: *C. alani* and *C. hedgecocki* (220 dm^{-2})
LWN: in wave-exposed areas patches of *Tetraclita stalactifera*, and of *Balanus peninsularis*.

Bahia Chamela, Jalisco, Mexico TEPE78-21 (19° 35' N 105° 08' W) 12 Apr. 1978[4]
Exposed rocky point.
HWS; 0.5 m zone of *Littorina* and *Neritia* spp., below this was a 1.5 m zone of *Chthamalus hedgecocki*, with *Siphonaria* and *Acmaea* spp., with a few *Microeuraphia imperatrix* in crevices in a 0.2 m deep zone overlapping the upper part of the *Chthamalus* zone. A 30 cm zone of *Lithothamnium* occurred 30 cm below the *Chthamalus* zone.

Isla Grande (Ixtapa Island), Ixtapa, Guerro, Mexico TEPE78-17 (17° 40.6' N 101° 39.4' W) 12 Apr. 1978[4]
Beach on south side
only a few barnacles, mainly concentrated in a shady north-facing crevice on the western side of the beach at + 1m where *Microeuraphia imperatrix* occurred deep in the crevice at 40 dm^{-2} and *Chthamalus hedgecocki* occurred on the outer side of the crevice at 5-10 dm^{-2}.

Puerto Escondido, Oaxaca, Mexico (15° 51.5' N 97° 3.9' W)[6]
Exposed coast site
2 specimens, both *Chthamalus hedgecocki* (DH 10020, DH 10022)

Coral Bay, near Isla Sacrificios, Oaxaca, Mexico TEPE78-16 (15° 41.8' N 96° 13.6' W) 08 Apr. 1978[4]
Rocks on W side of semi-enclosed bay with a coral platform at LWN, traverse down rocky shore (heights are given above chart datum)
Splash zone 2.8 m: littorinids in crevices (10-12 dm^{-2})
2.6 m: upper limit of *Microeuraphia imperatrix* (15 dm^{-2})
2.25 m: middle of zone of *Microeuraphia imperatrix* (20 dm^{-2}) and upper limit of *Chthamalus hedgecocki* (8 dm^{-2})
1.9 m: lower limit of *Microeuraphia imperatrix* (2 dm^{-2}) + some *C. hedgecocki*
1.7 m: upper limit of main zone of *C. hedgecocki*
1.6 m: *C. hedgecocki* (120 dm^{-2})
1.2 m (HWS): *C. hedgecocki* (360 dm^{-2}), upper limit of rapaninid gastropods
1.1 m; *C. hedgecocki* (470 dm^{-2}) + small *Siphonaria* (5-11 dm^{-2}) + rapaninids (2-4 dm-2)
0.65 m: upper limit of *Lithothamnion*
0.6 m: *C hedgecocki* (30 dm^{-2}) + *Siphonaria* (25 dm^{-2})
0.5 m: *Siphonaria* (25 dm^{-2}) + *Acmaea* (1 dm^{-2}), no barnacles
level increased to 0.45 m lottiid gastropods on seaward edge of rock
0.5 m: a few *Tetraclita stalactifera* (<0.1 dm^{-2})

Isla Sacrificios, Oaxaca, Mexico TEPE78-14 (15° 41.2' N 96° 13.9' W) 07 Apr. 1978[2,4]
Rocks at western tip of Island
HWS: *Microeuraphia imperatrix*
LW to HWN: *Chthamalus hedgecocki*

LW: *Megabalanus sp*.

Puerto San Jose, Guatamala (13° 55.2' N 90° 47.1' W) 07 May 1961[6]

12 specimens examined from towards the base of the pier: 7 *Chthamalus panamensis* (DH 3875, DH7470, DH7472a, DH7472b, DH7473 + 2 others); 5 *C. newmani* (DH 3874, DH 3876, DH7474, DH7475, DH7476, DH7477 + 1 other)

LW on *Megabalanus tintinnabulum*: all *C. newmani* (DH3873, DH3874, DH3876 + unnumbered samples)

Estero el Zapote, Barra de Santiago, El Salvador (13° 42.3' N 90° 01.3' W) 02 Mar. 1978[5]

Mangroves (*Rhizophora*) in lagoon.
Chthamalus newmani

La Shalpa, El Salvador (13° 29.9' N 89° 28.9' W) 06 Sep. 1978[5]

MTL horizontal plaform at wave-exposed coastal site
93 specimens, 94% *Chthamalus panamensis*, 6% *C. newmani*

Gualpirque, El Salvador (13° 14.0' N 87° 50.8' W) 20 Aug. 1978[5]

MLW on shells of *Tetraclita panamensis*: 4 *Balanus inexpectatus*, 42 *Chthamalus panamensis*, 50 *C. newmani*
MTL on shells of *Brachydontes* sp.: 18 *C. panamensis*, 58 *C. newmani*
on shells of *Thais* sp.: 4 *C. panamensis* 96 *C. newmani*

Isla del Tigre, Golfo de Fonseca, Honduras TEPE78-8 (13° 15' N 87° 38.2' W) 04 Apr. 1978[4]

Pta. Parada on volcanic rocks, sheltered by outer island.
HWS: 0.5 m zone of *Microeuraphia eastropacensis* (1-2 m^2 on verticals and approximately 1000 m^2 in crevices) with *Littorina* sp.
HWN-LWN: 1.5 m zone of *C. newmani* 200-300 dm^{-2} with a few [5%, Hedgecock (1979)] *C. panamensis*. 0.5 m below the top of this zone the chthamalids were mixed with *Balanus inexpectatus*
0.5 m above LWN: zone of oysters

Key Pt., Bahia Murciélagos, Costa Rica TEPE78-7B (10° 53.4' N 85° 54.2' W) 03 Apr. 1978[4]

Exposed rocky point.
HWS: *C. panamensis* in cavities and runnels only. A few *Microeuraphia imperatrix* were found at some sites
-0.5 m (HWN): small *C. panamensis* in shaded places (60 dm^{-2}) and upper limit of limpets.
-1.0 m: limpet density 2.5 dm^{-2}
-1.5 m (LW): no barnacles, dead *Lithothamnion*

Golfito, Costa Rica (8° 38.2' N 83° 10.1' W)[6]

Chthamalus panamensis (DH 8299)

Farfan Point, Panama (TEPE78-1) 8° 56.1' N 79° 34.2' W) 30 Mar. 1978[1, 2,3, 4]

Rock platform and vertical face
EHWS: scattered *C. newmani* mainly in crevices and shade.
HWS: *C. newmani* covered 40% of rock surface in the shade.
Just above MHWS: *Microeuraphia imperatrix* and *M. eastropacencsis* formed a scattered population on bare rock but were more concentrated in crevices with *C. newmani*.
MHWS: The euraphid species became well mixed with *C. newmani*
HWN-MTL: *C. newmani* covered 60-70% of the rock surface. A few *C. panamensis* were also present.
Below MTL: *Balanus inexpectatus* dominated but was mixed with *C. newmani*.

Panama City, Panama (8°57.9' N 79° 32' W) 31 Oct. 1970, 07 Jun. 1979[1,2,3,9] PWG 3

Sheltered site off Avenida Balboa opposite Balboa Statue
EHWS-MHWS: *Microeuraphia imperatrix* and *M. eastropacensis* abundant in hollows and crevices, where occasional specimens of *C. panamensis* with *C. newmani* were also found, but they occurred in much lower numbers on flat surfaces and elevated areas.
HWS-HWN: small numbers of *C. panamensis* with more numerous *C. newmani*
HWN – just above MTL: dense belt of *C. newmani* with very occasional *C. panamensis*
MTL –MLWN: *C. newmani* at a lower density. From MTL downwards *C. newmani* was mixed with *Balanus inexpectatus* Pilsbry and occasional *Tetraclita panamensis* Pilsbry. Cirripedes were displaced by a belt of sabellariid tubes below MLWN.

Naos Island, Panama (8° 55.07' N 79° 32.11' W) 07 Feb. 1979, 07 Jun. 1979[1,2,3,9] PWG 2

Partially sheltered site 200 m N of Naos Island on the west side of Avenida Amador.
EHWS-MHWS: *Microeuraphia imperatrix*
HWN-MTL: *Chthamalus panamensis* and *C. newmani*
MHWN: *C. panamensis* and *C. newmani* sparser interspersed with areas of bare rock and with some *Tetraclita panamensis*
MTL: *Catophragmus pilsbryi* at outer edge of rocks

La Chorrera Beach, Panama (8° 51.0' N 79° 44.35' W)[6]

3 specimens examined, all *Chthamalus newmani* (DH 4513, DH 4513a, DH 4513b)

San Jose Island, Panama (8° 15.4' N 79° 05.0' W)[6]

7 specimens examined, all *Chthamalus panamensis* (DH 4475, DH 4472a, DH 4472b)
